# Comparative efficacy of gut microbiota-targeted therapies for motor and non-motor symptoms in Parkinson's disease: a systematic review and network meta-analysis

**DOI:** 10.3389/fmicb.2026.1907410

**Published:** 2026-10-26

**Authors:** Xiaoyuan Yang, Yuanping Li, Qinghong Hu, Xuan Pan, Jinhua Hong, Lu Li

**Affiliations:** Department of Nursing, Jiang Xi Cancer Hospital, Nanchang, China

**Keywords:** fecal microbiota transplantation, microbial ecology, microbiota-gut-brain axis, microecological homeostasis, network meta-analysis, probiotics

## Abstract

**Background:**

Gut dysbiosis may contribute to Parkinson's disease (PD) through the microbiota-gut-brain axis. Probiotics, synbiotics, and fecal microbiota transplantation (FMT) have been studied as potential treatments, but their comparative effects remain uncertain.

**Methods:**

We searched trial registries and electronic databases from inception to April 1, 2026 for randomized controlled trials of gut microbiota-targeted therapies in PD. We performed a frequentist network meta-analysis. Risk of bias was assessed with Cochrane RoB 2, and confidence in the evidence was assessed with CINeMA. Treatment rankings were summarized using SUCRA values.

**Results:**

Nineteen RCTs involving 1,156 participants and five treatment options (FMT, probiotics, synbiotics, routine care, and placebo) were included. Probiotics increased spontaneous bowel movement (SBM) frequency compared with placebo (MD = 1.93, 95% CI: 0.23 to 3.64; SUCRA = 88.3%). In the oral-administration subgroup, probiotics (MD = 2.01, 95% CI: 0.64 to 3.39) and synbiotics (MD = 1.23, 95% CI: 0.01 to 2.45) increased SBM frequency. No active treatment significantly improved MDS-UPDRS III scores compared with placebo, although synbiotics and FMT had higher SUCRA rankings. Evidence certainty was Moderate for oral probiotics on SBM frequency and Low to Very Low for motor function and quality of life.

**Conclusion:**

Oral probiotics improved SBM frequency in patients with PD. Evidence for motor function and quality of life was uncertain, and the higher rankings of FMT and synbiotics should be considered exploratory. Larger head-to-head randomized trials are needed.

**Systematic review registration:**

https://www.crd.york.ac.uk/PROSPERO/recorddashboard.

## Introduction

Parkinson's disease (PD) is a progressive neurodegenerative disorder characterized by motor and non-motor symptoms (Dorsey and Bloem, 2018). Gastrointestinal dysfunction, particularly constipation, may precede motor symptoms (Postuma et al., 2015) and is consistent with hypotheses that α-synuclein pathology can involve the enteric nervous system and the gut-brain axis (Eisenstein, 2026; Horsager et al., 2020). Gut bacteria may also affect levodopa availability; for example, *Enterococcus faecalis* can metabolize levodopa in the intestine (Shen et al., 2026; van Kessel et al., 2019). Probiotics, synbiotics, and fecal microbiota transplantation (FMT) may influence intestinal barrier function, inflammation, microbial composition, and bowel motility (Hong et al., 2022; Duan et al., 2024; Jin et al., 2026; Nabil et al., 2025; Kuai et al., 2021). However, trials have reported inconsistent results, partly because microbial strains, doses, delivery routes, and treatment durations differ (Bertin et al., 2025).

Pairwise meta-analyses cannot compare all available gut microbiota-targeted therapies within one framework (Djulbegovic and Guyatt, 2017; Cipriani et al., 2018). We therefore performed a network meta-analysis to compare their effects on motor symptoms, gastrointestinal outcomes, and quality of life in PD, while accounting for the certainty and limitations of the available evidence (Chaimani et al., 2013; World Health Organization, 2025).

## Methods

This systematic review and network meta-analysis was conducted in accordance with the PRISMA 2020 statement and the PRISMA extension for Network Meta-Analyses (PRISMA-NMA) guidelines. The study protocol was prospectively registered in the PROSPERO international database (registration number: CRD420261380067) to ensure methodological transparency and to minimize the risk of bias (Page et al., 2021; Hutton et al., 2015).

### Search strategy

To identify eligible clinical trials investigating gut microbiota-targeted therapies for Parkinson's disease, we systematically searched primary databases including PubMed, Embase, Web of Science, the Cochrane Central Register of Controlled Trials (CENTRAL), CNKI, Wanfang Data, VIP and CBM. The search captured all available literature from database inception until April 1, 2026. The systematic search was restricted to articles published in either English or Chinese. A team of two reviewers independently conducted the search and screened titles and abstracts against our predefined criteria. Any discrepancies during the selection phase were resolved through internal discussion or by consulting a third reviewer to achieve full consensus.

We also searched conference proceedings from major neurology and gastroenterology organizations, trial registries (ClinicalTrials.gov, WHO ICTRP, and ChiCTR), reference lists of eligible studies and relevant reviews, and Google Scholar citations. The complete database-specific strategies are provided in Appendix 1.

### Eligibility criteria

Literature screening was performed based on the Population, Intervention, Comparison, Outcome, and Study Design (PICOS) framework. Studies were eligible for inclusion if they met the following criteria:

Population (P): Adult patients (≥18 years of age) with a clinical diagnosis of idiopathic Parkinson's disease (PD), established in accordance with the Movement Disorder Society (MDS) clinical diagnostic criteria or the United Kingdom Parkinson's Disease Society Brain Bank (UK PDSBB) criteria (Goldman, 2025). No restrictions were placed on sex or disease duration.

Interventions (I): (1) fecal microbiota transplantation (FMT), (2) probiotic formulations, (3) synbiotic formulations, (4) placebo, and (5) routine care.

Comparisons (C): **Placebo or non-microbial conventional management. To minimize confounding in indirect comparisons, conventional management (designated as ‘Routine Care') was defined across the included trials as the continuation of stable-dose standard anti-Parkinsonian pharmacotherapy, such as levodopa/carbidopa formulations or dopamine receptor agonists. Trials were excluded if the routine care arm introduced active modifications to the baseline medication schedule, or permitted the concomitant use of probiotic or synbiotic products, commercial prebiotics, targeted prebiotic fibers, or structured physical exercise regimens during the trial period**.

Outcomes (O): Trials were required to evaluate at least one of the following primary or secondary clinical endpoints: (1) core motor symptom metrics, specifically the Movement Disorder Society-Unified Parkinson's Disease Rating Scale Part III (MDS-UPDRS III) score; (2) non-motor symptoms or quality-of-life indicators, including spontaneous bowel movement (SBM) frequency, constipation severity, global gastrointestinal symptom scores, and the 39-item Parkinson's Disease Questionnaire (PDQ-39) score.

Study design (S): Peer-reviewed, formally published randomized controlled trials (RCTs).

### Exclusion criteria

We excluded patients with atypical or secondary parkinsonism, severe organic gastrointestinal disease, or recent major gastrointestinal surgery. We also excluded non-randomized studies, animal studies, editorials, case reports, and crossover trials for which first-period data could not be extracted. Studies with unusable outcome data were excluded after attempts to obtain clarification from the investigators.

### Screening process

To manage the retrieved records and remove duplicate entries, we compiled all titles into EndNote citation software. The screening process then followed three progressive stages. First, two reviewers independently evaluated the titles and abstracts of all articles, retaining any record that showed potential relevance. Next, they assessed these selected studies against our predefined criteria, resolving any differing opinions through direct discussion or consultation with a third reviewer. Finally, the team performed a rigorous full-text review of the remaining papers to confirm they met all eligibility standards before final inclusion.

### Data extraction

To collect data uniformly, we developed a standardized extraction framework to record all relevant study features and outcomes. Working independently, two reviewers extracted the data, settling any discrepancies through direct discussion or by consulting a third reviewer. The captured information covered three primary areas: first, study and baseline characteristics, including publication year, design, sample size, and participant demographics (age, sex, disease subtype, duration, and body mass index); second, treatment specifics, detailing the type of therapy, exact bacterial strains, dosage, formulation, duration, and any combined regimens; and finally, control group details, such as placebo or non-treatment protocols. All extracted outcome metrics included specific assessment timepoints, follow-up durations, and clinical symptom scores.

### Outcome measures

To ensure methodological rigor and transparency, all clinical endpoints evaluated in this network meta-analysis were predefined *a priori* and partitioned into primary and secondary outcome measures:

#### Primary outcomes

The primary efficacy endpoints comprised (1) central neurological motor impairment, quantified by the mean change in the Movement Disorder Society-Unified Parkinson's Disease Rating Scale Part III (MDS-UPDRS III) scores from baseline to post-intervention; and (2) peripheral autonomic dysmotility, assessed by the average weekly changes in spontaneous bowel movement (SBM) frequency.

#### Secondary outcomes

The secondary endpoints encompassed exploratory multi-dimensional scales and biomarkers, including the total UPDRS score, global gastrointestinal symptom profiles, psychological metrics (Anxiety and Depression scores), systemic somatic parameters (Sleep and Cognitive Function scores), health-related Quality of Life (PDQ-39) metrics, and biochemical oxidative strain indices, namely Total Antioxidant Capacity (TAC) and Malondialdehyde (MDA) concentrations.

These outcomes were selected to represent complementary aspects of PD. MDS-UPDRS III measures motor impairment, SBM frequency provides a direct measure of constipation, global gastrointestinal scores capture broader gastrointestinal symptoms, and PDQ-39 assesses health-related quality of life.

### Quality assessment of evidence

Two reviewers independently assessed risk of bias using Cochrane RoB 2 and resolved disagreements by consensus. Confidence in the network estimates was assessed with CINeMA across within-study bias, reporting bias, indirectness, imprecision, heterogeneity, and incoherence (Nikolakopoulou et al., 2020; Papakonstantinou et al., 2020). Potential effect modifiers, including administration route, follow-up duration, disease severity, and background medication, were examined descriptively to assess the transitivity assumption.

### Statistical analysis

Network meta-analyses (NMAs) were conducted using Stata 17.0 via the mvmeta command, applying a multivariate random-effects model within a frequentist framework (Rücker, 2012). Two primary endpoints were evaluated: changes in motor function (MDS-UPDRS III scores) and spontaneous bowel movement (SBM) frequency. Additionally, global gastrointestinal symptom relief and health-related quality of life (PDQ-39 scores) were analyzed as secondary endpoints. Treatment effects were expressed as mean differences (MD) or standardized mean differences (SMD) alongside 95% confidence intervals (CIs). To account for the non-independence of multi-arm trials sharing a common control group, within-study correlations across treatment arms were modeled using a multivariate variance-covariance structure based on a design-by-treatment data configuration under the restricted maximum likelihood (REML) approach.

For crossover randomized trials, specific data extraction protocols were implemented to avoid carry-over effects. The duration of the washout periods was reviewed for each crossover cohort. For trials with an adequate washout interval where participants returned to their baseline clinical status prior to the crossover phase, data from both treatment phases were extracted. In instances where the washout period was insufficient or its details were not reported in the primary text, data extraction was limited to the first intervention period, treating the cohort as a parallel-group trial to minimize potential carry-over effects.

A common heterogeneity variance (τ^2^) was assumed across treatment comparisons to support estimation in the sparse network. This modeling choice does not imply clinical homogeneity. Residual heterogeneity was interpreted together with the descriptive comparison of potential effect modifiers and the limitations of the available reporting.

Change-from-baseline values were preferred for continuous outcomes; when these were unavailable, final values were used. When the standard deviation (SD) of a change score was missing and no study-specific correlation was available, it was calculated using the Cochrane variance formula with an assumed baseline-to-follow-up correlation of *R* = 0.5 (Higgins et al., 2023). This value was selected *a priori* as a pragmatic midpoint representing a moderate within-person correlation; it is not presented as a Cochrane-recommended default. The Cochrane Handbook recommends examining plausible alternative correlations when possible. Because the required study-level data were insufficient for a complete correlation sensitivity analysis, uncertainty arising from this assumption is acknowledged in the limitations. Means and SDs were estimated from medians and ranges or interquartile ranges using the methods of Wan et al. and Luo et al. (Wan et al., 2014; Luo et al., 2018).

### Principles for merging intervention nodes

To avoid disconnecting the sparse network, treatment arms were grouped according to their main microbiota-targeted component. The following rules were applied:

FMT node: Oral capsules, colonoscopic administration, and other enteral delivery routes were grouped as FMT because all aimed to transfer donor microbiota. Delivery route and donor preparation may nevertheless modify efficacy, so the pooled FMT estimate should not be interpreted as evidence that all routes are equivalent.

Probiotic and synbiotic nodes: Regimens were assigned according to the primary microbial intervention. Some probiotic trials also included vitamin D, prebiotic fiber, or structured exercise. When these adjuncts were not matched in the comparator arm, the estimated effect may reflect the combined regimen rather than probiotics alone.

These broader nodes were retained because splitting every formulation, route, and co-intervention would have produced disconnected or single-study nodes. The limited number of trials did not support reliable regimen-specific sensitivity analyses. We therefore treat node-level estimates as exploratory and explicitly discuss possible confounding from delivery routes and co-interventions.

The NMA first compared the main treatment categories (placebo, routine care, probiotics, synbiotics, and FMT). Prespecified subgroup analyses examined administration route and follow-up duration. Network plots described the evidence structure, SUCRA values summarized ranking probabilities, and sensitivity analyses excluded studies with high risk of bias or small samples to assess robustness (Bafeta et al., 2014; Higgins et al., 2023).

## Results

### Literature selection

A total of 1,405 records were retrieved from eight databases, including PubMed (*n* = 561), Embase (*n* = 49), the Cochrane Library (*n* = 128), Web of Science (*n* = 620), CNKI (*n* = 10), VIP (*n* = 12), Wanfang (*n* = 10), and CBM (*n* = 15). After removing 353 duplicate entries, 1,052 unique records remained for the initial title and abstract screening. During this first screening phase, a total of 825 reports were excluded: 456 reports were excluded because they were unrelated to the core objective of this study, and an additional 369 records were excluded for not meeting the preliminary inclusion criteria. This screening left 227 articles that underwent a comprehensive full-text eligibility assessment. Of these, 208 papers were subsequently excluded based on the following specific criteria: Not meeting diagnostic criteria (*n* = 42); Non-probiotic interventions or combined treatments where the probiotic effect could not be isolated (*n* = 58); Inadequate or irrelevant outcome data (*n* = 49); Duplicate or overlapping studies (*n* = 33); Full text unavailable or insufficient data for extraction (*n* = 26). Finally, 19 RCTs (**?**Wang et al., 2025; Figura et al., 2026; Du et al., 2025; Liu et al., 2023; Cheng et al., 2023; Wang et al., 2020; Zali et al., 2024; Mehrabani et al., 2023; Ibrahim et al., 2020; Tan et al., 2021; DuPont et al., 2023; Barichella et al., 2016; Ghalandari et al., 2023; Fang et al., 2022; Yang et al., 2025; Scheperjans et al., 2024; Ramadan et al., 2025; Leta et al., 2025) met the inclusion criteria and were included in the qualitative and quantitative synthesis (Figure 1).

**Figure 1:**
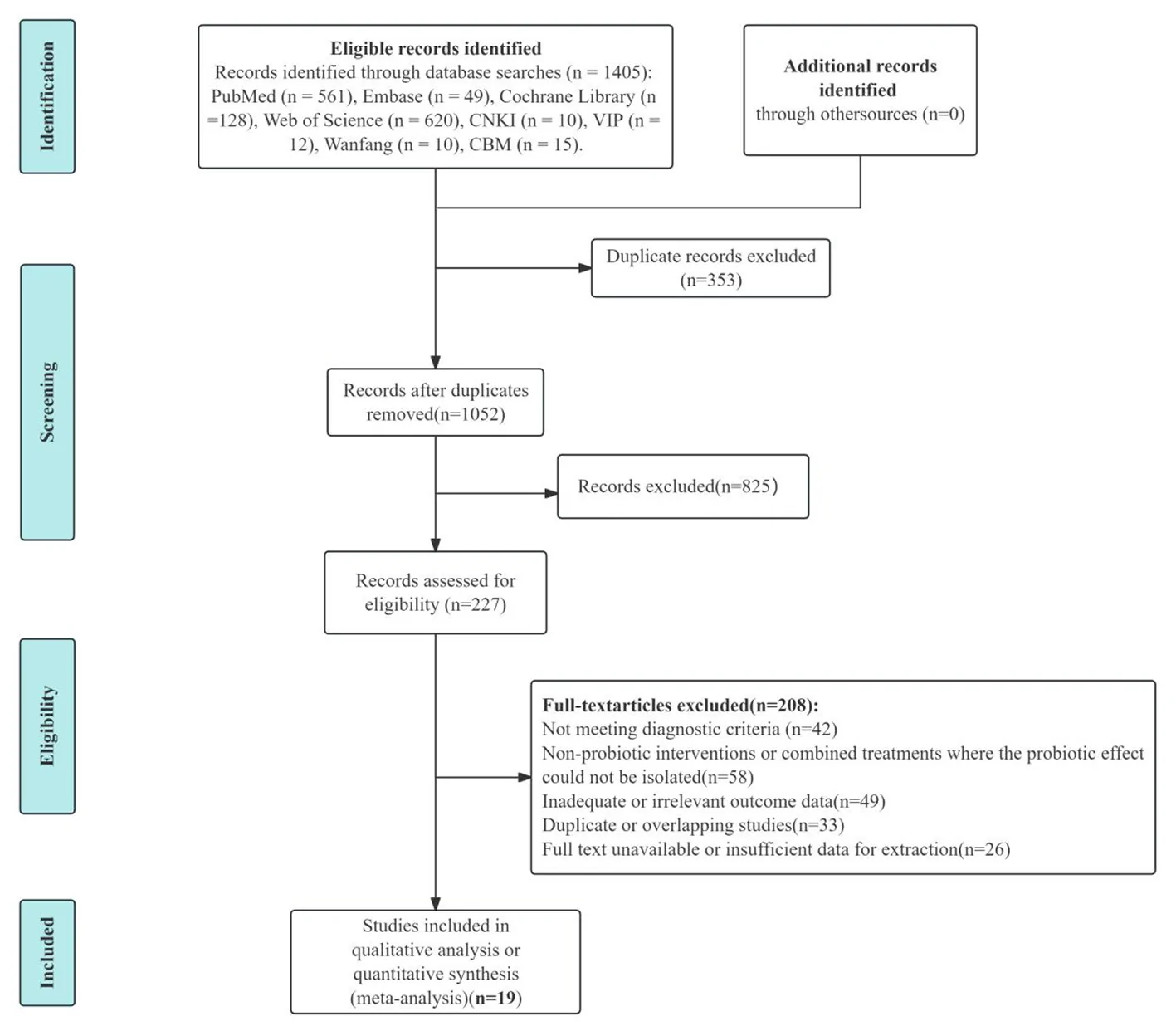
PRISMA flow diagram of study identification and selection process.

### Study characteristics

Ultimately, 19 randomized controlled trials (RCTs) published between 2016 and 2026 met the eligibility criteria, including 1,156 patients: 603 in intervention arms and 553 in control arms. The network included five intervention nodes: FMT, probiotics, synbiotics, routine care, and placebo (Tables 1, 2). The network was connected for quantitative synthesis, but reporting of important effect modifiers was incomplete and comparability across all indirect comparisons could not be confirmed. Outcomes included motor symptoms, gastrointestinal symptoms, SBM frequency, and quality of life.

**Table 1:** Baseline characteristics of included studies.

Study	Trial registration	Country and region	Design	Follow-up duration	Number of participants (T/C)	Randomized treatments (T/C)	Duration (mean ±SD), years	Mean age	Male %	Mean BMI	H-Y
Zhang et al. (2024)	NR	China	RCT	3 weeks	72 (36/36)	dFMT vs. aFMT	2.5 ± 1.2 (dFMT)	64.2 ± 7.8 (dFMT)	54.5%	22.8 ± 2.4	I-III
Wang et al. (2025)	ChiCTR2100050380	China	RCT	Single dose	30 (15/15)	Fresh FMT vs. Placebo	3.0 (median)	68.0 (median)	60%	22.1 ± 2.3	I-III
Figura et al. (2026)	NCT05204641	Poland	RCT	Single dose	59 (28/31)	Mbiotix FMT vs. Placebo	6.5 (median)	64.0 (median)	49.2%	25.5	NR
Du et al. (2025)	ChiCTR2200065638	China	RCT	12 weeks	50 (25/25)	Probiotics vs. Control	5.8 ± 4.3 (Pro)	63.8 ± 8.2 (Pro)	56%	23.4 ± 3.1	I-III
Liu et al. (2023)	NR	China	RCT	4 weeks	51 (25/26)	FMT capsule vs. Placebo	5.3 ± 2.1 (FMT)	65.4 ± 6.8 (FMT)	51.0%	NR	≤ II
Cheng et al. (2023)	ChiCTR1900021405	China	RCT	12 weeks	54 (27/27)	Oral FMT vs. Placebo	6.7 ± 4.0 (FMT)	60.5 ± 8.7 (FMT)	59.3%	22.3 ± 2.2	I-III
Wang et al. (2020)	NR	China	RCT	16 weeks	39 (20/19)	Triple Probiotics vs. Control	6.2 ± 2.4 (Joint)	64.2 ± 7.5 (Joint)	51.3%	NR	NR
Zali et al. (2024)	IRCT20200921048793N1	Iran	RCT	12 weeks	46 (23/23)	Probiotics+VitD vs. Placebo	7.8 ± 4.5 (Pro)	61.2 ± 8.5 (Pro)	60.9%	26.5 ± 3.8	≤ III
Mehrabani et al. (2023)	IRCT20180818040827N2	Iran	RCT	12 weeks	80 (40/40)	Synbiotic vs. Placebo	6.2 ± 4.1 (Syn)	65.8 ± 7.1 (Syn)	57.5%	26.3 ± 3.5	≤ III (29)/≥III (11)
Ibrahim et al. (2020)	NCT04451096	Malaysia	RCT	8 weeks	48 (22/26)	Multi-strain Probiotics vs. Placebo	6.5 ± 4.5 (Pro)	64.0 ± 8.0 (Pro)	43.8%	24.3 ± 4.5	I-IV
Tan et al. (2021)	NR	Malaysia	RCT	4 weeks	72 (34/38)	Multi-strain Probiotics vs. Placebo	5.8 ± 4.8 (Pro)	65.1 ± 8.1 (Pro)	62.5%	24.1 ± 3.9	NR
DuPont et al. (2023)	NR	USA	RCT	12 weeks	12 (8/4)	FMT vs. Placebo	6.0 (FMT)	68.0 (FMT)	50.0%	NR	≤ III
Barichella et al. (2016)	NCT02459717	Italy	RCT	4 weeks	120 (80/40)	Probiotics+Fiber vs. Placebo	6.7 ± 4.8 (Pro)	68.5 ± 7.3 (Pro)	54.2%	24.5 ± 3.5	I-V
Ghalandari et al. (2023)	IRCT20170608034390N11	Iran	RCT	8 weeks	30 (15/15)	Probiotics (Comflor) vs. Placebo	6.5 ± 3.2 (Pro)	62.5 ± 7.5 (Pro)	60.0%	26.8 ± 4.2	I-IV
Fang et al. (2022)	NR	China	RCT	12 weeks	60 (30/30)	Probiotics vs. Placebo	5.5 ± 2.3	64.5 ± 8.2	53.3%	NR	NR
Yang et al. (2025)	NR	China	RCT	24 weeks	154 (77/77)	Probiotics+movement vs. movement	3.2 ± 1.1 (Pro)	62.5 ± 6.8 (Pro)	55.8%	NR	I-III
Scheperjans et al. (2024)	NCT04671030	Finland	RCT	12 weeks	45 (30/15)	FMT vs. Placebo	7.0 (median)	66.0 (median)	55.6%	25.8	≤ II
Ramadan et al. (2025)	NR	Egypt	RCT	12 weeks	66 (33/33)	Synbiotic vs. Placebo	7.1 ± 3.9 (Pro)	56.06 ± 6.2 (Syn)	56.7%	26.1 ± 3.4	I-IV
Leta et al. (2025)	NR	UK	RCT	12 weeks	68 (35/33)	Probiotic vs. Placebo	6.8 ± 4.2 (Pro)	68.17 ± 8.29(Pro)	55.0%	25.9 ± 3.7	II-III

NR: not reported.

**Table 2:** Detailed profile of included trials.

Study	Assigned node	Specific formulation/ product	Detailed microecological composition (strains/materials)	Reported dose	Duration	Administration route
Zhang R (Zhang et al., 2024)	FMT	Donor feces via TET tube	Transendoscopic Enteral Tubing (TET) engraftment	200 units initially, then 50	3 cycles	Endoscopic tube
Wang J (Wang et al., 2025)	FMT	Fresh single-donor stool solution	Screened single-donor fecal microbiota slurry	100g stool/200mL	Single dose	Colonoscopy
Figura (Figura et al., 2026)	FMT	Mbiotix HBI Suspension	High-capacity donor microbiota	60g stool suspension	Single dose	Colonoscopy
Du Y (Du et al., 2025)	Probiotics	*Bacillus licheniformis* + Triple-strain	*B. licheniformis* combined with standard live triple viable bacteria	2.5 × 109 CFU	12 weeks	Oral
Liu J (Liu et al., 2023)	FMT	Fecal microbiota capsule	Standardized frozen donor microbiota powder	4 capsules per dose	6 consecutive days	Oral capsule
Cheng Y (Cheng et al., 2023)	FMT	Donor microbiota capsules	Concentrated donor feces microencapsulated	16 capsules per dose	3 weeks	Oral capsule
Wang SY (Wang et al., 2020)	Probiotics	Live Triple Viable Bacteria	Combined multi-strain live viable capsule formulation	2 tablets per dose	16 weeks	Oral
Zali A (Zali et al., 2024)	Probiotics	Biologics + Vitamin D	*L. acidophilus, L. rhamnosus, L. reuteri, L. paracasei, B. longum, Bacillus coagulans*	2 × 109 CFU	12 weeks	Oral
Mehrabani (Mehrabani et al., 2023)	Synbiotics	5-Strain Probiotics + Inulin	*L. acidophilus* (LAA-5), *L. rhamnosus* (LAR-7), *L. plantarum* (LAP-10), *B. longum* (BIA-8), *S. thermophilus* + 4g Inulin	5 × 109 CFU	12 weeks	Oral suspension
Ibrahim (Ibrahim et al., 2020)	Probiotics	Hexbio + FOS	*L. acidophilus* (BCMC 12130), *L. casei* (BCMC 12313), *L. lactis* (BCMC 12451), *B. infantis* (BCMC 02129), *B. longum* (BCMC 02120) + FOS	30 × 109 CFU	8 weeks	Oral suspension
Tan et al. (2021)	Probiotics	Hexbio Mixture	*L. acidophilus, L. gasseri, L. reuteri, L. rhamnosus, B. bifidum, B. longum, E. faecalis, E. faecium*	10 × 109 CFU	4 weeks	Oral
DuPont et al. (2023)	FMT	Lyophilized donor microbiota capsule	Screened frozen donor fecal powder	60g equivalent	12 weeks	Oral capsule
Barichella et al. (2016)	Probiotics	Multi-strain Probiotics + Prebiotic fiber	*S. thermophilus, E. faecium, L. rhamnosus* GG, *L. acidophilus, L. plantarum, L. paracasei, L. delbrueckii, B. breve, B. lactis*	2.5 × 109 CFU	4 weeks	Oral (milk)
Ghalandari et al. (2023)	Probiotics	Comflor Formulation	*L. plantarum, L. casei, L. acidophilus, L. bulgaricus, B. infantis, B. longum, B. breve, S. thermophilus*	4.5 × 1011 CFU	8 weeks	Oral
Fang et al. (2022)	Probiotics	Live Triple Viable Capsules	*Bifidobacterium, Lactobacillus, Enterococcus* complex	0.84g/day	12 weeks	Oral
Yang et al. (2025)	Probiotics	Triple Viable Bacteria + Exercise	Live multi-strain viable formulation	420mg per dose	12 weeks	Oral
Scheperjans et al. (2024)	FMT	Anaerobically prepared donor stool	Screened healthy donor microbial community	30g stool + glycerol	Single dose	Colonoscopy
Ramadan et al. (2025)	Synbiotics	Livia Formulation	*Lactobacillus acidophilus* + Inulin matrix	10 × 109 CFU	12 weeks	Oral suspension
Leta et al. (2025)	Probiotics	Symprove Liquid	*L. acidophilus* (NCIMB 30157), *L. plantarum* (NCIMB 30173), *L. rhamnosus* (NCIMB 30174), *E. faecium* (NCIMB 30176)	4.08 × 108 CFU/mL	12 weeks	Oral

### Risk of bias, certainty of evidence, and consistency

Evaluated across five distinct domains using the Cochrane Risk of Bias 2.0 tool, 14 trials were classified as having a low risk of bias, while the remaining 5 were graded with some concerns **(Figure 2 and Appendix 3)**. Although 15 out of the 19 trials explicitly stated that a randomization process was used, they lacked sufficient detail regarding sequence generation and allocation concealment. Regarding deviations from intended interventions and outcome measurement biases, approximately half of the included studies provided detailed blinding protocols for both participants and outcome assessors, whereas several single-center or open-label trials offered limited descriptors. Missing outcome data was low, as the majority of trials reported complete follow-up statistics. A small number of studies that failed to provide full statistical summaries or preset outcomes were noted for potential selective reporting.

**Figure 2:**
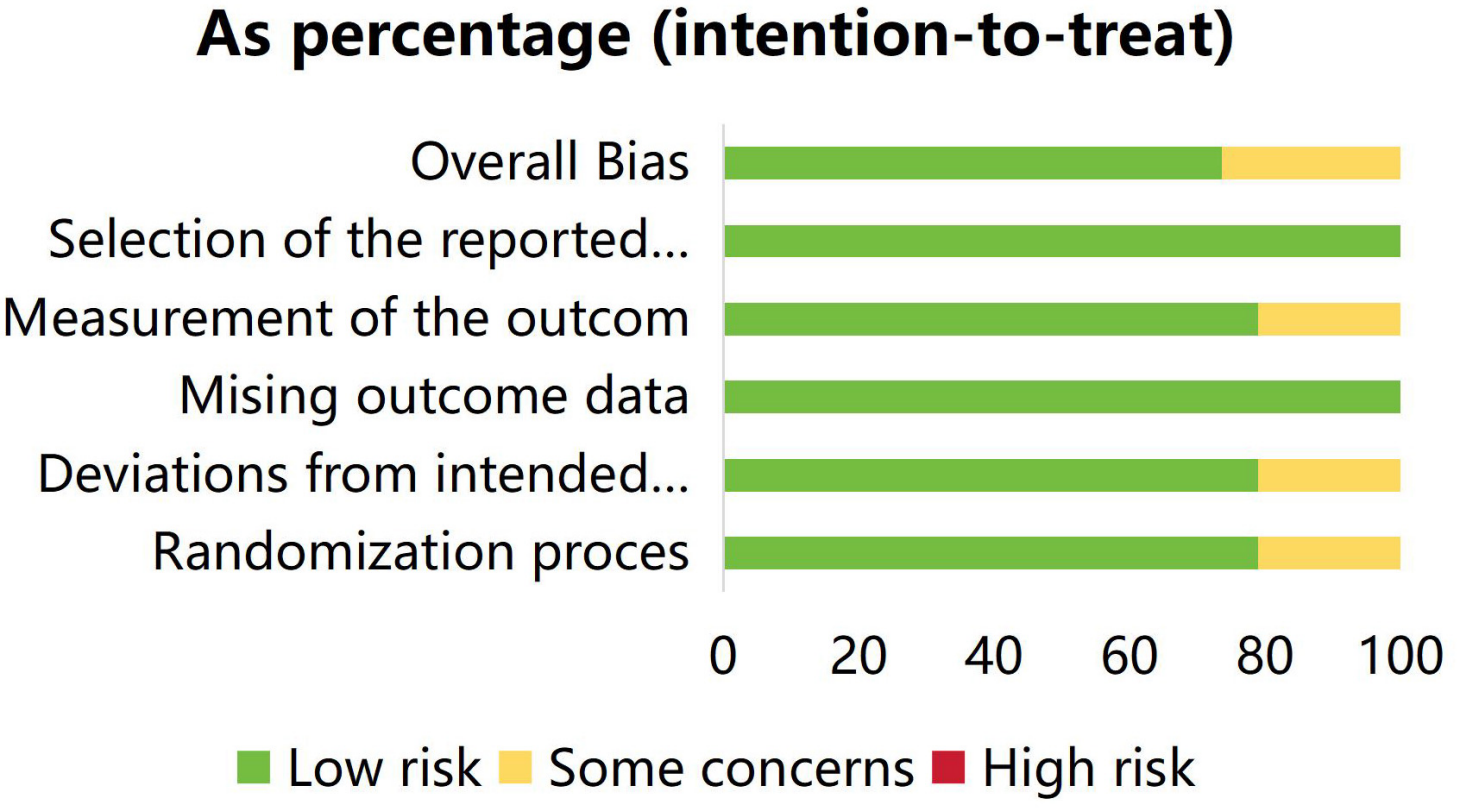
Risk of bias assessment of included studies.

Confidence in the network estimates was assessed using CINeMA. Summary assessments for the primary outcomes are presented in Table 3, with full details in **Appendix 14**. Because the networks were predominantly star-shaped and contained no closed loops, local inconsistency could not be estimated using node-splitting. We therefore examined potential effect modifiers descriptively, including age, disease duration, baseline severity, background medication, and follow-up duration (Table 4). Reporting was incomplete, so clinical homogeneity and transitivity across all comparisons could not be confirmed.

**Table 3:** CINeMA confidence ratings for key pairwise comparisons across primary endpoints.

Comparison pair	Outcome	Within-study bias	Reporting bias	Indirectness	Imprecision	Heterogeneity	Incoherence	Overall Certainty
FMT vs. Placebo	MDS-UPDRS III	Major concerns	Some concerns	No concerns	Major concerns	Some concerns	No concerns	Very Low
Synbiotics vs. Placebo	MDS-UPDRS III	Some concerns	Some concerns	No concerns	Major concerns	Some concerns	No concerns	Low
Probiotics vs. Placebo	MDS-UPDRS III	Some concerns	No concerns	No concerns	Major concerns	No concerns	No concerns	Low
Probiotics vs. Placebo	SBM Frequency	Some concerns	No concerns	No concerns	No concerns	Some concerns	No concerns	Moderate
Synbiotics vs. Placebo	SBM Frequency	Some concerns	Some concerns	No concerns	Some concerns	Some concerns	No concerns	Low
FMT vs. Placebo	SBM Frequency	Major concerns	Some concerns	No concerns	Major concerns	Major concerns	No concerns	Very Low
Probiotics vs. Placebo	Gastrointestinal Symptom	No concerns	Low risk	No concerns	Major concerns	No concerns	No concerns	Low
FMT vs. Placebo	Gastrointestinal Symptom	No concerns	Low risk	No concerns	Major concerns	No concerns	No concerns	Very Low
Placebo vs. Routine Care	Gastrointestinal Symptom	No concerns	Low risk	No concerns	Major concerns	No concerns	No concerns	Very Low

**Table 4:** Baseline demographics and clinical potential effect modifiers across operational intervention nodes.

Baseline characteristics (potential effect modifiers)	FMT node (*n* = 7 trials overall; *n* = 5 in network meta-analysis)	Probiotics node (*n* = 10 studies)	Synbiotics node	Placebo/control node	Across-group homogeneity
No. of participants (T/C)	169	361	73	553	/
Mean age (years, range of means)	60.5−68.0	56.1−68.5	56.1−65.8	56.1−68.5	Not statistically assessed
Male percentage (%)	49.2%−60.0%	43.8%−62.5%	56.7%−57.5%	43.8%−62.5%	Not statistically assessed
PD duration (years, range of means)	2.5−7.0	3.2−7.8	6.2−7.1	2.5−7.8	Not statistically assessed
Mean BMI (kg/m2, range of means)	22.1−25.8	23.4−26.8	26.1−26.3	22.1−26.8	Not statistically assessed
Baseline MDS-UPDRS III score (mean ± SD)	31.4 ± 8.5	32.9 ± 9.1	30.8 ± 7.6	32.1 ± 8.8	Not statistically assessed
Geographic distribution	China, Poland, USA	China, Iran, Malaysia, Italy, UK	Iran, Egypt	Global	Varied across nodes

The common heterogeneity variances (τ^2^) ranged from 0.07 to 0.08 across outcomes (**Appendix 4**, **Supplementary Table 4.1**). As summarized in Table 3, confidence in estimates for MDS-UPDRS III ranged from Low to Very Low, mainly because of imprecision and within-study bias.

Confidence in the comparison of oral probiotics with placebo for SBM frequency was Moderate (Table 3; **Appendix 14**, **Supplementary Table 14.2**). Comparison-adjusted funnel plots were examined as exploratory assessments of asymmetry (**Appendix 8**), but several outcomes included fewer than 10 trials; therefore, publication bias could not be reliably confirmed or excluded.

### Primary network analysis

#### Motor outcome: MDS-UPDRS III score

For the motor outcome evaluated by MDS-UPDRS III scores, the network meta-analysis incorporated 15 studies enrolling 957 participants (503 in the intervention groups and 454 in the control groups) across 4 active intervention nodes (Synbiotics, FMT, Probiotics, and Routine Care) plus the reference node Placebo. Placebo served as the central hub of the network, with the vast majority of evidence derived from direct comparisons against Placebo; no direct head-to-head comparisons were identified between most active interventions, and the network adopted a star-like topology without closed loops (Figure 3A).

**Figure 3:**
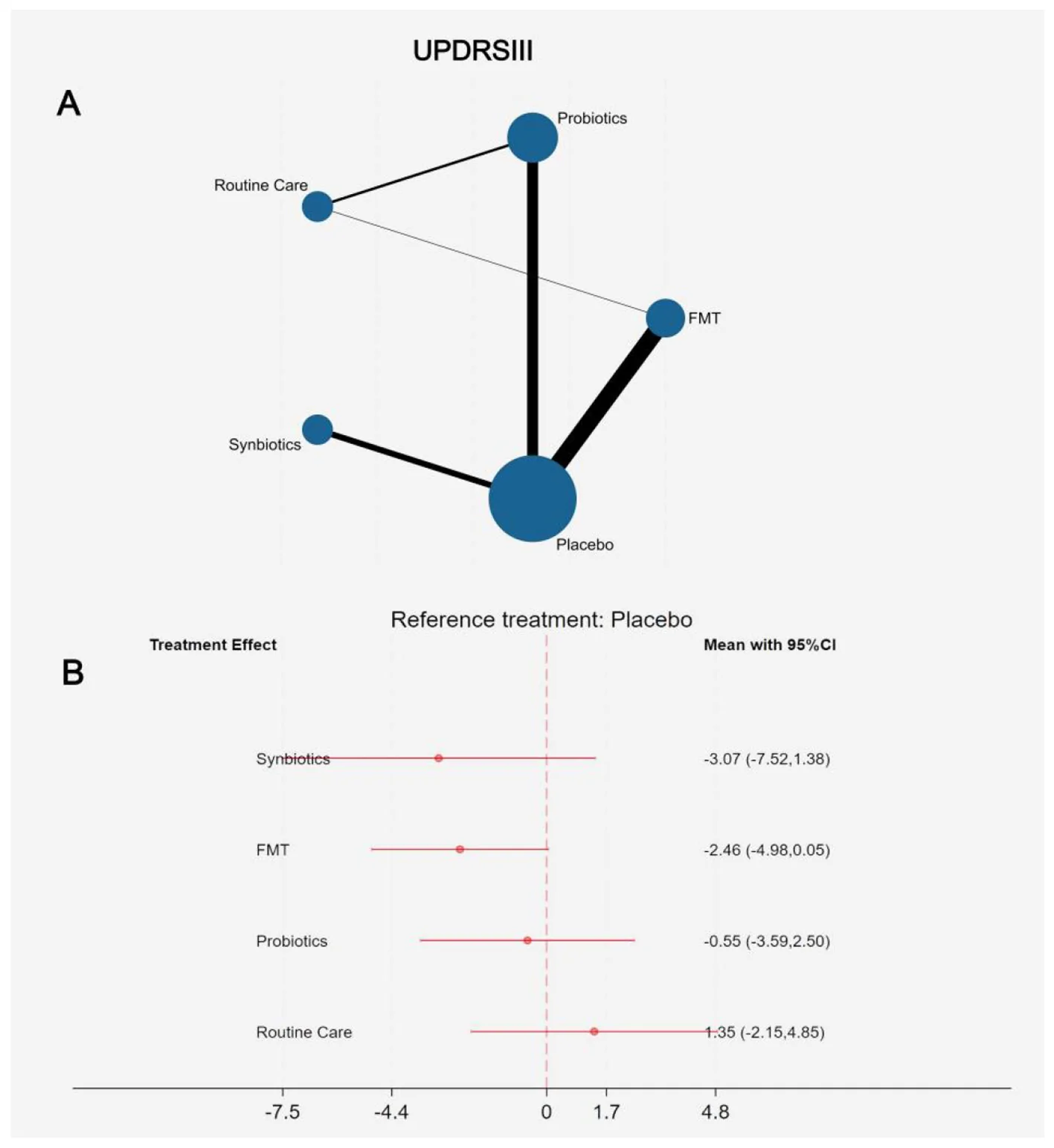
Network plot and forest plot for MDS-UPDRS III. **(A)** Evidence network: A loopless, star-shaped network of five treatment nodes. Node sizes correspond to sample sizes (Placebo is the largest). Line thickness represents the number of direct comparisons, which are concentrated between the active interventions and Placebo. **(B)** Forest plot: Mean differences (MDs) with 95% confidence intervals (CIs) compared with Placebo. No active intervention achieved statistical significance (all intervals cross the null line, MD = 0): Synbiotics (MD = −3.07, 95% CI: −7.52 to 1.38), FMT (MD = −2.46, 95% CI: −4.98 to 0.05), Probiotics (MD = −0.55, 95% CI: −3.59 to 2.50), and Routine Care (MD = 1.35, 95% CI: −2.15 to 4.85).

Compared with Placebo, none of the active interventions achieved statistically significant reductions in MDS-UPDRS III scores (Figure 3B). In terms of relative ranking probability, Synbiotics ranked highest within the SUCRA distribution (MD = −3.07, 95% CI: −7.52 to 1.38, SUCRA = 82.3%), followed by FMT (MD = −2.46, 95% CI: −4.98 to 0.05, SUCRA = 80.0%) and Probiotics (MD = −0.55, 95% CI: −3.59 to 2.50, SUCRA = 47.4%) **(****Appendix 6**, **Supplementary Table 6.2****)**.

In pairwise comparisons from the league table, no contrasts between any active interventions reached formal statistical significance. Specifically, the comparative estimates for multi-species microbial therapies vs. conventional control were MD = −3.81 (95% CI: −7.72 to 0.09) for FMT vs. Routine Care and MD = 4.42 (95% CI: −1.29 to 10.13) for Routine Care vs. Synbiotics. Between the active microbial formulations, the relative estimates were MD = −1.92 (95% CI: −5.46 to 1.62) for FMT vs. Probiotics and MD = −1.90 (95% CI: −4.26 to 0.47) for Probiotics vs. Routine Care (**Appendix 7**, **Supplementary Table 7.2**). The global network meta-analysis model for neuro-motor function recovery (MDS-UPDRS III) presented a common heterogeneity variance of τ^2^ = 0.08.

#### Gastrointestinal non-motor outcome: spontaneous bowel movements (SBM)

For the non-motor outcome of spontaneous bowel movements (SBM), the network meta-analysis incorporated 7 studies enrolling 399 participants (224 in the intervention groups and 175 in the control groups) across 4 active intervention nodes (Probiotics, Synbiotics, FMT, and Routine Care) plus the reference node Placebo. Placebo served as the central hub of the network, with the vast majority of evidence derived from direct comparisons against Placebo; no direct head-to-head comparisons were identified between most active interventions, and the network adopted a star-like topology without closed loops (Figure 4A).

**Figure 4:**
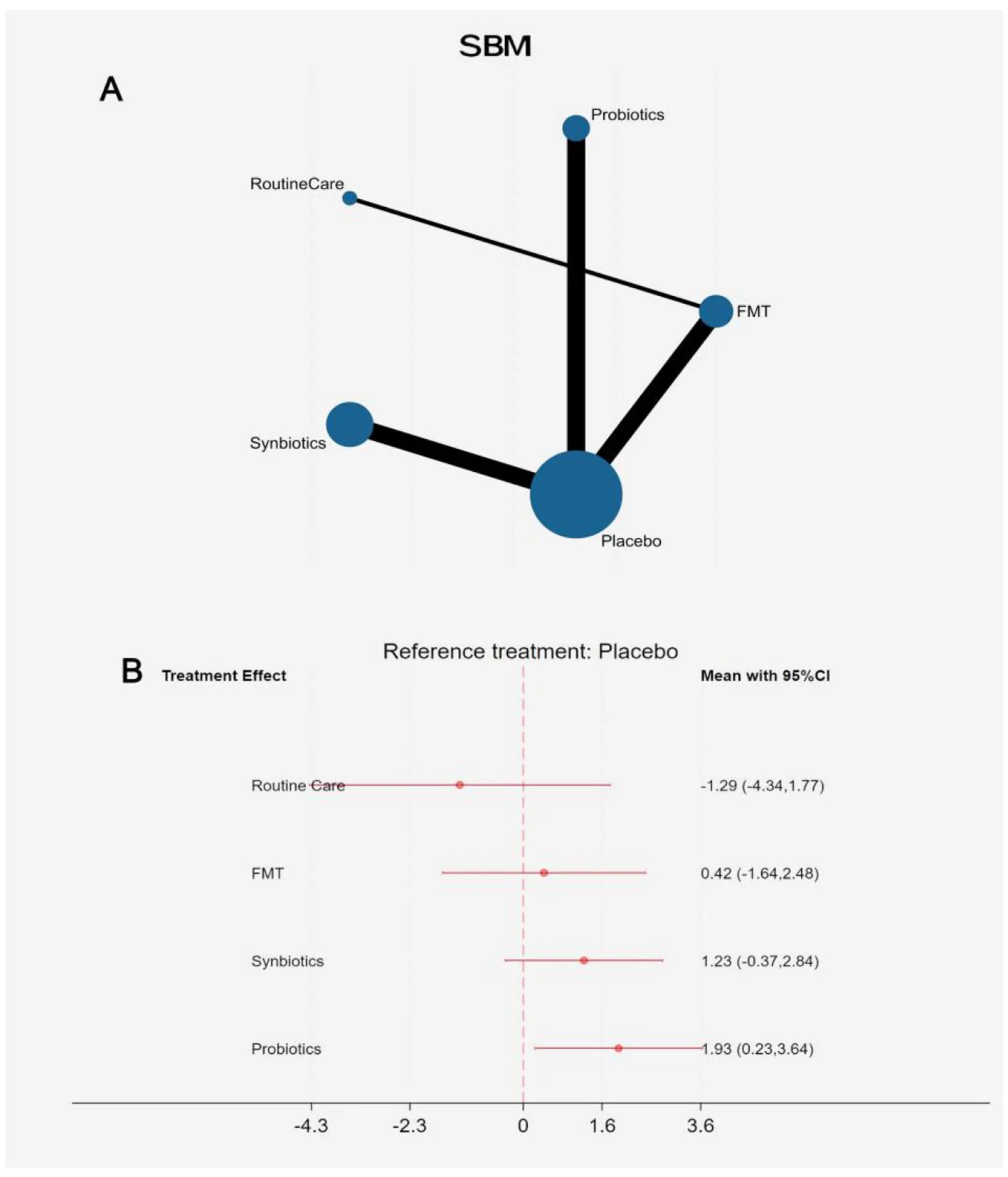
Network plot and forest plot for SBM frequency. **(A)** Evidence network: A loopless, star-shaped network of five treatment nodes. Node sizes correspond to sample sizes (Placebo is the largest). Line thickness represents the number of direct comparisons, which are concentrated between the active interventions and Placebo, with only one direct link existing between FMT and Routine Care. **(B)** Forest plot: Mean differences (MDs) with 95% confidence intervals (CIs) compared with Placebo. Only Probiotics achieved statistical significance (as its interval does not cross the null line, MD = 0). The estimates are: Probiotics (MD = 1.93, 95% CI: 0.23 to 3.64), Synbiotics (MD = 1.23, 95% CI: −0.37 to 2.84), FMT (MD = 0.42, 95% CI: −1.64 to 2.48), and Routine Care (MD = −1.29, 95% CI:−4.34 to 1.77).

Compared with Placebo, Probiotics was the only active intervention that achieved a statistically significant increase in SBM scores (MD = 1.93, 95% CI: 0.23 to 3.64) (Figure 4B). In terms of relative ranking probability, Probiotics ranked highest within the SUCRA distribution (SUCRA = 88.3%), followed by Synbiotics (MD = 1.23, 95% CI: −0.37 to 2.84, SUCRA = 71.1%) and FMT (MD = 0.42, 95% CI: −1.64 to 2.48, SUCRA = 50.4%) **(****Appendix 6**, **supplementary Table 6.3****)**.

In pairwise comparisons from the league table, no contrasts between any other active interventions reached formal statistical significance. Specifically, the comparative estimates for the primary gut microbiota-targeted therapies vs. conventional care were MD = 3.22 (95% CI: −0.28 to 6.73) for Probiotics vs. Routine Care, MD = 1.71 (95% CI: −0.55 to 3.97) for FMT vs. Routine Care, and MD = −2.52 (95% CI: −5.98 to 0.93) for Routine Care vs. Synbiotics. Between the active microbial therapies, the relative estimate was MD = −1.51 (95% CI: −4.20 to 1.17) for FMT vs. Probiotics (**Appendix 7**, **Supplementary Table 7.3**). The frequentist network model evaluating SBM frequency (SBM frequency) demonstrated a common heterogeneity variance of τ^2^ = 0.08.

#### Non-motor outcome: gastrointestinal symptom scores

For the non-motor outcome of gastrointestinal symptoms, the network meta-analysis incorporated 5 studies enrolling 270 participants (132 in the intervention groups and 138 in the control groups) across 3 active intervention nodes (Probiotics, FMT, and Routine Care) plus the reference node Placebo. Placebo served as the central hub of the network, with the direct comparison between Probiotics and Placebo forming the most robust link; no direct head-to-head comparisons were identified between active interventions, and the network adopted a star-shaped topology without closed loops (Figure 5A).

**Figure 5:**
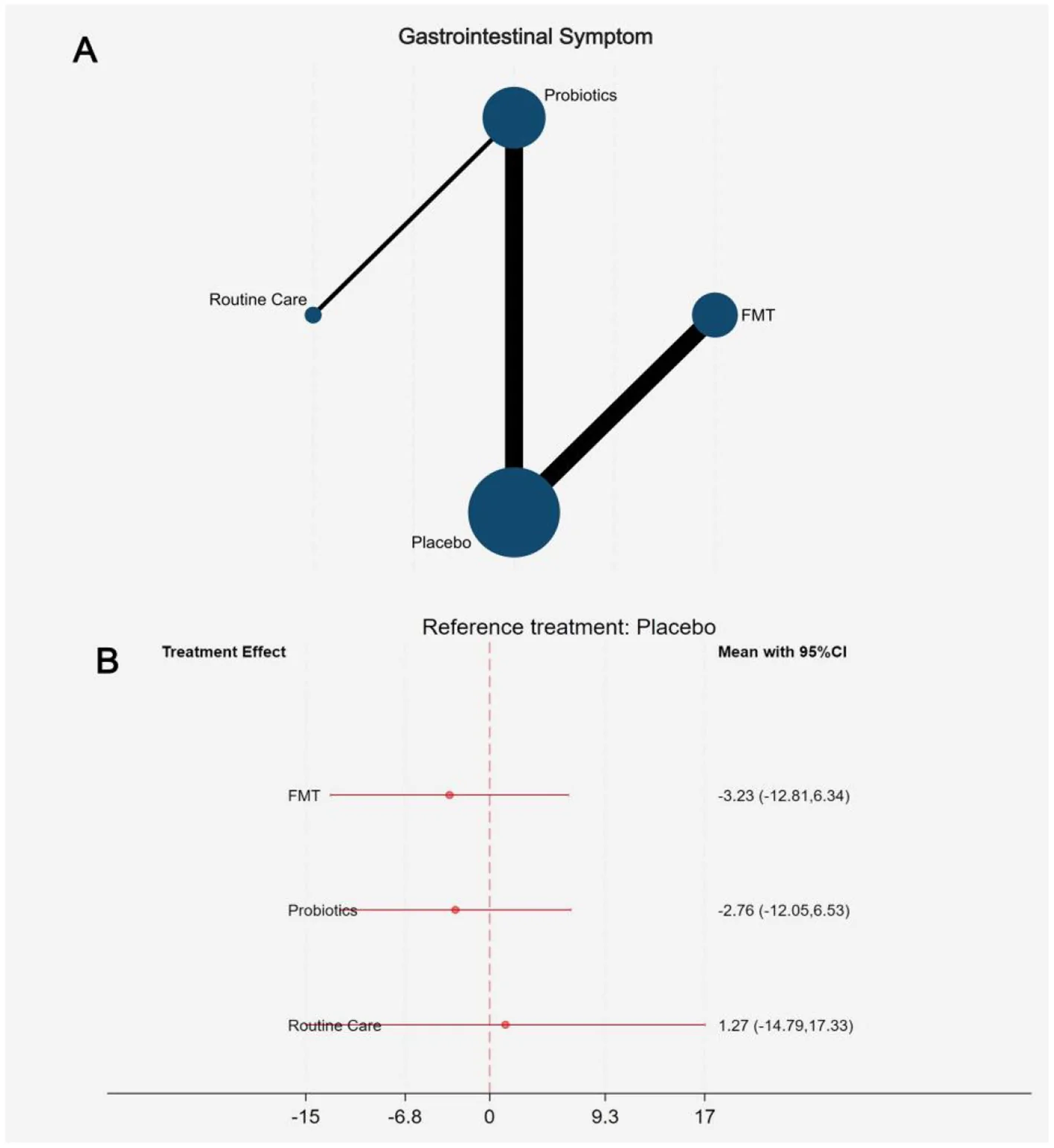
Network plot and forest plot for gastrointestinal symptoms. **(A)** Evidence network: A loopless, star-shaped network of four treatment nodes. Node sizes correspond to sample sizes (Placebo is the largest). Line thickness represents the number of direct comparisons, which are concentrated between the active interventions and Placebo, with only one direct link existing between Probiotics and Routine Care. **(B)** Forest plot: Mean differences (MDs) with 95% confidence intervals (CIs) compared with Placebo. No active intervention achieved statistical significance (all intervals cross the null line, MD = 0). The estimates are: FMT (MD = −3.23, 95% CI: −12.81 to 6.34), Probiotics (MD = −2.76, 95% CI: −12.05 to 6.53), and Routine Care (MD = 1.27, 95% CI: −14.79 to 17.33).

Compared with Placebo, none of the active interventions demonstrated statistically significant reductions in symptom scores (Figure 5B). In terms of relative ranking probability, FMT (MD = −3.23, 95% CI: −12.81 to 6.34, SUCRA = 64.2%) and Probiotics (MD = −2.76, 95% CI: −12.05 to 6.53, SUCRA = 64.2%) shared identical SUCRA values, followed by Routine Care (MD = 1.27, 95% CI: −14.79 to 17.33). Pairwise comparisons among active interventions yielded mean differences of −0.52 (95% CI: −13.95 to 12.91) for FMT vs. Probiotics, −4.57 (95% CI: −23.41 to 14.27) for FMT vs. Routine Care, and −4.05 (95% CI: −17.29 to 9.20) for Probiotics vs. Routine Care, with all confidence intervals overlapping the null value **(****Appendix 6**, **Supplementary Table 6.4****)**.

Pairwise comparisons between active interventions showed no statistically significant differences, as all confidence intervals overlapped the null value: FMT vs. Probiotics (MD = −0.52, 95% CI: −13.95 to 12.91), FMT vs. Routine Care (MD = −4.57, 95% CI: −23.41 to 14.27), and Probiotics vs. Routine Care (MD = −4.05, 95% CI: −17.29 to 9.20) (**Appendix 7**, **Supplementary Table 7.4**). For the global gastrointestinal symptom network, the estimated common heterogeneity variance was τ^2^ = 0.08.

### Health-related quality of life outcome: quality of life score

For quality of life measured with PDQ-39, the network meta-analysis incorporated 9 studies enrolling 489 participants (249 in the intervention groups and 240 in the control groups) across 4 active intervention nodes (Probiotics, Synbiotics, FMT, and Routine Care). Placebo served as the central hub of the network, with the direct comparison between FMT and Placebo forming the most robust link; no direct head-to-head comparisons were identified between active therapies, and the network adopted a star-shaped topology without closed loops (Figure 6A).

**Figure 6:**
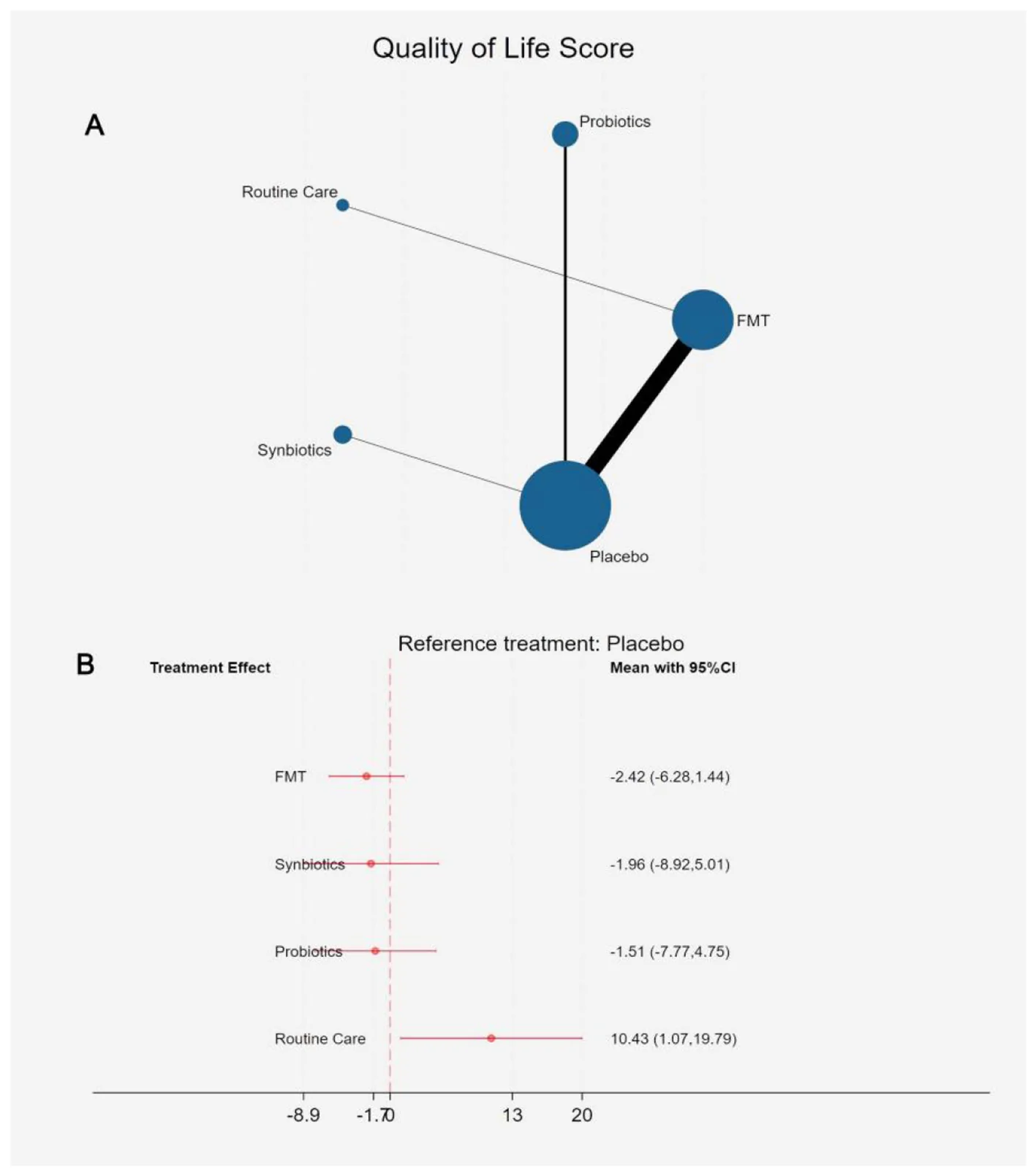
Network plot and forest plot for PDQ-39. **(A)** Evidence network: A loopless, star-shaped network of five treatment nodes. Node sizes correspond to sample sizes (Placebo is the largest). Line thickness represents the number of direct comparisons, which are concentrated between the active interventions and Placebo, with only one direct link existing between FMT and Routine Care. **(B)** Forest plot: Mean differences (MDs) with 95% confidence intervals (CIs) compared with Placebo. Only Routine Care showed a statistically significant difference compared with Placebo, as its interval does not cross the null line (MD = 0). The estimates are: FMT (MD = −2.42, 95% CI: −6.28 to 1.44), Synbiotics (MD = −1.96, 95% CI: −8.92 to 5.01), Probiotics (MD = −1.51, 95% CI: −7.77 to 4.75), and Routine Care (MD = 10.43, 95% CI: 1.07 to 19.79).

Compared with Placebo, none of the active gut microbiota-targeted interventions demonstrated statistically significant differences in PDQ-39 scores, as their 95% confidence intervals overlapped the null value (Figure 6B). Specifically, the estimates relative to Placebo were: FMT (MD = −2.42, 95% CI: −6.28 to 1.44, SUCRA = 75.8%), Synbiotics (MD = −1.96, 95% CI: −8.92 to 5.01, SUCRA = 67.1%), and Probiotics (MD = −1.51, 95% CI: −7.77 to 4.75, SUCRA = 62.9%). Conversely, Routine Care was associated with a statistically significant increase in PDQ-39 scores compared with Placebo (MD = 10.43, 95% CI: 1.07 to 19.79, SUCRA = 1.3%) (**Appendix 6**, **Supplementary Table 6.7**).

In pairwise comparisons, FMT, synbiotics, and probiotics were associated with statistically significant reductions in PDQ-39 scores compared with Routine Care: FMT (MD = −12.85, 95% CI: −21.38 to −4.32), synbiotics (MD = −12.39, 95% CI: −24.06 to −0.73), and probiotics (MD = −11.94, 95% CI: −23.22 to −0.67). No other comparisons showed statistically significant differences (**Appendix 7**, **Supplementary Table 7.7**). For the PDQ-39 network, the estimated common heterogeneity variance was τ^2^ = 0.08.

### Safety and adverse events

Safety reporting was incomplete across the included trials. Reported adverse events were mainly mild and transient, and no treatment-related serious adverse events were reported in the available data; however, comparative and long-term safety could not be established (**Appendix 16**, **Supplementary Table 16.1**).

### Sensitivity analyses

The first sensitivity analysis excluded five studies with high risk of bias or open-label designs (Wang et al., 2025; Liu et al., 2023; Wang et al., 2020; Ghalandari et al., 2023; **?**)). In this restricted analysis, FMT reduced MDS-UPDRS III scores compared with placebo (MD = −3.04, 95% CI: −6.04 to −0.04; **Appendix 12**, **Supplementary Figure 12.1**), whereas the primary analysis was not statistically significant. This change indicates that the motor result is sensitive to study selection and should be considered exploratory. Findings for gastrointestinal symptom scores and SBM frequency were broadly unchanged (Appendix 12, Supplementary Figures 12.2, 12.3).

A second sensitivity analysis restricted the dataset to larger trials. Probiotics (MD = 2.81, 95% CI: 2.30 to 3.32) and synbiotics (MD = 1.21, 95% CI: 0.78 to 1.65) continued to increase SBM frequency compared with placebo (Appendix 13, Supplementary Figure 13.2). Rankings for motor and gastrointestinal symptom outcomes were similar to the primary analysis (**Appendix 13**, **Supplementary Figures 13.1**, **S13.3**).

### Subgroup analysis

Subgroup analyses were planned by administration route. Too few endoscopic-delivery trials were available to form a connected network, so a network meta-analysis could not be estimated for this subgroup. Quantitative subgroup results are therefore reported only for oral administration.

### Motor function recovery (MDS-UPDRS III)

For motor function recovery within the Oral administration subgroup, the network meta-analysis incorporated active microbial intervention nodes and Placebo (Figure 7A). Compared with Placebo, none of the active interventions achieved a statistically significant reduction in MDS-UPDRS III scores, as all 95% confidence intervals crossed the line of no effect (Figure 7B). Based on the SUCRA ranking probabilities, the values were 83.8% for FMT (MD = −3.47, 95% CI: −7.95 to 1.01), 76.7% for synbiotics (MD = −2.99, 95% CI: −7.31 to 1.33), and 48.1% for probiotics (MD = −0.75, 95% CI: −3.73 to 2.24) **(****Appendix 6**, **Supplementary Table 9.1A****)**. Pairwise comparisons from the league table showed no statistically significant differences among any of the active treatment arms **(****Appendix 9**, **Supplementary Table 9.1B****)**.

**Figure 7:**
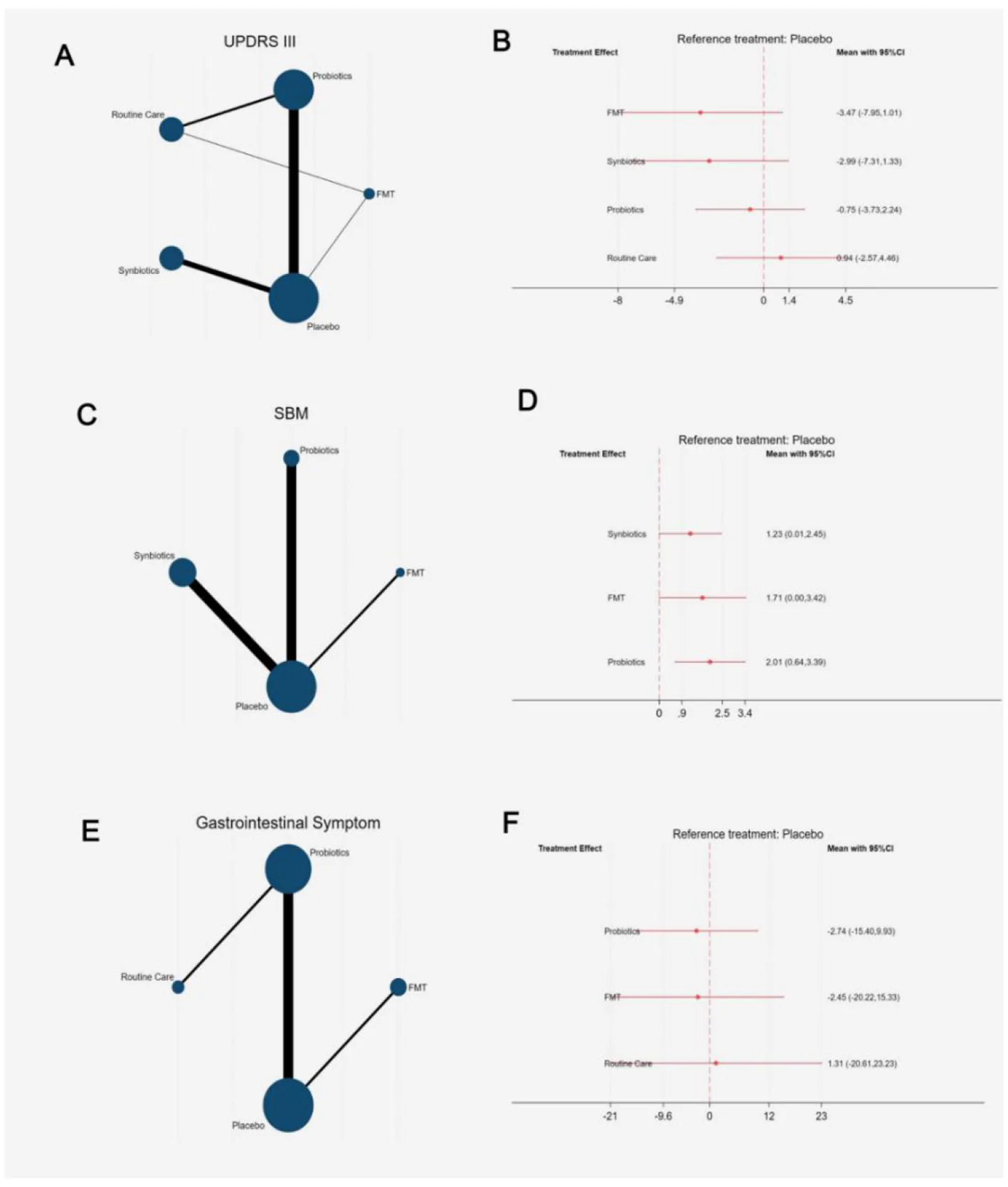
Network plots and forest plots for the oral-administration subgroup. **(A, C, E)** Evidence networks: Star-shaped, loopless network topologies restricted to oral delivery routes for **(A)** MDS-UPDRS III, **(C)** SBM, and **(E)** gastrointestinal symptoms. Node sizes correspond to sample sizes (Placebo is the largest in all networks). Line thickness represents the number of direct comparisons, which are primarily concentrated between the active interventions and Placebo. **(B, D, F)** Forest plots: Mean differences (MDs) with 95% confidence intervals (CIs) compared with Placebo. MDS-UPDRS III **(B)**: No oral intervention achieved statistical significance (all intervals cross the null line, MD = 0): FMT (MD = −3.47, 95% CI: −7.95 to 1.01), Synbiotics (MD = −2.99, 95% CI: −7.31 to 1.33), Probiotics (MD = −0.75, 95% CI: −3.73 to 2.24), and Routine Care (MD = 0.94, 95% CI: −2.57 to 4.46). SBM **(D):** Oral probiotics (MD = 2.01, 95% CI: 0.64 to 3.39) and synbiotics (MD = 1.23, 95% CI: 0.01 to 2.45) increased SBM frequency compared with placebo. Oral FMT showed a numerical increase, but its confidence interval reached the line of no effect (MD = 1.71, 95% CI: 0.00 to 3.42). Gastrointestinal Symptom **(F)**: No oral intervention achieved statistical significance (all intervals cross the null line, MD = 0): Probiotics (MD = −2.74, 95% CI: −15.40 to 9.93), FMT (MD = −2.45, 95% CI: −20.22 to 15.33), and Routine Care (MD = 1.31, 95% CI: −20.61 to 23.23).

### Spontaneous bowel movement (SBM)

In the oral-administration subgroup, probiotics (MD = 2.01, 95% CI: 0.64 to 3.39) and synbiotics (MD = 1.23, 95% CI: 0.01 to 2.45) increased SBM frequency compared with placebo (Figures 7C, D). Oral FMT showed a numerical increase, but its confidence interval reached the line of no effect (MD = 1.71, 95% CI: 0.00 to 3.42). Probiotics had the highest SUCRA value (80.2%), followed by FMT (68.0%) and synbiotics (50.2%) (**Appendix 9**, **Supplementary Tables 9.2A**, **9.2B**).

### Gastrointestinal symptom improvement

For global gastrointestinal symptom mitigation, the network adopted a star-shaped topology without closed loops, dominated by the direct link between Probiotics and Placebo (Figure 7E). Compared with Placebo, no active microbial therapies achieved statistically significant superiority (Figure 7F). While probiotics (MD = −2.74, 95% CI: −15.40 to 9.93) and FMT (MD = −2.45, 95% CI: −20.22 to 15.33) displayed numerical tendencies toward symptom reduction, all 95% confidence intervals spanned the line of no effect. SUCRA calculations revealed parallel potential trends between the two leading interventions, with probiotics scoring 62.0% followed closely by FMT at 56.3%, while routine care performed poorest (SUCRA = 39.1%) (**Appendix 9**, Supplementary Table 9.3A). Pairwise matrix cross-comparisons confirmed no formal statistical differentiation among any active arms (**Appendix 9**, Supplementary Table S9.3B).

Subgroup analyses were also planned by follow-up duration. A medium-to-long-term network could be estimated for MDS-UPDRS III, but no comparison was statistically significant (**Appendix 11**). Extended follow-up data were insufficient to estimate connected networks for the other outcomes.

## Discussion

### Primary findings

This network meta-analysis found that treatment effects differed by outcome. Probiotics increased SBM frequency compared with placebo. No active treatment significantly improved MDS-UPDRS III or PDQ-39 scores compared with placebo in the primary analyses. Higher SUCRA rankings for synbiotics and FMT therefore indicate ranking probabilities, not confirmed superiority.

The certainty of evidence also differed by outcome. The evidence for oral probiotics on SBM frequency was Moderate, whereas evidence for motor function and quality of life was Low to Very Low because of imprecision and risk of bias. The motor effect observed for FMT after excluding higher-risk studies should therefore be regarded as a preliminary sensitivity-analysis signal.

Safety reporting was incomplete. Reported events were mainly mild and transient, but the available data do not establish comparative or long-term safety.

Key supplementary analyses support the same cautious interpretation: the FMT motor estimate changed after exclusion of higher-risk studies, the SBM finding remained stable in larger trials, and oral-route subgroup results favored probiotics and synbiotics for SBM frequency. The medium-to-long-term MDS-UPDRS III subgroup showed no statistically significant differences, and extended follow-up data were insufficient for the other outcomes.

### Comparison with previous research and efficacy robustness

Previous pairwise meta-analyses of probiotics reported improvements in constipation outcomes but inconsistent effects on motor symptoms (Xie et al., 2023; Fang et al., 2023). A recent systematic review of FMT similarly described possible gastrointestinal and motor benefits, while emphasizing small, heterogeneous studies and limited long-term evidence (Nabil et al., 2025). To our knowledge, previous reviews did not compare probiotics, synbiotics, and FMT within the same network. Our NMA provides this comparison, but most contrasts are indirect and the motor evidence remains Low to Very Low certainty.

The primary motor analysis found no statistically significant benefit over placebo. After studies with high risk of bias or open-label designs were excluded, FMT showed a small reduction in MDS-UPDRS III scores (MD = −3.04, 95% CI: −6.04 to −0.04). Because significance depended on study selection, this finding requires confirmation in larger blinded trials.

For MDS-UPDRS III, an improvement of 3.25 points has been proposed as a minimal clinically important within-patient change (Horváth et al., 2015). No intervention reached this threshold in the primary analysis; the FMT estimate in the sensitivity analysis approached it. For SBM frequency, we did not identify a validated PD-specific between-group MCID. The increase with oral probiotics (MD = 1.93, 95% CI: 0.23 to 3.64) may be clinically relevant, but the wide interval and Moderate certainty should be considered.

### Possible mechanisms along the microbiota-gut-brain axis

Several mechanisms may explain the observed gastrointestinal effects, although the clinical trials did not directly test them. Probiotics and synbiotics may alter short-chain fatty acid production, epithelial barrier function, and inflammatory signaling, which could influence bowel motility and the microbiota-gut-brain axis (Duan et al., 2024; Cryan et al., 2019). *Enterococcus faecalis* can metabolize levodopa in the intestine, providing a possible link between microbial composition and drug availability (van Kessel et al., 2019). FMT may produce broader changes in microbial communities than individual probiotic strains, but evidence for effects on neuroinflammation and motor function is largely preclinical (Sampson et al., 2016; Haifer et al., 2022). These mechanisms should therefore be regarded as hypotheses rather than proof of clinical causation.

### Clinical implications

Gut microbiota-targeted therapies should be considered investigational adjuncts rather than standard treatments for PD. The strongest finding was the improvement in SBM frequency with oral probiotics, supported by Moderate-certainty evidence.

Evidence for motor symptoms and quality of life was Low to Very Low certainty, and the network contained few direct comparisons between active treatments. FMT and synbiotics should not be recommended for motor control on the basis of SUCRA rankings alone. Longer and adequately powered head-to-head trials are needed.

### Strengths and limitations

Several limitations affect the reliability and interpretation of this network meta-analysis:

Sparse network and indirect evidence: Most trials compared an active treatment with placebo or routine care, with few head-to-head comparisons between active treatments. Consequently, SUCRA rankings depend mainly on indirect evidence and should not be interpreted as proof of clinical superiority.

Intervention node merging: **FMT delivery routes and probiotic regimens with vitamin D, prebiotic fiber, or exercise were pooled to preserve network connectivity. These co-interventions were not always matched in the comparator arms. The pooled estimates may therefore reflect bundled regimens and may conceal route- or formulation-specific effects. The small number of trials prevented reliable regimen-specific sensitivity analyses**.

Descriptive heterogeneity and unconfirmed transitivity: Disease severity, levodopa equivalent daily dose, baseline motor scores, and follow-up duration were incompletely or inconsistently reported. Quantitative meta-regression was therefore not feasible. Residual effect modification and confounding across indirect comparisons cannot be excluded, so complete transitivity cannot be confirmed.

Correlation assumption for change-score SDs: When study-specific correlations were unavailable, R = 0.5 was used as a pragmatic moderate-correlation assumption. Alternative correlations were not examined across all derived SDs, and this may affect the precision of some continuous-outcome estimates.

Prediction intervals and contribution matrices: These analyses were not performed or reported. Their absence limits assessment of the expected range of effects in future studies and the contribution of individual direct comparisons to each network estimate.

Treatment duration and small studies: Treatment and follow-up ranged from single administration to 24 weeks, and several networks included small trials. Long-term efficacy, disease-modifying effects, and publication bias could not be assessed reliably.

## Conclusion

Oral probiotics increased SBM frequency in patients with PD, supported by Moderate-certainty evidence. Evidence for MDS-UPDRS III and PDQ-39 was Low to Very Low certainty and did not show significant benefits over placebo in the primary analyses. FMT and synbiotics should therefore be considered exploratory for motor outcomes. Larger, longer, head-to-head randomized trials are required.

## Data Availability

The original contributions presented in the study are included in the article/Supplementary material, further inquiries can be directed to the corresponding author.
