# Supplementary material for: Comparative efficacy of gut microbiota-targeted therapies for motor and non-motor symptoms in Parkinson's disease: a systematic review and network meta-analysis
Source: Front Microbiol. 2026 Oct 26;17:1907410. doi: 10.3389/fmicb.2026.1907410 (PMC13745344; doi:10.3389/fmicb.2026.1907410)

Appendix 1: Search strategy

Table S1. Search strategy of PubMed

#	Searches
1	((((((((((Parkinson's disease[MeSH Terms]) OR (Idiopathic Parkinson Disease[Title/Abstract])) OR (Idiopathic Parkinson's Disease[Title/Abstract])) OR (Lewy Body Parkinson Disease[Title/Abstract])) OR (Lewy Body Parkinson's Disease[Title/Abstract])) OR (Paralysis Agitans[Title/Abstract])) OR (Parkinson Disease, Idiopathic[Title/Abstract])) OR (Parkinson's Disease[Title/Abstract])) OR (Parkinson's Disease, Idiopathic[Title/Abstract])) OR (Parkinson's Disease, Lewy Body[Title/Abstract])) OR (Primary Parkinsonism[Title/Abstract])) OR (Parkinsonism, Primary[Title/Abstract])
2	((Prebiotics[MeSH Terms]) OR (Prebiotic*[Title/Abstract]))
3	((Probiotics[MeSH Terms]) OR (Probiotic*[Title/Abstract]))
4	((Synbiotics[MeSH Terms]) OR (Synbiotic*[Title/Abstract]))
5	("Fecal Microbiota Transplantation"[MeSH Terms]) OR ("Fecal Microbiota Transplan*"[Title/Abstract]) OR ("Fecal Transplant*"[Title/Abstract]) OR ("Fecal Microbiome Transplan*"[Title/Abstract]) OR ("Intestinal Microbiota Transplan*"[Title/Abstract]) OR (FMT[Title/Abstract])
6	#2 OR #3 OR#4 OR #5
7	#1 AND #6
8	((randomized controlled trial[pt]) OR (controlled clinical trial[pt]) OR (randomized[tiab]) OR (placebo[tiab]) OR (clinical trials as topic[mesh:noexp]) OR (randomly[tiab]) OR (trial[ti])) NOT (animals[mh] NOT humans[mh])
9	#7 AND #8

Table S2. Search strategy of Embase

#	Searches
1	'parkinson disease'/exp OR (parkinson* NEAR/3 (disease* OR symptom* ORism)):ti,ab
2	'prebiotic agent'/exp OR prebiotic*:ti,ab
3	'probiotic agent'/exp OR probiotic*:ti,ab
4	'synbiotic agent'/exp OR synbiotic*:ti,ab
5	'feces transplantation'/exp OR ('fecal microbiota transplan*':ti,ab) OR ('fecal transplan*':ti,ab) OR (fmt:ti,ab)
6	#2 OR #3 OR #4 OR #5
7	#1 AND #6
8	('randomized controlled trial'/exp OR 'randomization'/exp OR 'double blind procedure'/exp OR 'single blind procedure'/exp OR 'placebo'/exp OR random*:ti,ab OR factorial*:ti,ab OR crossover*:ti,ab OR (cross NEXT/1 over*):ti,ab OR placebo*:ti,ab OR (doubl* NEXT/1 blind*):ti,ab OR (singl* NEXT/1 blind*):ti,ab OR assign*:ti,ab OR allocat*:ti,ab OR volunteer*:ti,ab) NOT ('animal'/exp NOT 'human'/exp)
9	#7 AND #8

Table S3. Search strategy of Cochrane Library

#	Searches
1	[mh "Parkinson Disease"]
2	(Parkinson*):ti,ab,kw
3	#1 OR #2
4	[mh Prebiotics]
5	(Prebiotic*):ti,ab,kw
6	[mh Probiotics]
7	(Probiotic*):ti,ab,kw
8	[mh Synbiotics]
9	(Synbiotic*):ti,ab,kw
10	[mh "Fecal Microbiota Transplantation"]
11	("Fecal Microbiota Transplan*" OR "Fecal Transplan*" OR "Fecal Microbiome Transplan*" OR FMT):ti,ab,kw
12	#4 OR #5 OR #6 OR #7 OR #8 OR #9 OR #10 OR #11
13	#3 AND #12

Table S4. Search strategy of Web of Science

#	Searches
1	TS=(Parkinson* OR "Paralysis Agitans" OR "Primary Parkinsonism")
2	TS=(Prebiotic*)
3	TS=(Probiotic*)
4	TS=(Synbiotic*)
5	TS=("Fecal Microbiota Transplan*" OR "Fecal Transplan*" OR "Fecal Microbiome Transplan*" OR "Intestinal Microbiota Transplan*" OR FMT)
6	#2 OR #3 OR #4 OR #5
7	#1 AND #6
8	TS=(randomized controlled trial* OR "clinical trial*" OR randomi* OR placebo* OR "double-blind*" OR "single-blind*")
9	#7 AND #8

Table S5. Search strategy of CNKI (China National Knowledge Infrastructure)

#	Searches	
1	Search Pool	Search Formulation (in Chinese)
2	Disease (Parkinson's Disease)	SU = ('帕金森' OR '震颤麻痹' OR '路易体' OR '帕金森综合征')
3	Intervention A (Prebiotics)	SU = ('益生元' OR 'prebiotic')
4	Intervention B (Probiotics)	SU = ('益生菌' OR 'probiotic')
5	Intervention C (Synbiotics)	SU = ('合生元' OR '同生元' OR 'synbiotic')
6	Intervention D (FMT)	SU = ('粪菌移植' OR '肠道菌群移植' OR '粪便微生物移植' OR 'FMT' OR '微生态')
7	All Microbial Interventions	#2 OR #3 OR #4 OR #5
8	Disease AND Interventions	#1 AND #6
9	Study Design (RCT Filter)	SU = ('随机对照试验' OR '随机' OR '对照' OR '盲法' OR '安慰剂' OR 'RCT')

Appendix 2: Characteristics of included studies

Table S2.1: Baseline of characteristics of included studies

Study	Trial registration	Country and region	Design	Follow-up duration	Number of participants (T/C)	Randomised treatments (T/C)	Duration (mean±SD), years	Mean Age	Male %	Mean BMI	H-Y
Zhang R	NR	China	RCT	3 weeks	72 (36/36)	dFMT vs. aFMT	2.5±1.2 (dFMT)	64.2±7.8 (dFMT)	54.5%	22.8±2.4	I-III
Wang J	ChiCTR2100050380	China	RCT	Single dose	30 (15/15)	Fresh FMT vs. Placebo	3.0 (median)	68.0 (median)	60%	22.1±2.3	I-III
Figura	NCT05204641	Poland	RCT	Single dose	59 (28/31)	Mbiotix FMT vs. Placebo	6.5 (median)	64.0 (median)	49.2%	25.5	NR
Du Y	ChiCTR2200065638	China	RCT	12 weeks	50 (25/25)	Probiotics vs. Control	5.8±4.3 (Pro)	63.8±8.2 (Pro)	56%	23.4±3.1	I-III
Liu J	NR	China	RCT	4 weeks	51 (25/26)	FMT capsule vs. Placebo	5.3±2.1 (FMT)	65.4±6.8 (FMT)	51.0%	NR	≤II
Cheng Y	ChiCTR1900021405	China	RCT	12 weeks	54 (27/27)	Oral FMT vs. Placebo	6.7±4.0 (FMT)	60.5±8.7 (FMT)	59.3%	22.3±2.2	I-III
Wang SY	NR	China	RCT	16 weeks	39 (20/19)	Triple Probiotics vs. Control	6.2±2.4 (Joint)	64.2±7.5 (Joint)	51.3%	NR	NR
Zali A	IRCT20200921048793N1	Iran	RCT	12 weeks	46 (23/23)	Probiotics+VitD vs. Placebo	7.8±4.5 (Pro)	61.2±8.5 (Pro)	60.9%	26.5±3.8	≤III
Mehrabani	IRCT20180818040827N2	Iran	RCT	12 weeks	80 (40/40)	Synbiotic vs. Placebo	6.2±4.1 (Syn)	65.8±7.1 (Syn)	57.5%	26.3±3.5	≤III (29)/≥III (11)
Ibrahim	NCT04451096	Malaysia	RCT	8 weeks	48 (22/26)	Multi-strain Probiotics vs. Placebo	6.5±4.5 (Pro)	64.0±8.0 (Pro)	43.8%	24.3±4.5	I-IV
Tan AH	NR	Malaysia	RCT	4 weeks	72 (34/38)	Multi-strain Probiotics vs. Placebo	5.8±4.8 (Pro)	65.1±8.1 (Pro)	62.5%	24.1±3.9	NR
DuPont	NR	USA	RCT	12 weeks	12 (8/4)	FMT vs. Placebo	6.0 (FMT)	68.0 (FMT)	50.0%	NR	≤III
Barichella	NCT02459717	Italy	RCT	4 weeks	120 (80/40)	Probiotics+Fiber vs. Placebo	6.7±4.8 (Pro)	68.5±7.3 (Pro)	54.2%	24.5±3.5	I-V
Ghalandari	IRCT20170608034390N11	Iran	RCT	8 weeks	30 (15/15)	Probiotics (Comflor) vs. Placebo	6.5±3.2 (Pro)	62.5±7.5 (Pro)	60.0%	26.8±4.2	I-IV
Fang YK	NR	China	RCT	12 weeks	60 (30/30)	Probiotics vs Placebo	5.5±2.3	64.5±8.2	53.3%	NR	NR
Yang J	NR	China	RCT	24 weeks	154 (77/77)	Probiotics+movement vs movement	3.2±1.1 (Pro)	62.5±6.8 (Pro)	55.8%	NR	I-III
Scheperjans	NCT04671030	Finland	RCT	12 weeks	45 (30/15)	FMT vs. Placebo	7.0 (median)	66.0 (median)	55.6%	25.8	≤II

Study	Trial registration	Country and region	Design	Follow-up duration	Number of participants (T/C)	Randomised treatments (T/C)	Duration (mean±SD), years	Mean Age	Male %	Mean BMI	H-Y
Ramadan ME	NR	Egypt	RCT	12 weeks	66 (33/33)	Synbiotic vs. Placebo	7.1±3.9 (Pro)	56.06±6.2 (Syn)	56.7%	26.1±3.4	I-IV
Valentina Leta	NR	UK	RCT	12 weeks	68 (35/33)	Probiotic vs. Placebo	6.8±4.2 (Pro)	68.17±8.29(Pro)	55.0%	25.9±3.7	II-III

Appendix 3: Risk of bias of randomized clinical trials

Figure S3: Overall risk of bias presented as percentage of each risk of bias item across all included studies. Green = Low risk, Red = High risk, Yellow = Some concerns

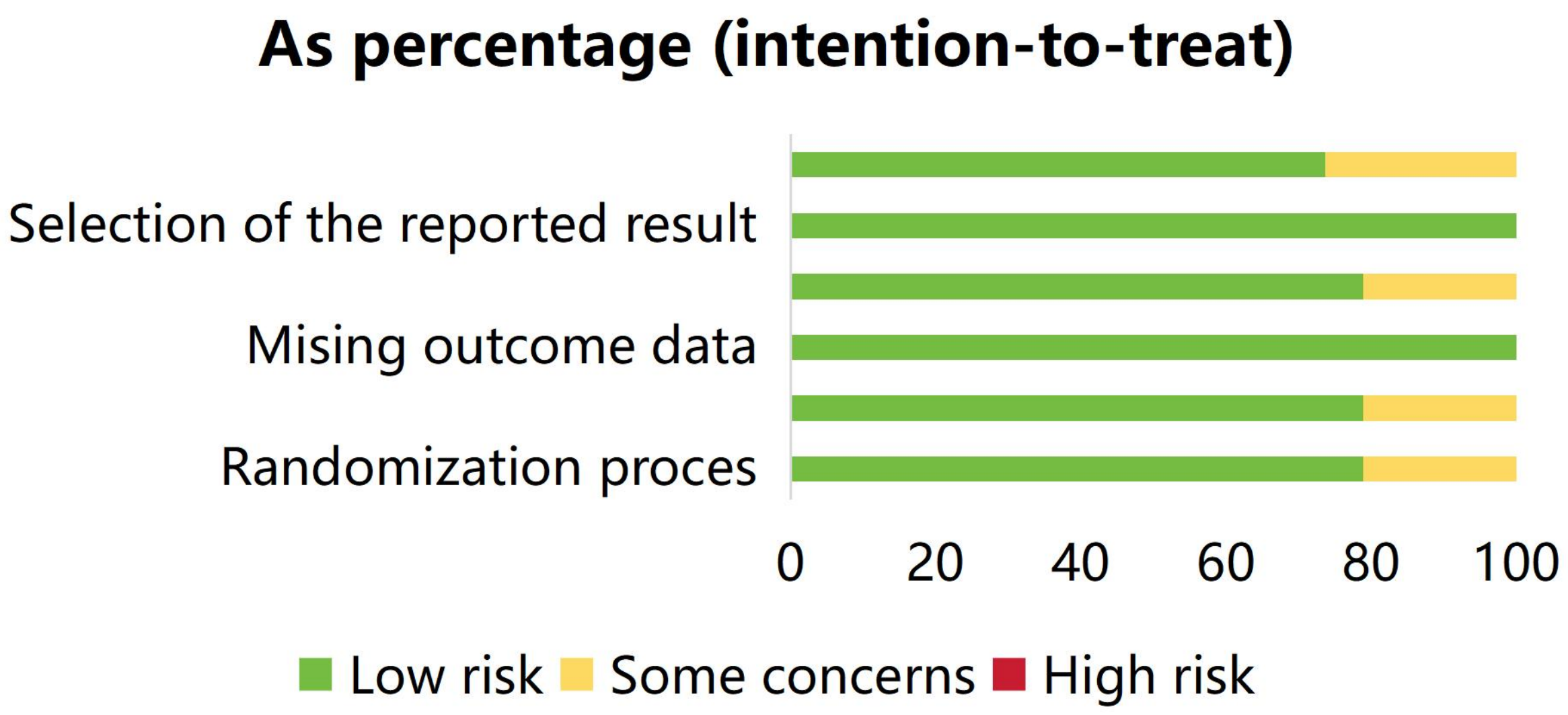

Table S3：Study level risk of bias assessment using Cochrane risk of bias tool 2.0 for assessing risk of bias of randomized clinical trials.

Unique ID	Study ID	Randomization proces	Deviations from intended interventions	Missing outcome data	Measurement of the outcom	Selection of the reported result	Over all
Alireza Zali12024	NR	low	low	low	low	low	Low Risk
Filip Scheperjans2024	ChiCTR2100050380	low	low	low	low	low	Low Risk
Ai Huey Tan2021	NCT05204641	low	low	low	low	low	Low Risk
Monika Figura	ChiCTR2200065638	low	low	low	low	low	Low Risk
Sanaz Mehrabani2023	NR	low	low	low	low	low	Low Risk
Mohamed E. Ramadan2025	ChiCTR1900021405	low	low	low	low	low	Low Risk
Azliza Ibrahim2020	NR	low	low	low	low	low	Low Risk
Valentina Leta	IRCT20200921048793N1	Some concerns	Some concerns	low	Some concerns	low	Some concerns
Herbert L.DuPont	IRCT20180818040827N2	low	Some concerns	low	low	low	Some concerns
Michela Barichella2016	NCT04451096	low	low	low	low	low	Low Risk
Nasibeh Ghalandari2023	NR	low	low	low	low	low	Low Risk
Yitong Du2025	NR	low	low	low	low	low	Low Risk
Yi Cheng2023	NCT02459717	low	low	low	low	low	Low Risk
Jingyi Wang2025	IRCT20170608034390N11	low	low	low	low	low	Low Risk
Rui Zhang2026	NR	low	low	low	low	low	Low Risk
Wang Shaoying2020	NR	Some concerns	low	low	Some concerns	low	Some concerns
Yang Jie2025	NCT04671030	Some concerns	Some concerns	low	Some concerns	low	Some concerns
Fang Yika2022	NR	low	low	low	low	low	Low Risk
Liu Juan2023	NR	Some concerns	Some concerns	low	Some concerns	low	Some concerns

Appendix 4：Evaluation of inconsistency and heterogeneity

Table S4.1: Global consistency

Clinical outcome	Chi square	P value	τ^2network
UPDRS	1.85	0.1733	0.07
UPDRS III	0.80	0.3698	0.08
SBM	2.27	0.1318	0.08
Gastrointestinal Symptom	0.33	0.5664	0.08
Anxiety Score	0.03	0.8544	0.08
Depression Score	0.80	0.3702	0.08
Quality of Life Score	0.30	0.5816	0.08
Sleep Score	0.06	0.8123	0.08
Cognitive Function Score	4.04	0.5444	0.08
TAC	0.26	0.6075	0.08
MDA	1.33	0.2484	0.08

Appendix 5：Network maps and forest plots of secondary outcomes

Figure S5.1：Network map of the effect on UPDRS, and forest plot of network effect sizes for compared with placebo. The size of the nodes was proportional to the number of participants included in the trial, and the thickness of lines between the interventions relates to the number of studies for that comparison.

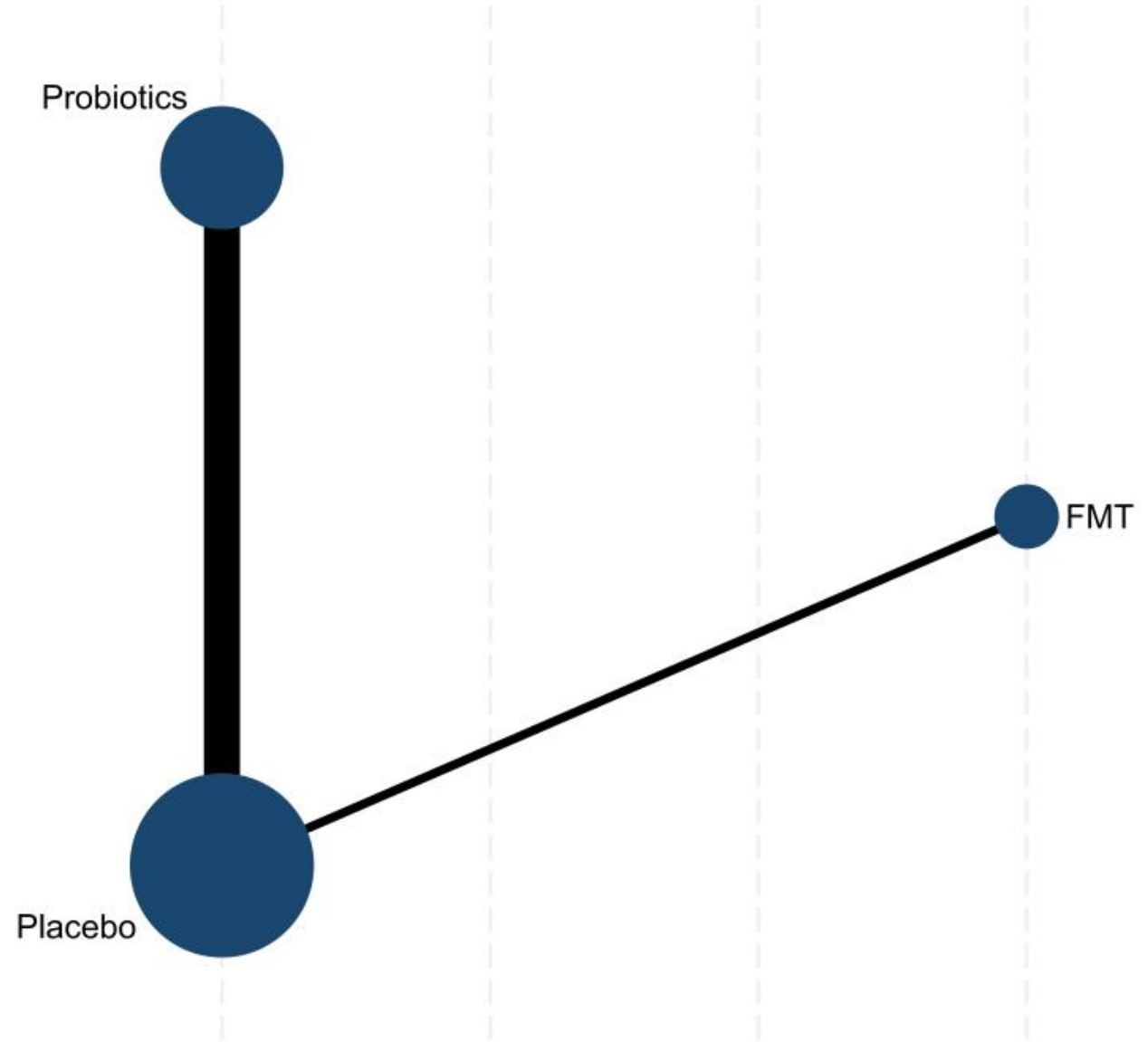

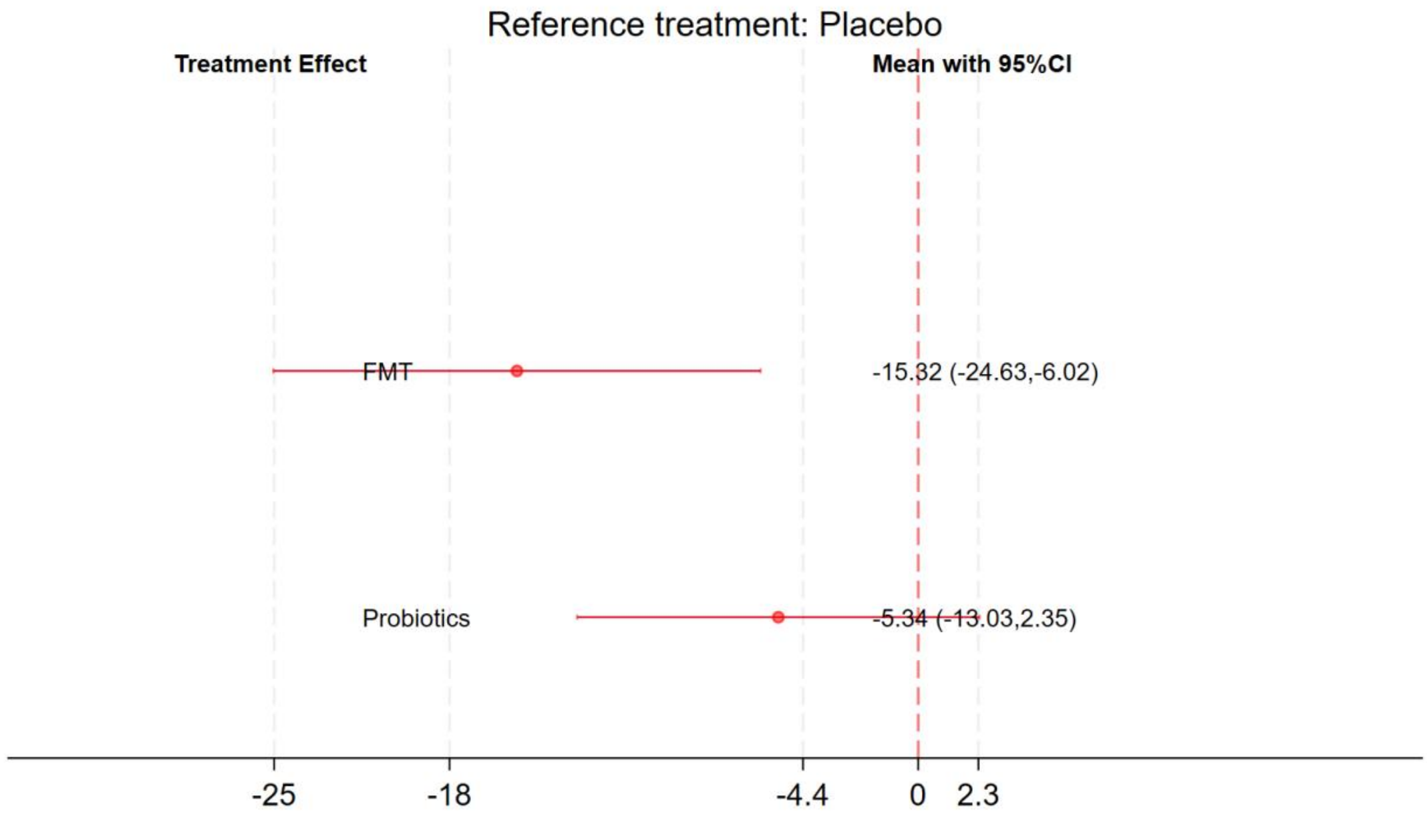

Figure S5.2：Network map of the effect on Anxiety Score, and forest plot of network effect sizes for compared with placebo. The size of the nodes was proportional to the number of participants included in the trial, and the thickness of lines between the interventions relates to the number of studies for that comparison.

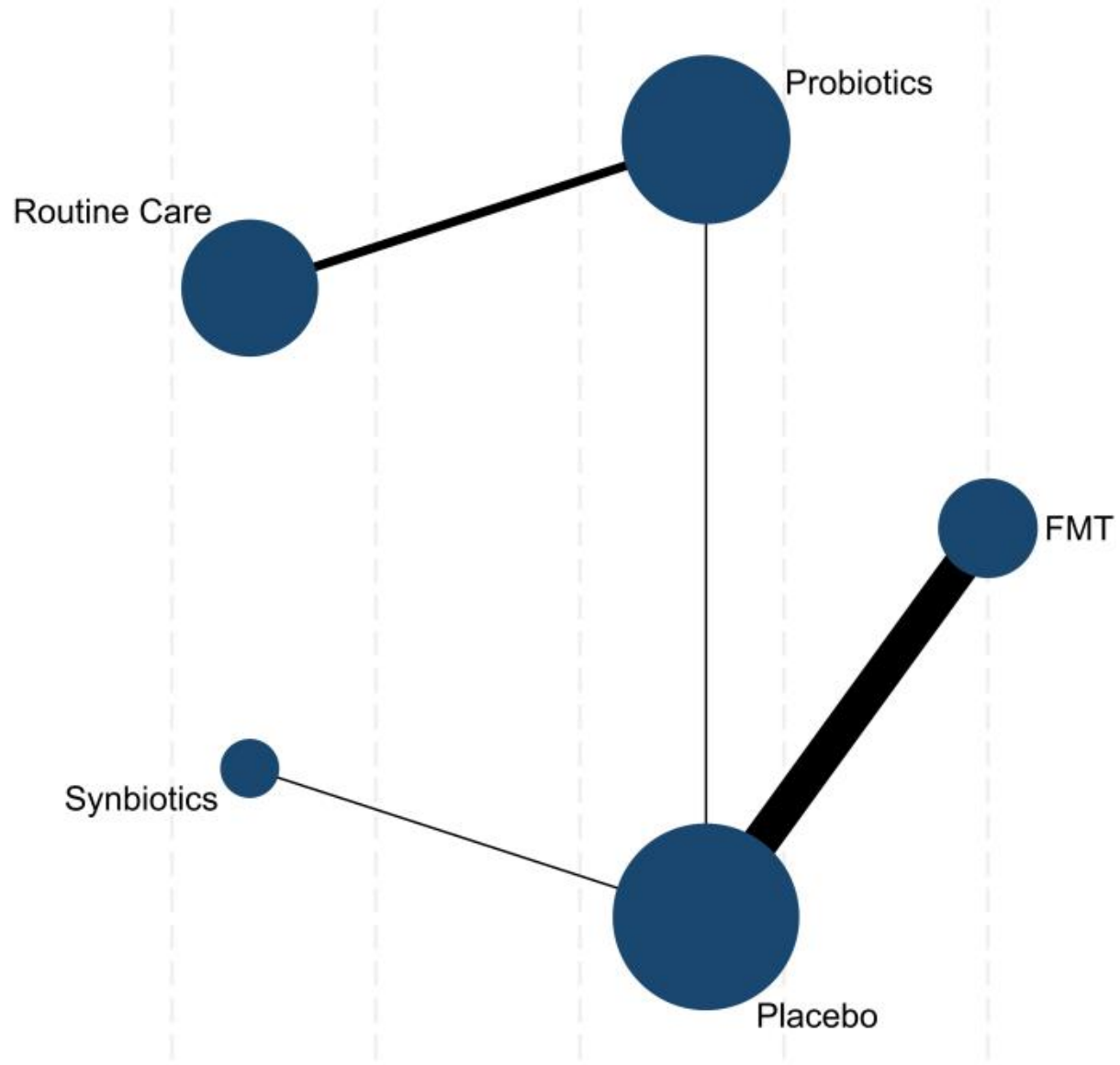

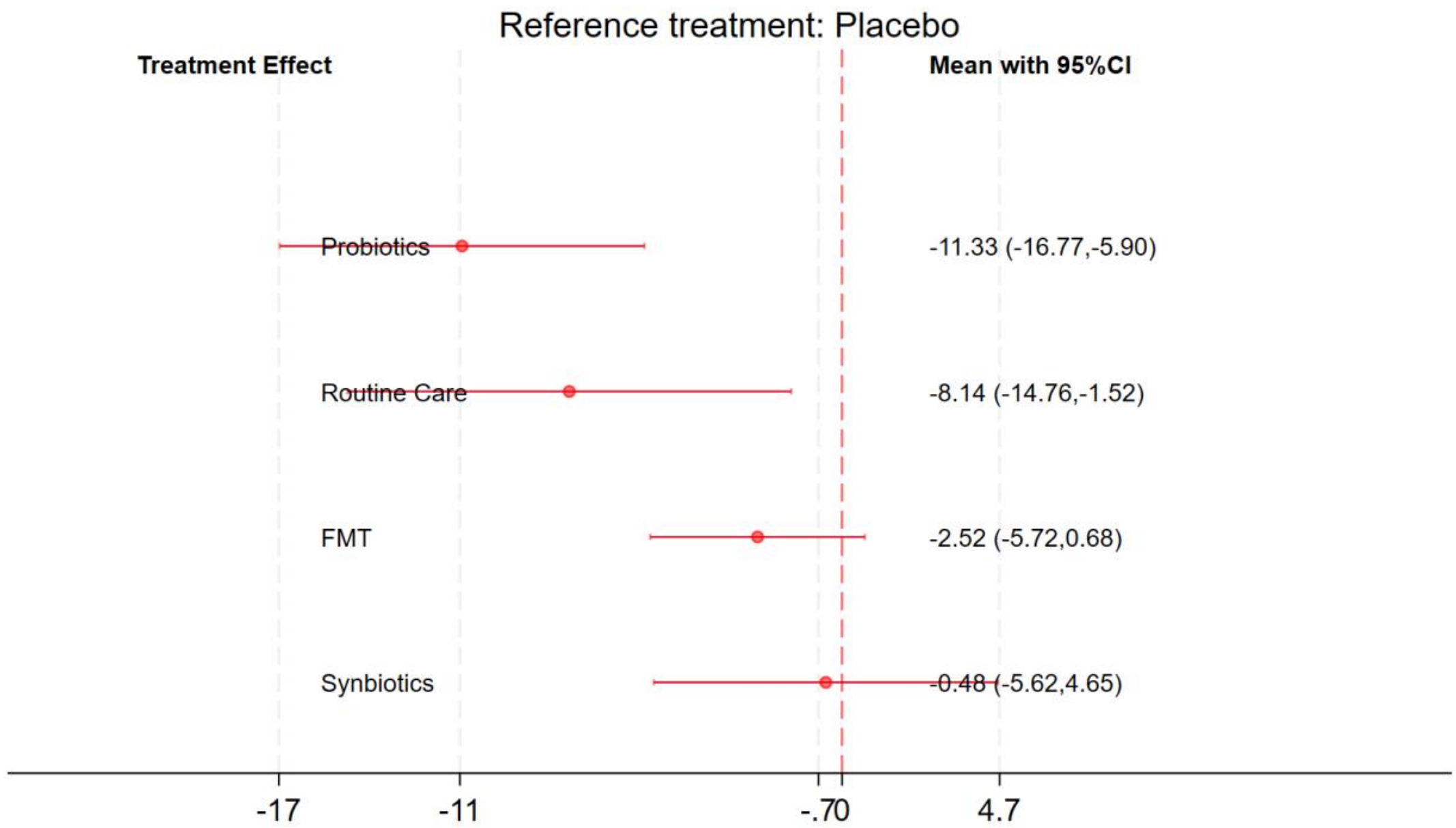

Figure S5.3： Network map of the effect on Depression Score, and forest plot of network effect sizes for compared with placebo. The size of the nodes was proportional to the number of participants included in the trial, and the thickness of lines between the interventions relates to the number of studies for that comparison.

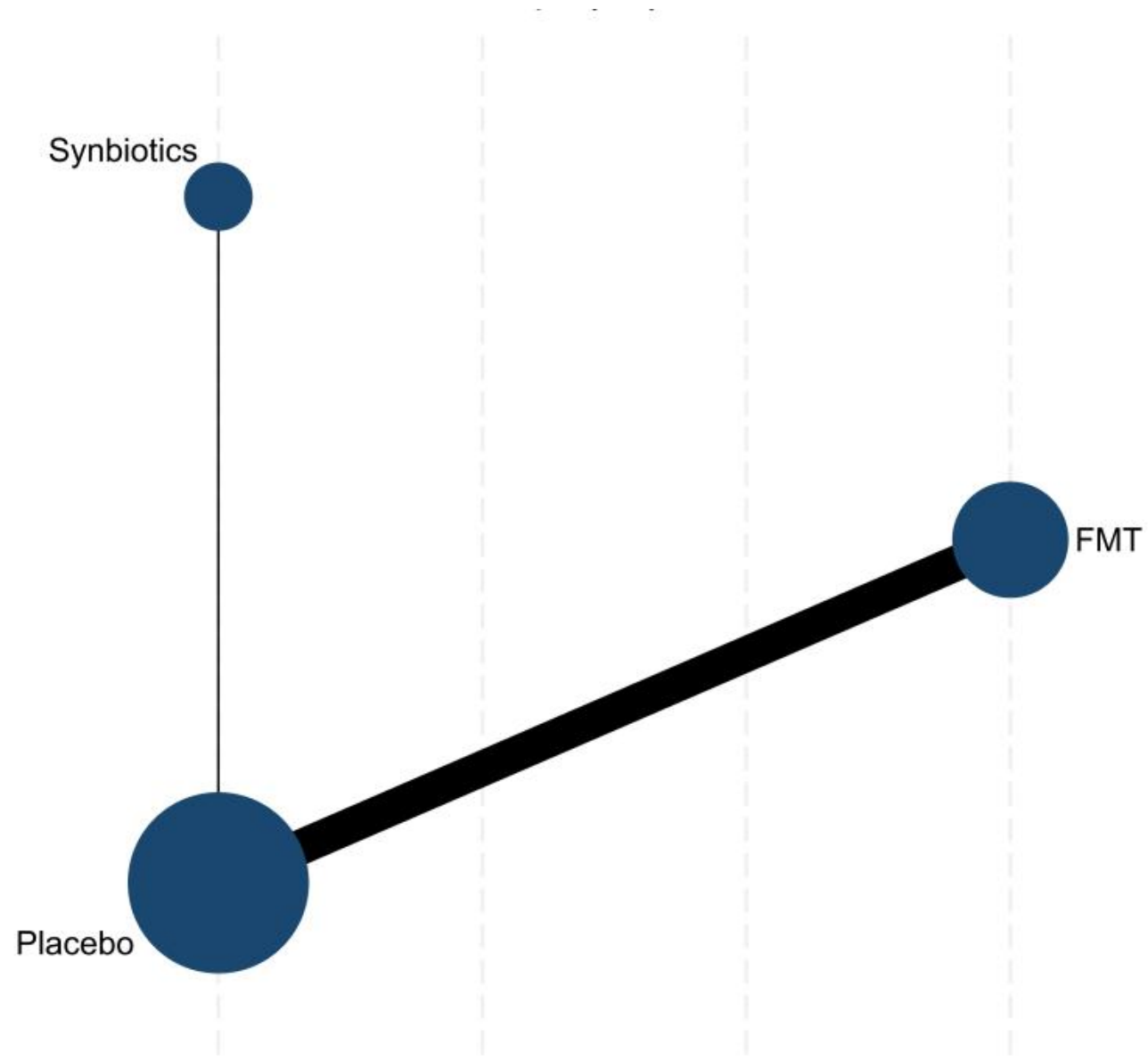

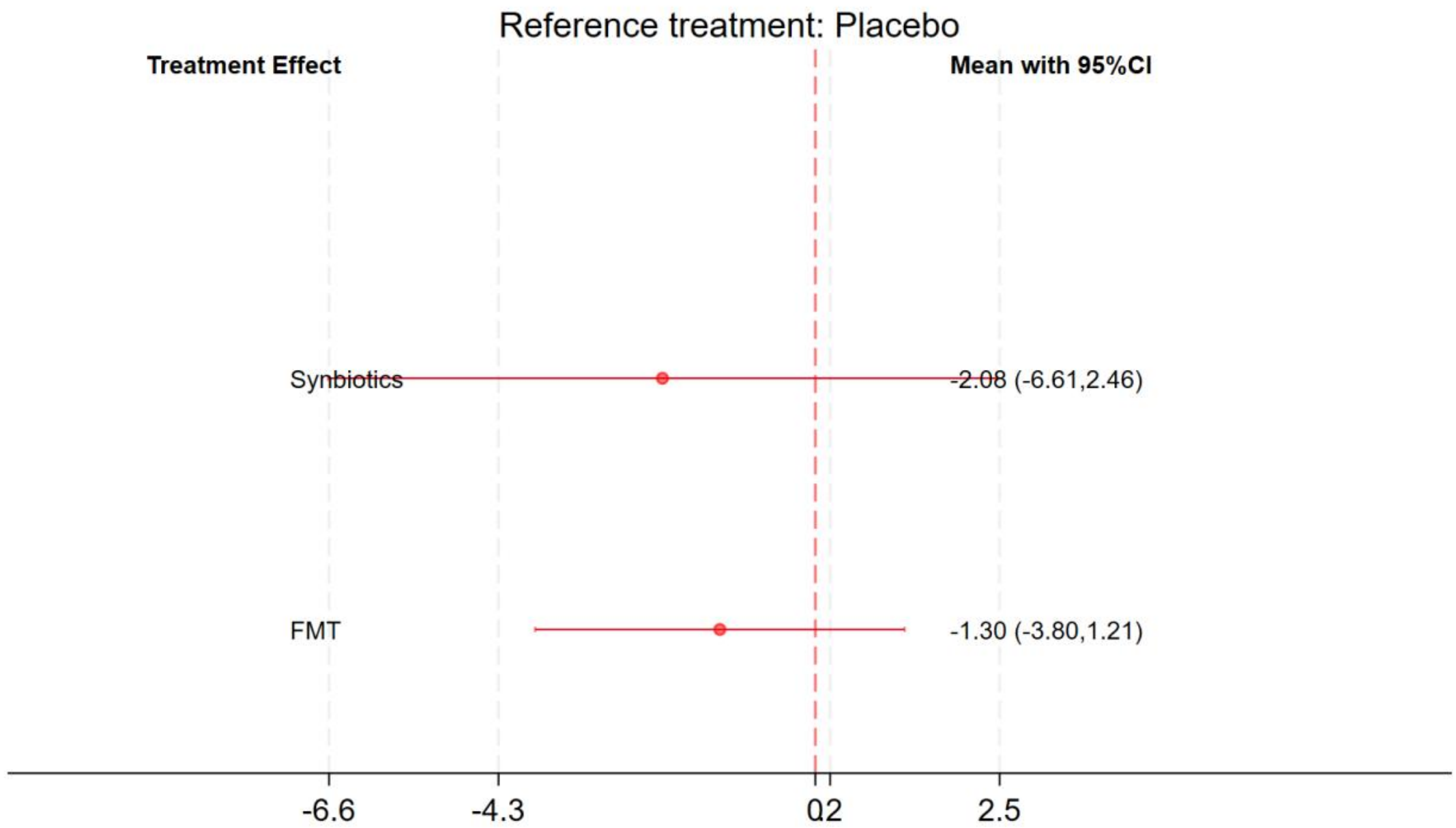

Figure S5.4：Network map of the effect on Sleep Score, and forest plot of network effect sizes for compared with placebo. The size of the nodes was proportional to the number of participants included in the trial, and the thickness of lines between the interventions relates to the number of studies for that comparison.

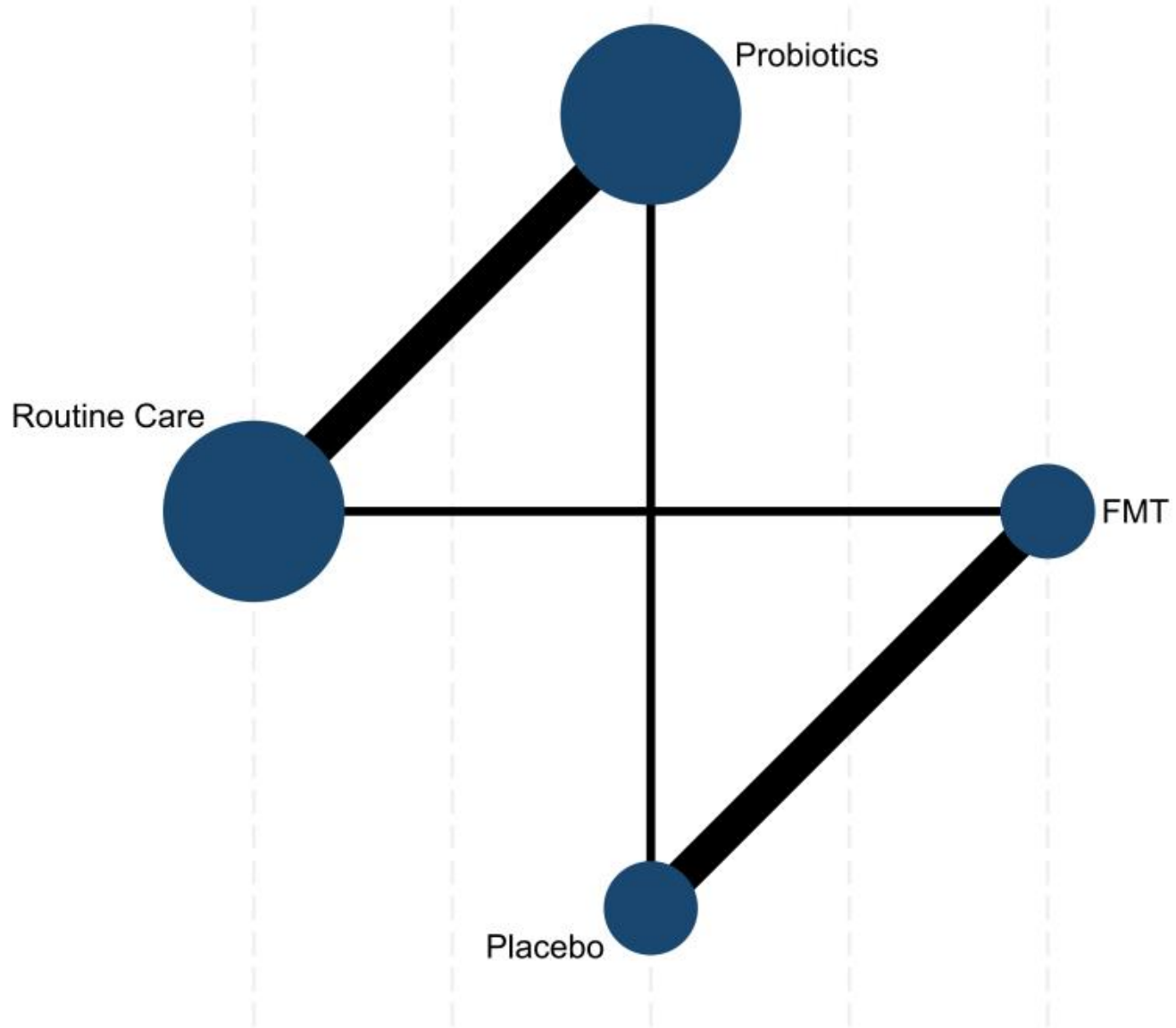

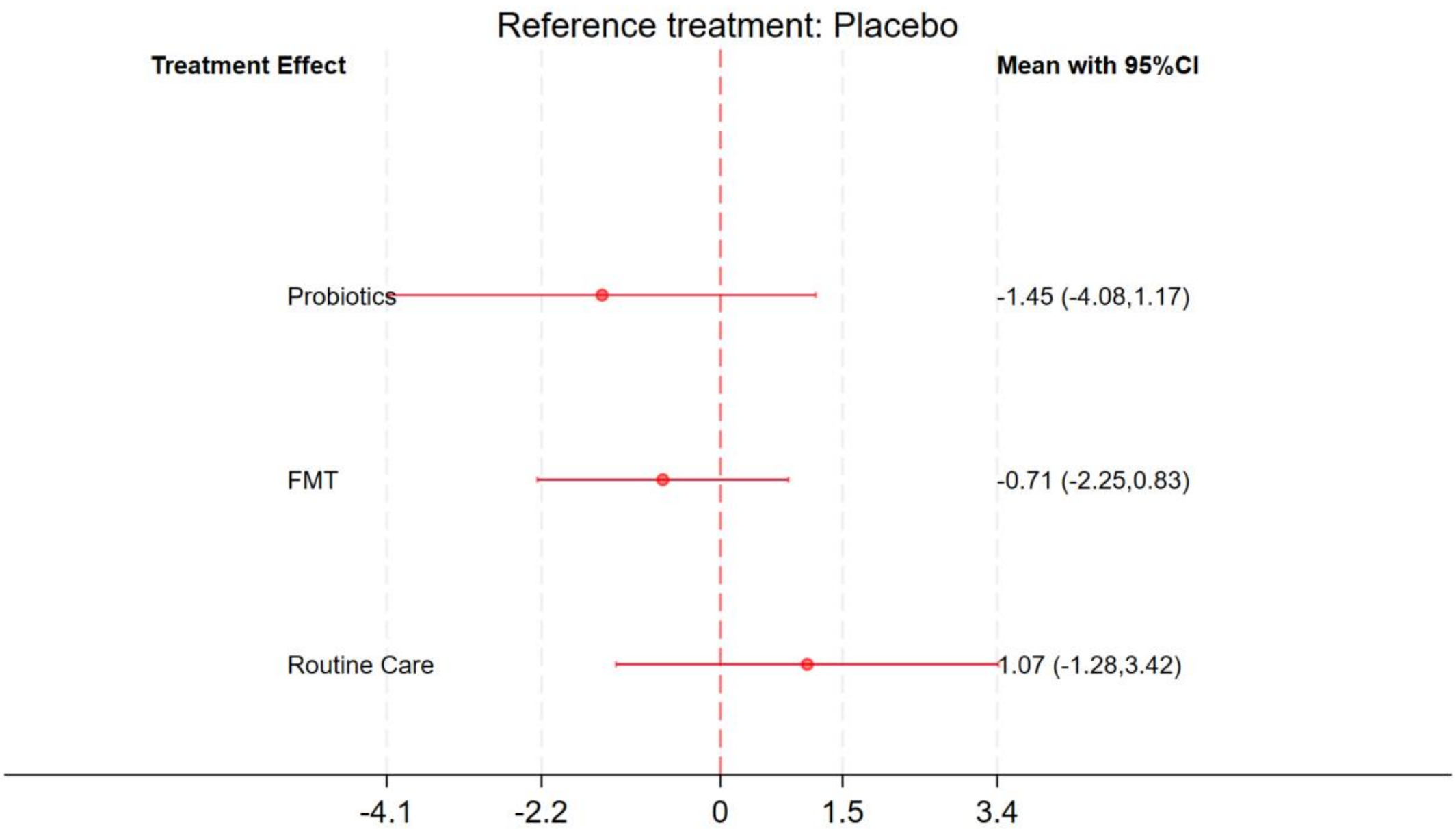

Figure S5.5：Network map of the effect on Cognitive Function Score, and forest plot of network effect sizes for compared with placebo. The size of the nodes was proportional to the number of participants included in the trial, and the thickness of lines between the interventions relates to the number of studies for that comparison.

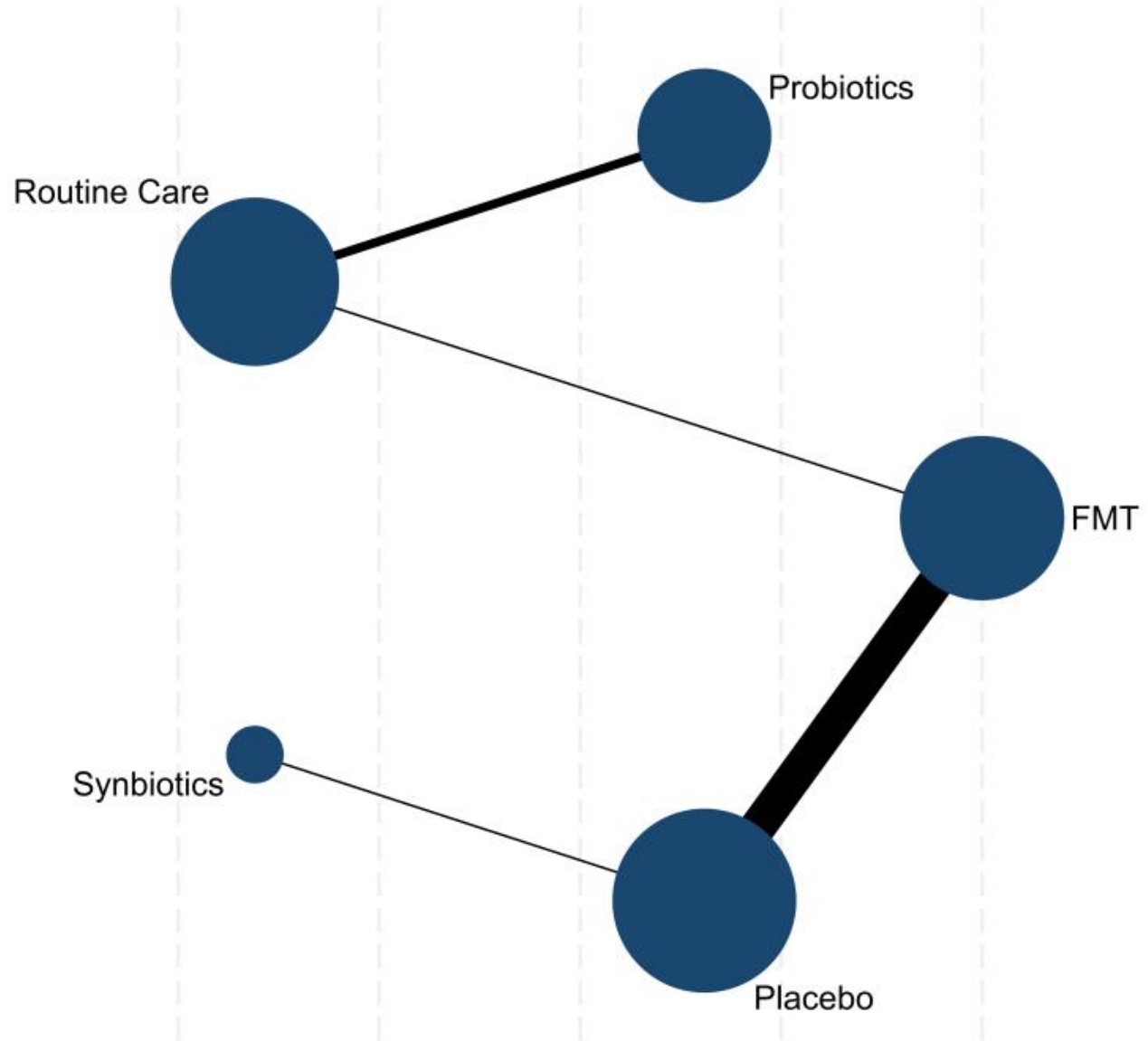

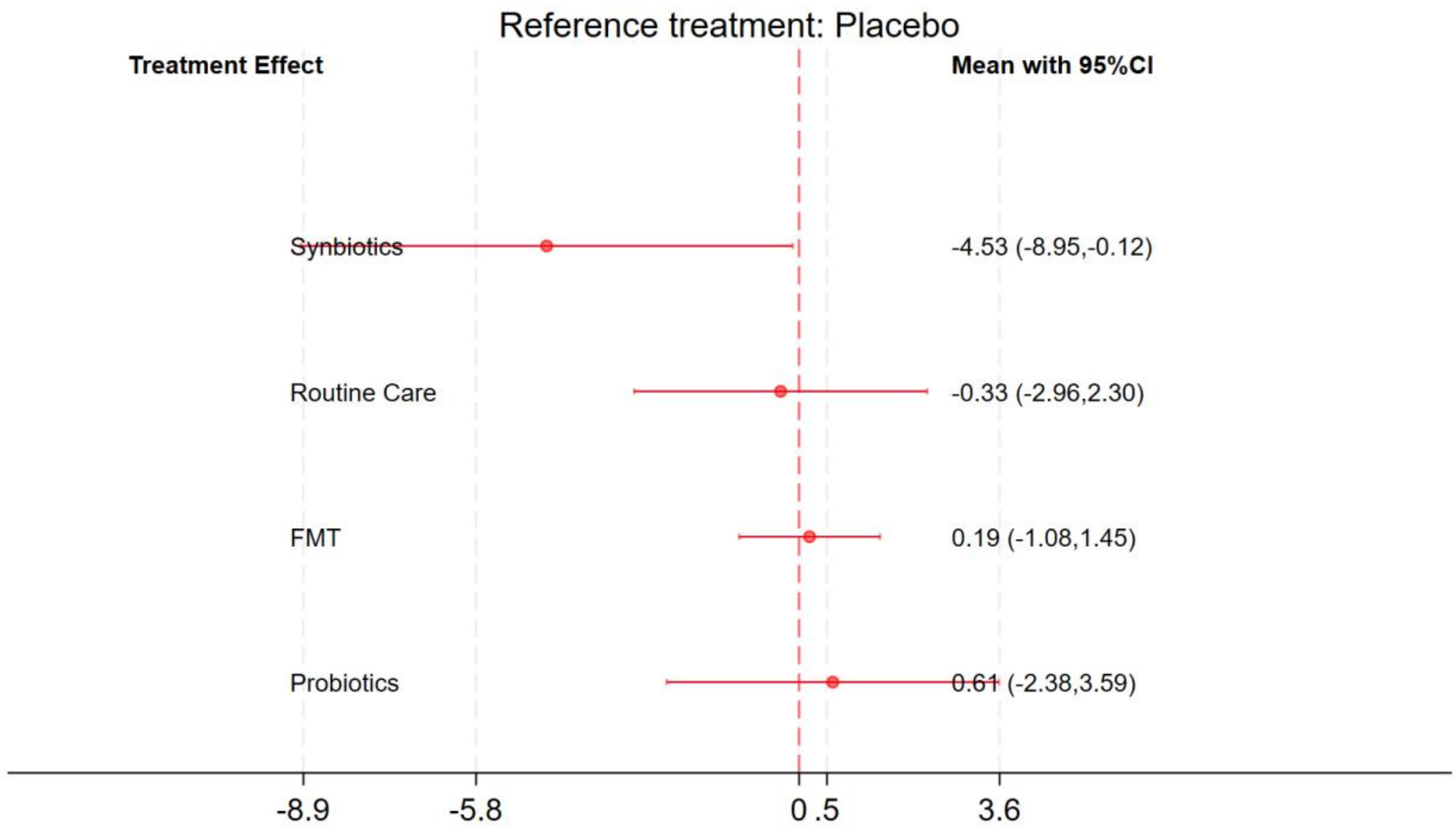

Figure S5.6： Network map of the effect on TAC, and forest plot of network effect sizes for compared with placebo. The size of the nodes was proportional to the number of participants included in the trial, and the thickness of lines between the interventions relates to the number of studies for that comparison.

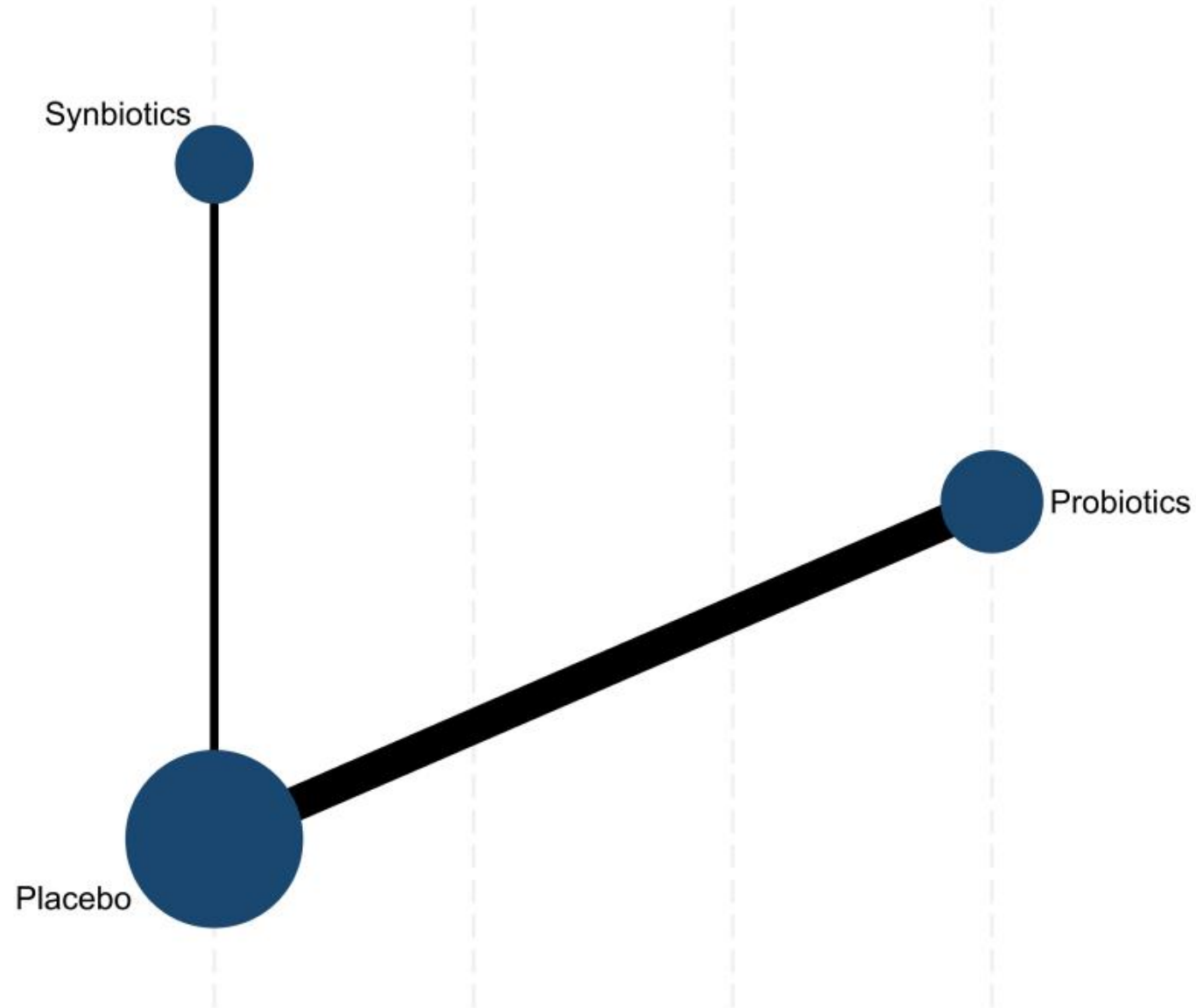

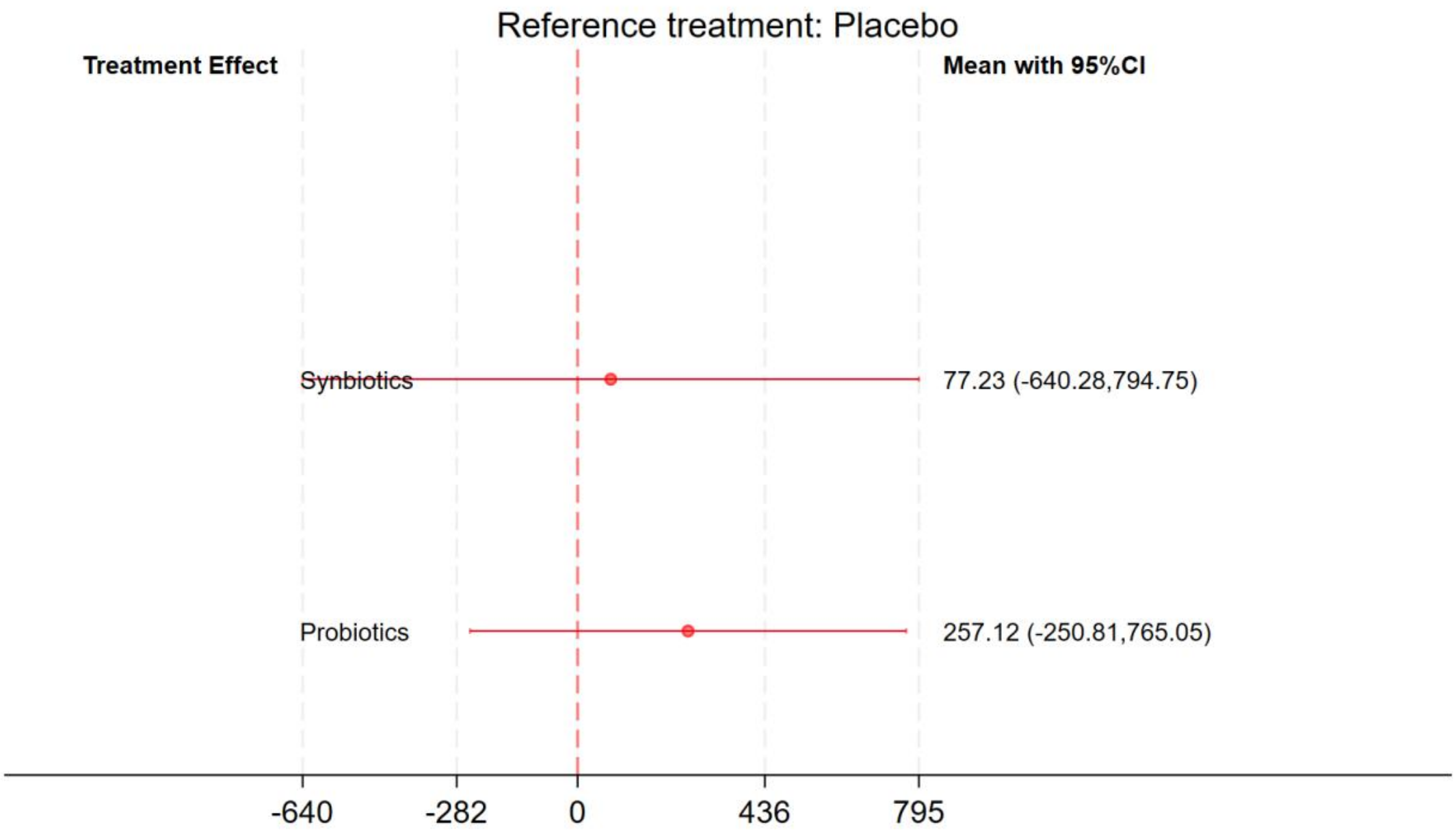

Figure S5.7： Network map of the effect on MDA, and forest plot of network effect sizes for compared with placebo. The size of the nodes was proportional to the number of participants included in the trial, and the thickness of lines between the interventions relates to the number of studies for that comparison.

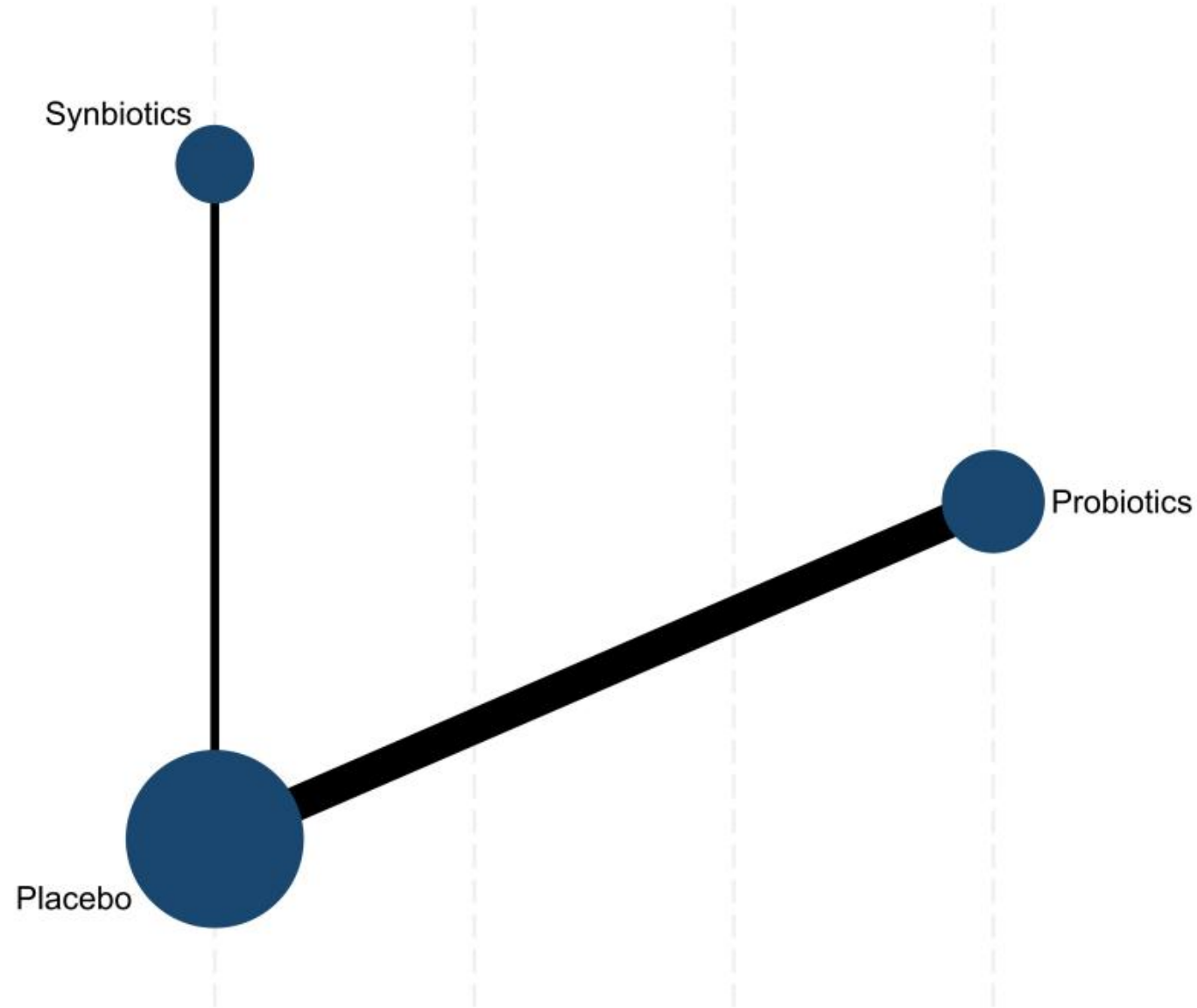

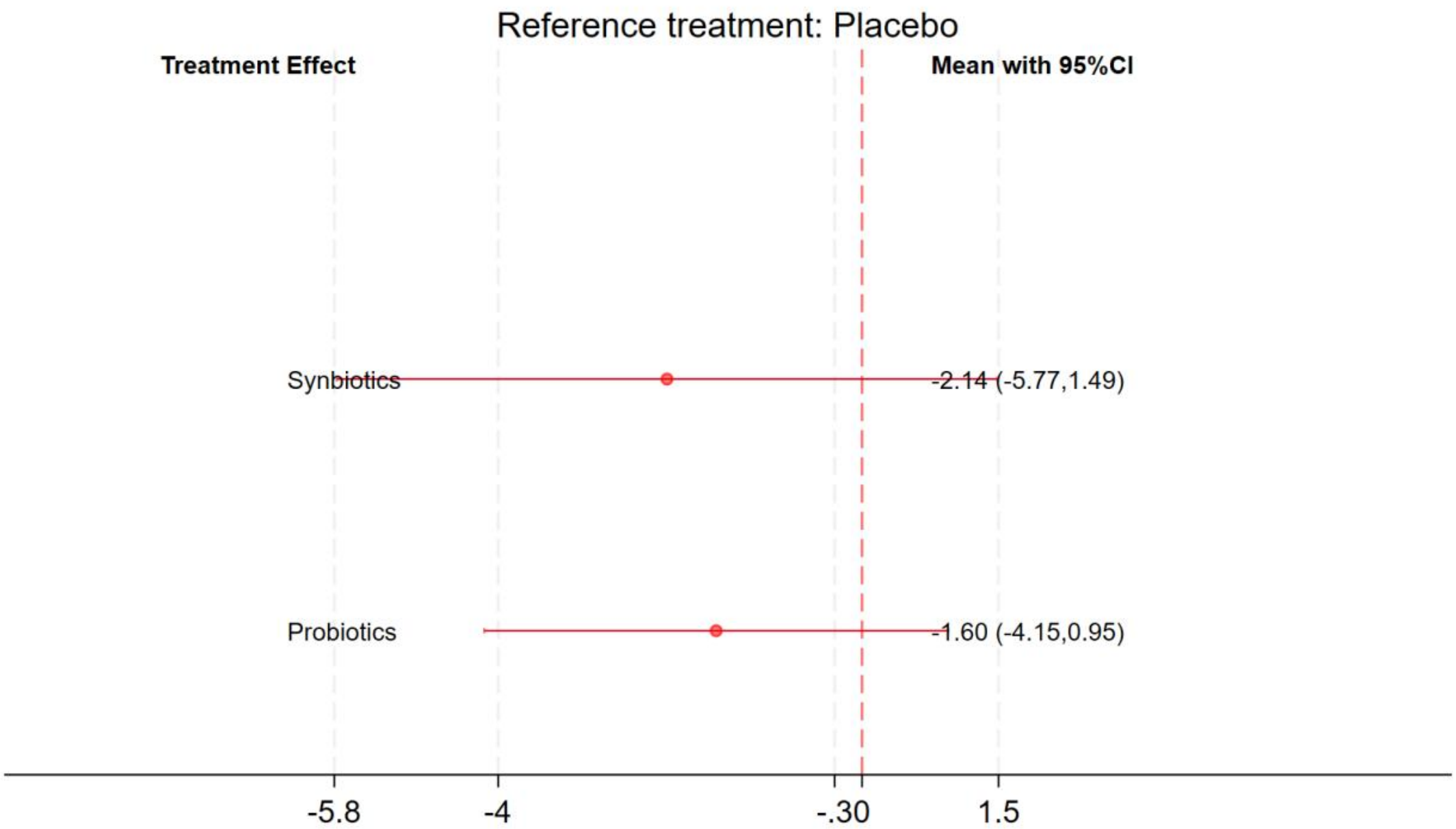

Appendix 6：SUCRA and cumulative probability plots

Figure S6.1：Cumulative ranking curve plots of gut microbiota-targeted therapies for UPDRS total score. Higher surface under the cumulative ranking curve (SUCRA) reflects a higher probability of association with a reduction in the UPDRS total score.

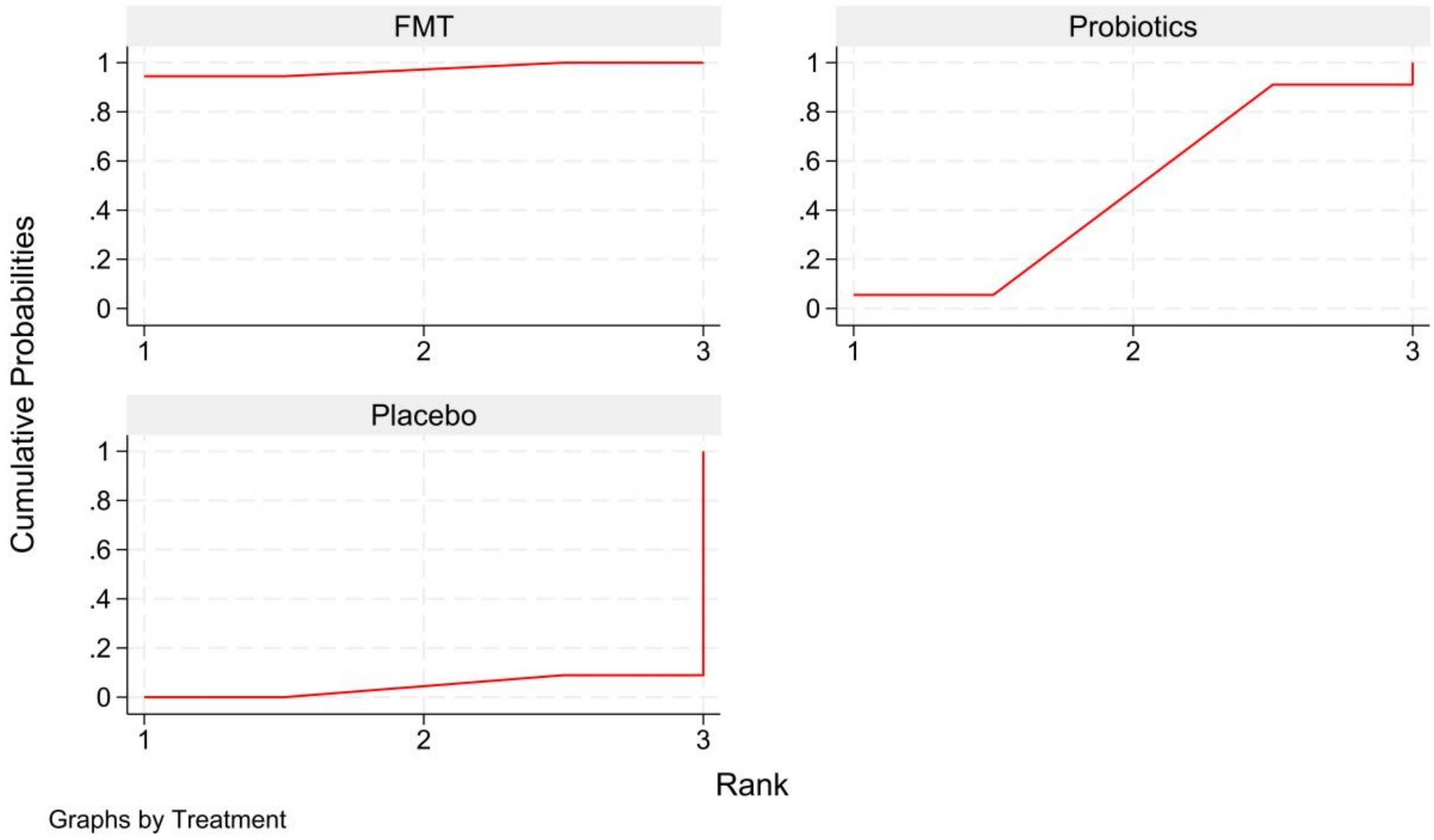

Table S6.1：SUCRA values and probability rankings of various gut microbiota-targeted therapies for on UPDRS total score.

Treatment	SUCRA	PrBest	MeanRank
FMT	97.2	94.5	1.1
Probiotics	48.3	5.5	2
Placebo	4.5	0	2.9

Figure S6.2：Cumulative ranking curve plots of gut microbiota-targeted therapies for UPDRS III score. Higher surface under the cumulative ranking curve (SUCRA) reflects a higher probability of association with a reduction in the UPDRS III score.

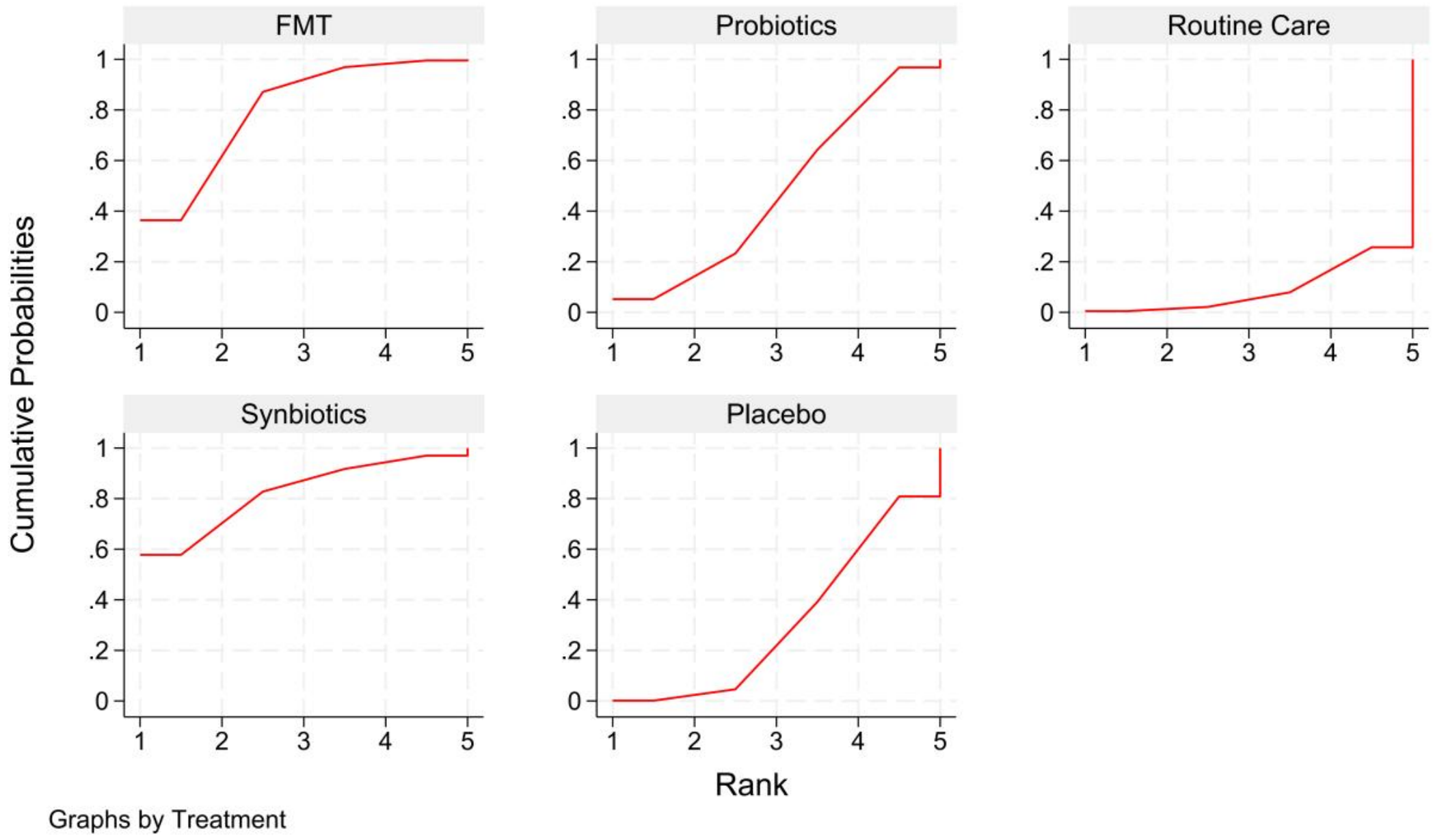

Table S6.2：SUCRA values and probability rankings of various gut microbiota-targeted therapies for UPDRS III score.

Treatment	SUCRA	PrBest	MeanRank
FMT	80	36.4	1.8
Probiotics	47.4	5.2	3.1
Routine Care	9.1	0.5	4.6
Synbiotics	82.3	57.8	1.7
Placebo	31.2	0.1	3.8

Figure S6.3：Cumulative ranking curve plots of gut microbiota-targeted therapies for SBM. A larger surface under the cumulative ranking curve (SUCRA) reflects a higher probability of being positioned as a more effective treatment for increasing SBM frequency.

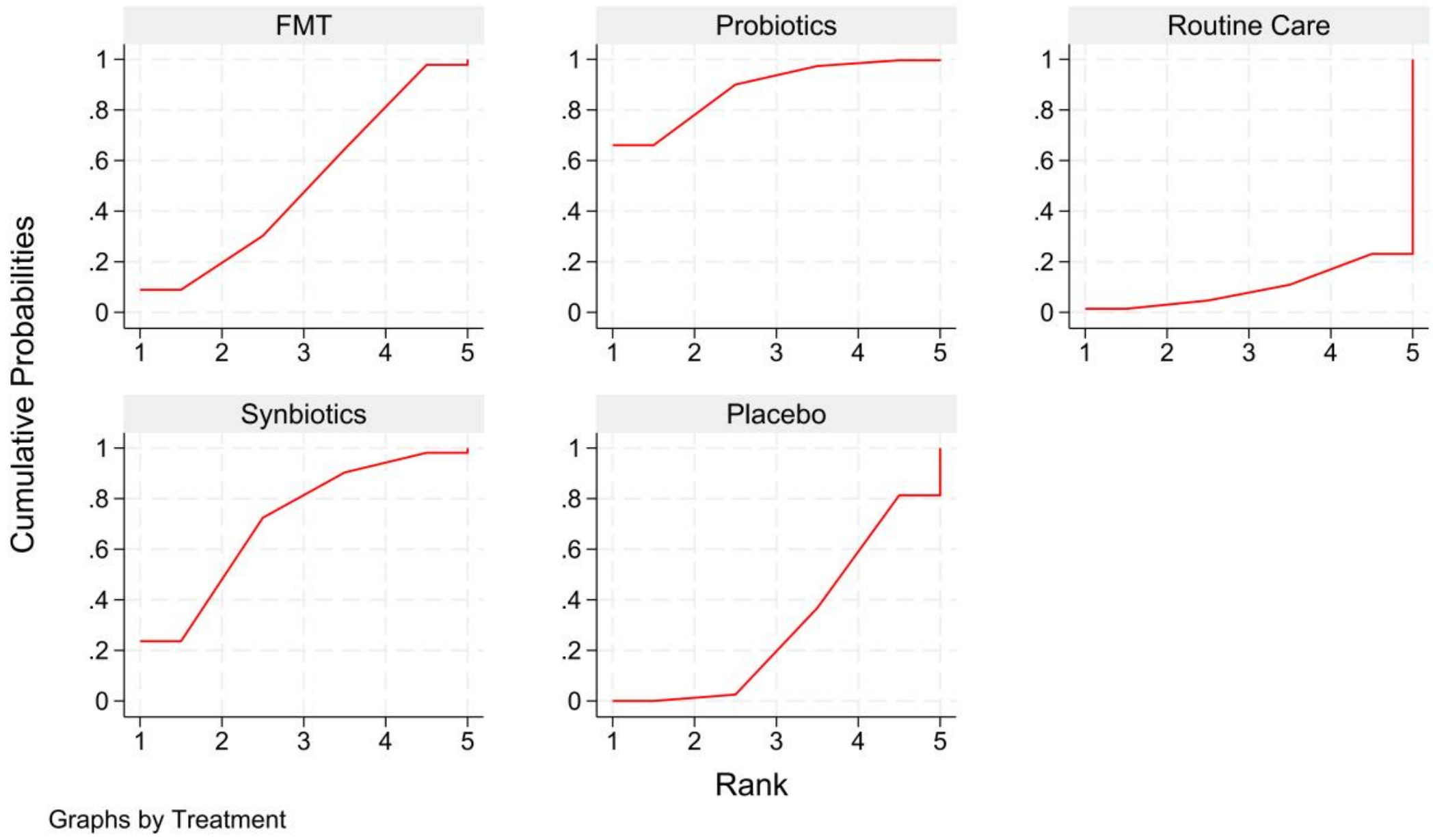

Table S6.3：SUCRA values and probability rankings of various gut microbiota-targeted therapies for SBM.

Treatment	SUCRA	PrBest	MeanRank
**FMT**	50.4	8.9	3
**Probiotics**	88.3	66.1	1.5
**Routine Care**	10	1.4	4.6
**Synbiotics**	71.1	23.6	2.2
**Placebo**	30.2	0	3.8

Figure S6.4 : Cumulative ranking curve plots of gut microbiota-targeted therapies for gastrointestinal symptoms. A larger surface under the cumulative ranking curve (SUCRA) reflects a higher probability of being positioned as a more effective treatment for reducing gastrointestinal symptom severity.

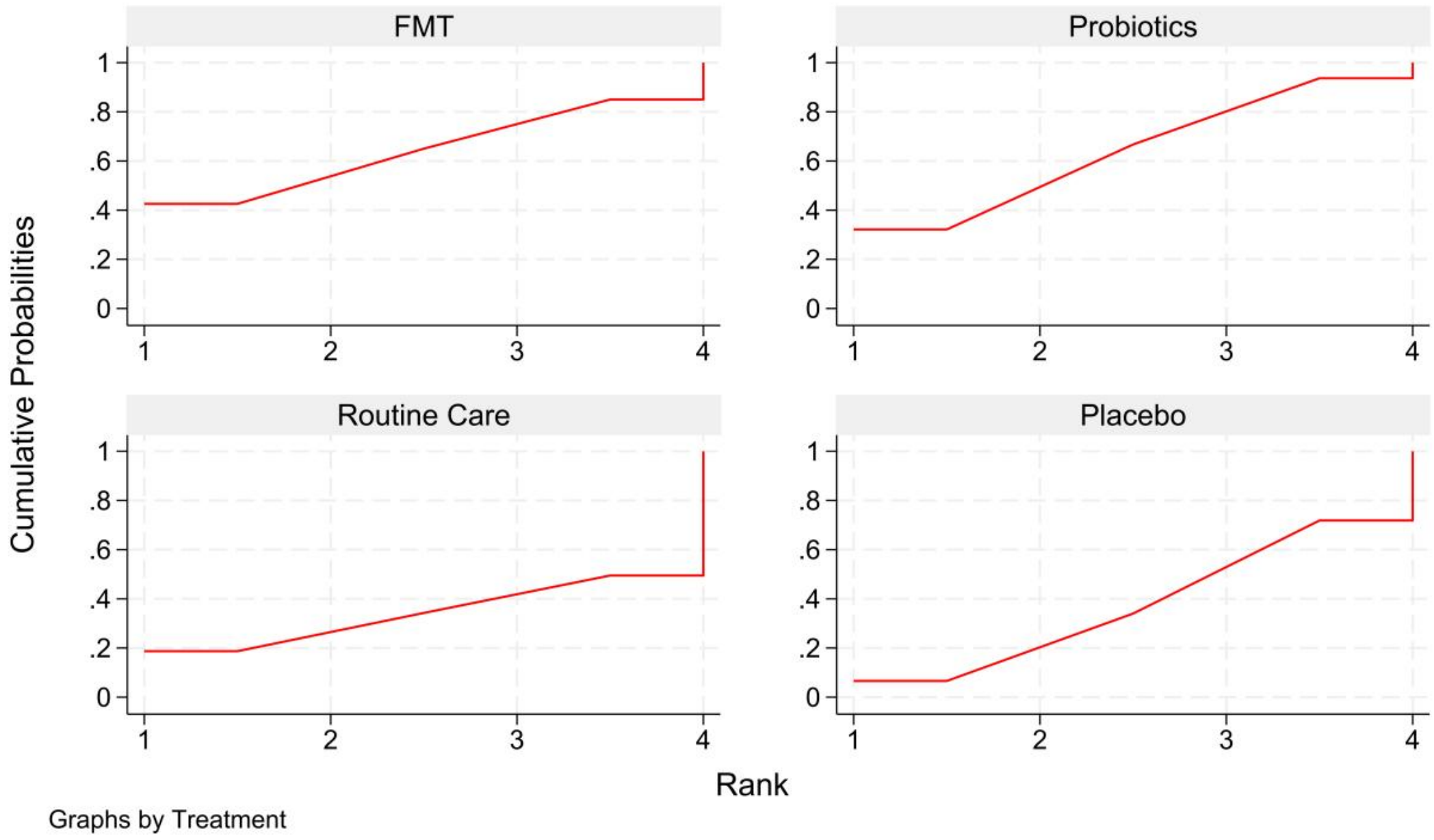

Table S6.4：SUCRA values and probability rankings of various gut microbiota-targeted therapies for gastrointestinal symptoms.

Treatment	SUCRA	PrBest	MeanRank
**FMT**	64.2	42.6	2.1
**Probiotics**	64.2	32.1	2.1
**Routine**	34.1	18.7	3
**Placebo**	37.5	6.6	2.9

Figure S6.5：Cumulative ranking curve plots of gut microbiota-targeted therapies for anxiety scores. A larger surface under the cumulative ranking curve (SUCRA) reflects a higher probability of being positioned as a more effective treatment for reducing anxiety scores.

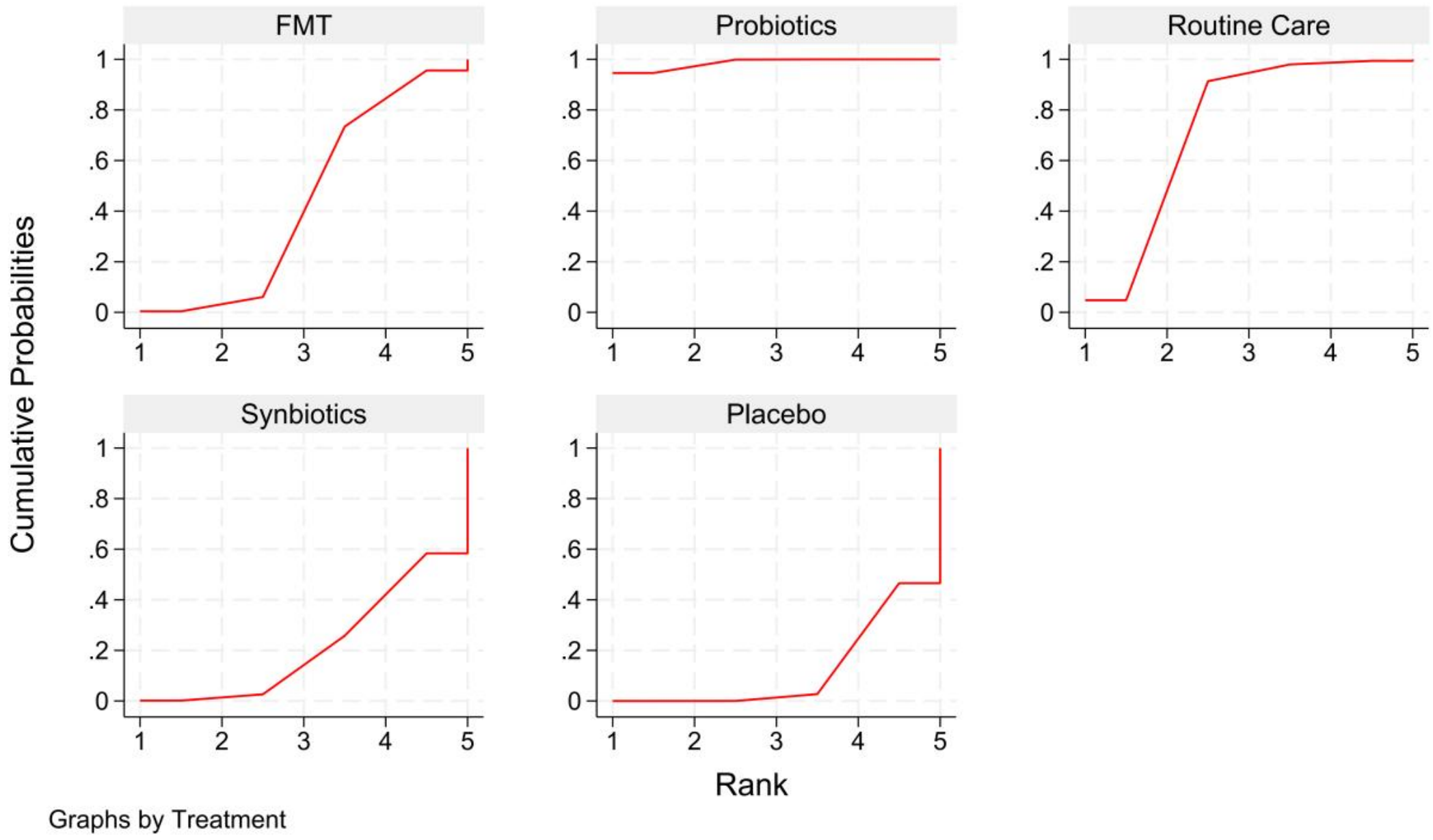

Table S6.5：SUCRA values and probability rankings of various gut microbiota-targeted therapies for anxiety scores.

Treatment	SUCRA	PrBest	MeanRank
**FMT**	43.9	0.4	3.2
**Probiotics**	98.6	94.6	1.1
**Routine Care**	73.4	4.8	2.1
**Synbiotics**	21.8	0.2	4.1
**Placebo**	12.3	0	4.5

Figure S6.6：Cumulative ranking curve plots of gut microbiota-targeted therapies for depression scores. A larger surface under the cumulative ranking curve (SUCRA) reflects a higher probability of being positioned as a more effective treatment for reducing depression scores.

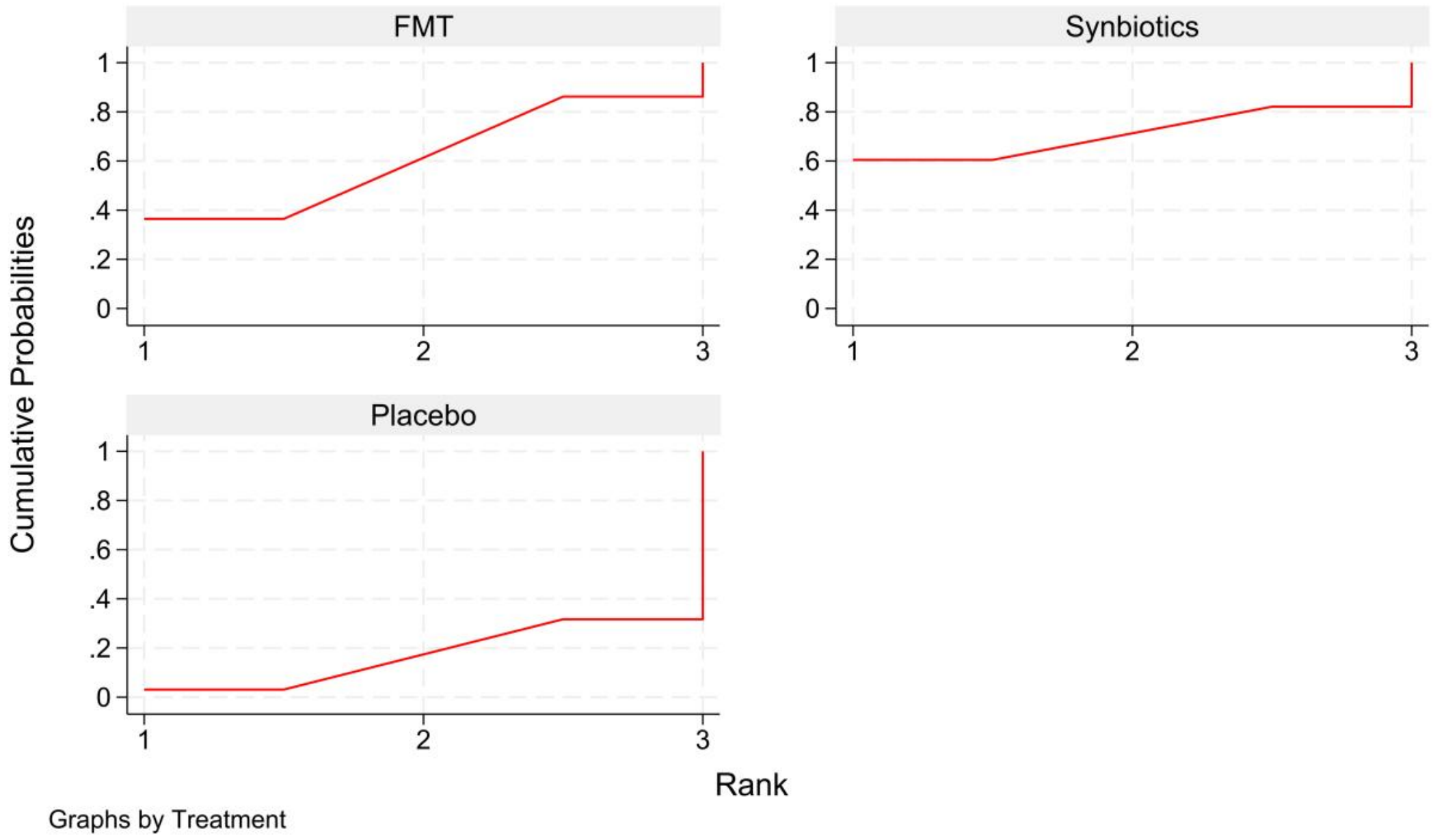

Table S6.6：SUCRA values and probability rankings of various gut microbiota-targeted therapies for depression scores.

Treatment	SUCRA	PrBest	MeanRank
**FMT**	61.3	36.5	1.8
**Synbiotics**	71.3	60.4	1.6
**Placebo**	17.4	3.1	2.7

Figure S6.7: Cumulative ranking curve plots of gut microbiota-targeted therapies for quality of life scores. A larger surface under the cumulative ranking curve (SUCRA) reflects a higher probability of being positioned as a more effective treatment for reducing quality of life scores.

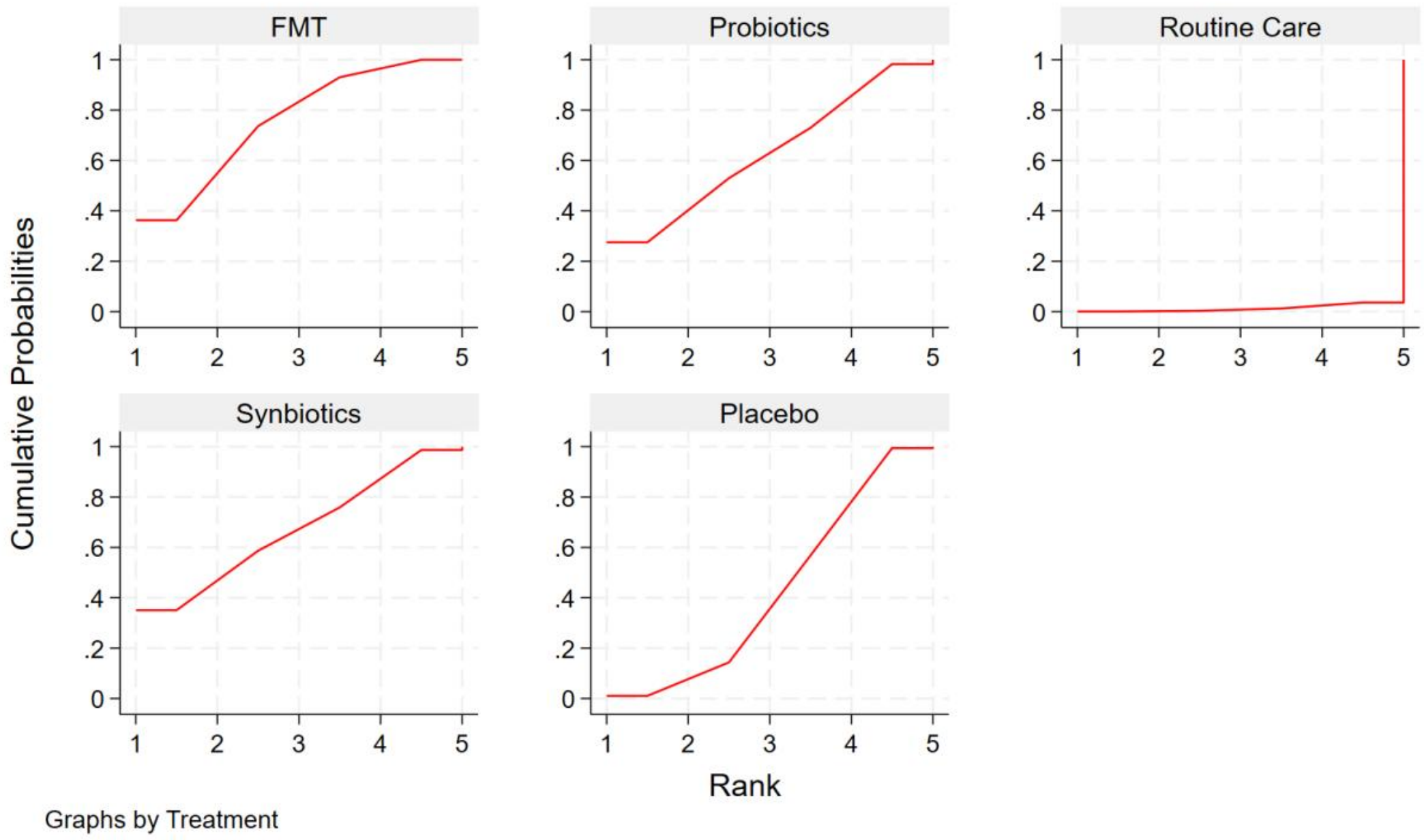

Table S6.7: SUCRA values and probability rankings of various gut microbiota-targeted therapies for quality of life scores.

Treatment	SUCRA	PrBest	MeanRank
**FMT**	75.8	36.3	2
**Probiotics**	62.9	27.5	2.5
**Routine Care**	1.3	0	4.9
**Synbiotics**	67.1	35.1	2.3
**Placebo**	42.9	1.1	3.3

Figure S6.8：Cumulative ranking curve plots of gut microbiota-targeted therapies for sleep scores. A larger surface under the cumulative ranking curve (SUCRA) reflects a higher probability of being positioned as a more effective treatment for reducing sleep scores.

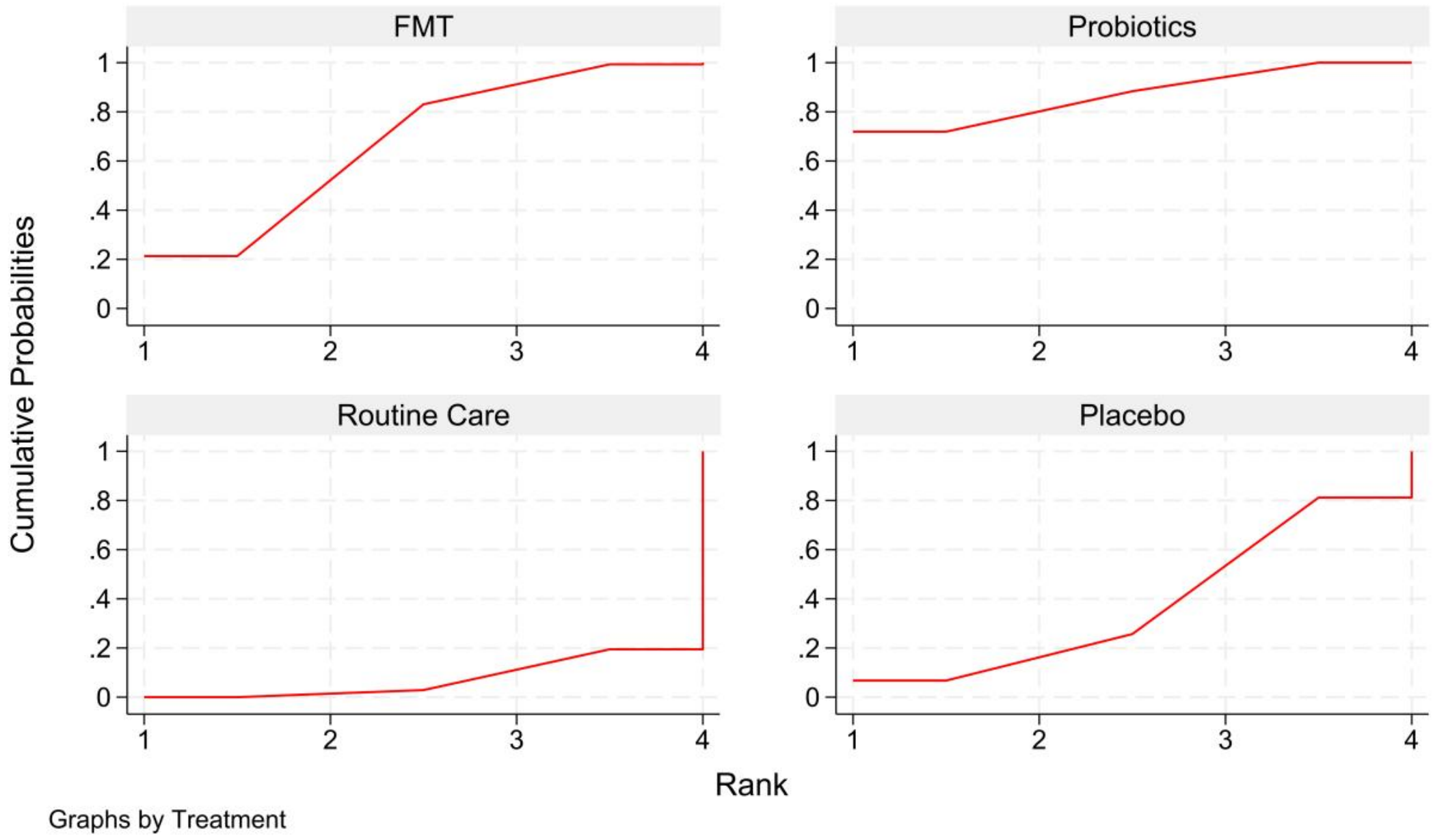

Table S6.8：SUCRA and cumulative ranking metrics of gut microbiota-targeted therapies for sleep scores.

**Treatment**	**SUCRA**	**PrBest**	**MeanRank**
**FMT**	67.9	21.3	2
**Probiotics**	86.8	71.9	1.4
**Routine**	7.5	0	3.8
**Placebo**	37.9	6.8	2.9

Figure S6.9：Cumulative ranking curve plots of gut microbiota-targeted therapies for cognitive function scores. A larger surface under the cumulative ranking curve (SUCRA) reflects a higher probability of being positioned as a more effective treatment for improving cognitive function scores.

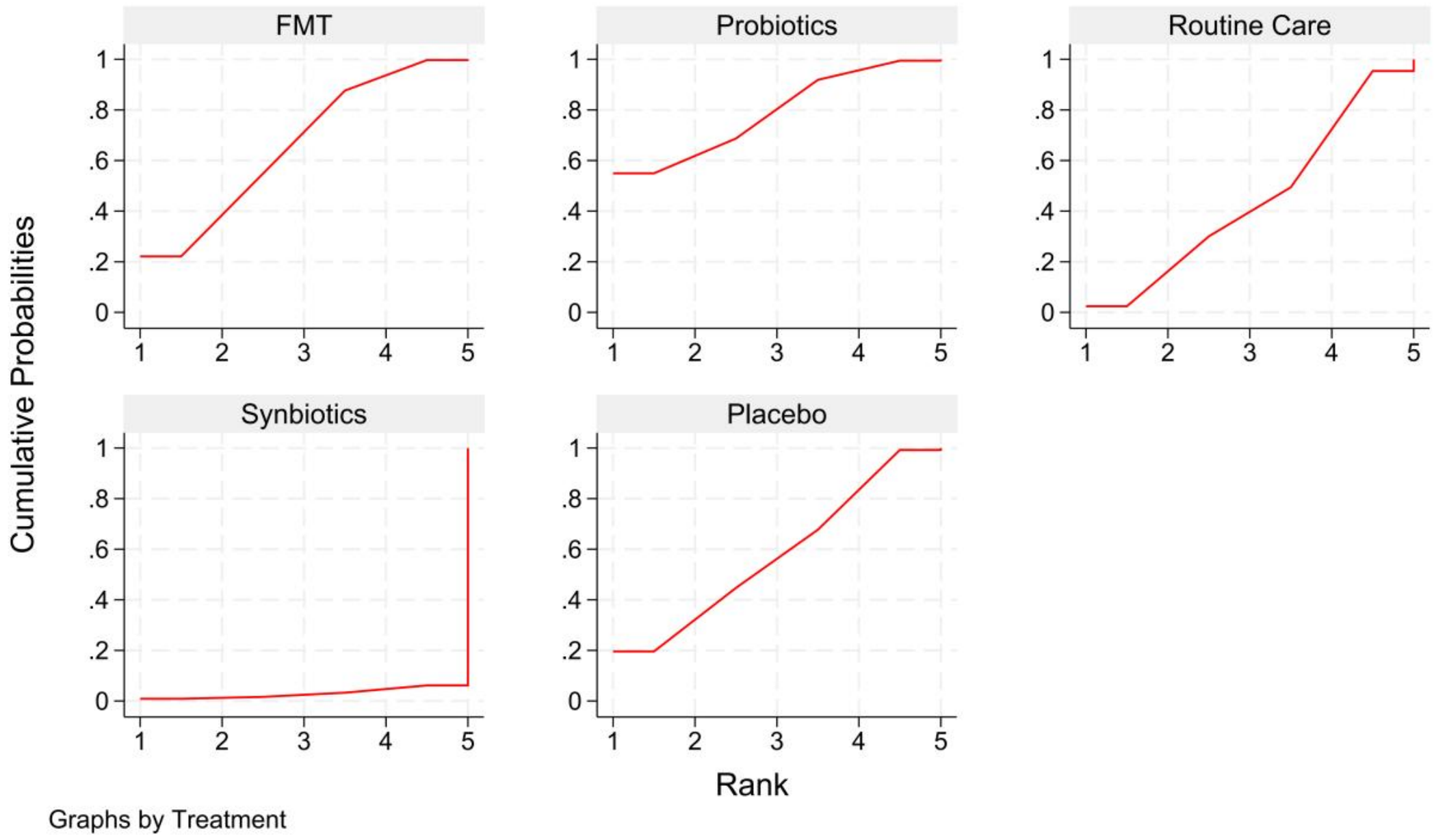

Table S6.9：SUCRA values and probability rankings of various gut microbiota-targeted therapies for cognitive function scores.

Treatment	SUCRA	PrBest	MeanRank
**FMT**	66.1	22.1	2.4
**Probiotics**	78.8	55	1.8
**Routine Care**	44.3	2.4	3.2
**Synbiotics**	3	0.9	4.9
**Placebo**	57.8	19.6	2.7

Figure S6.10： Cumulative ranking curve plots of gut microbiota-targeted therapies for TAC. A larger surface under the cumulative ranking curve (SUCRA) reflects a higher probability of being positioned as a more effective treatment for increasing total antioxidant capacity (TAC) levels.

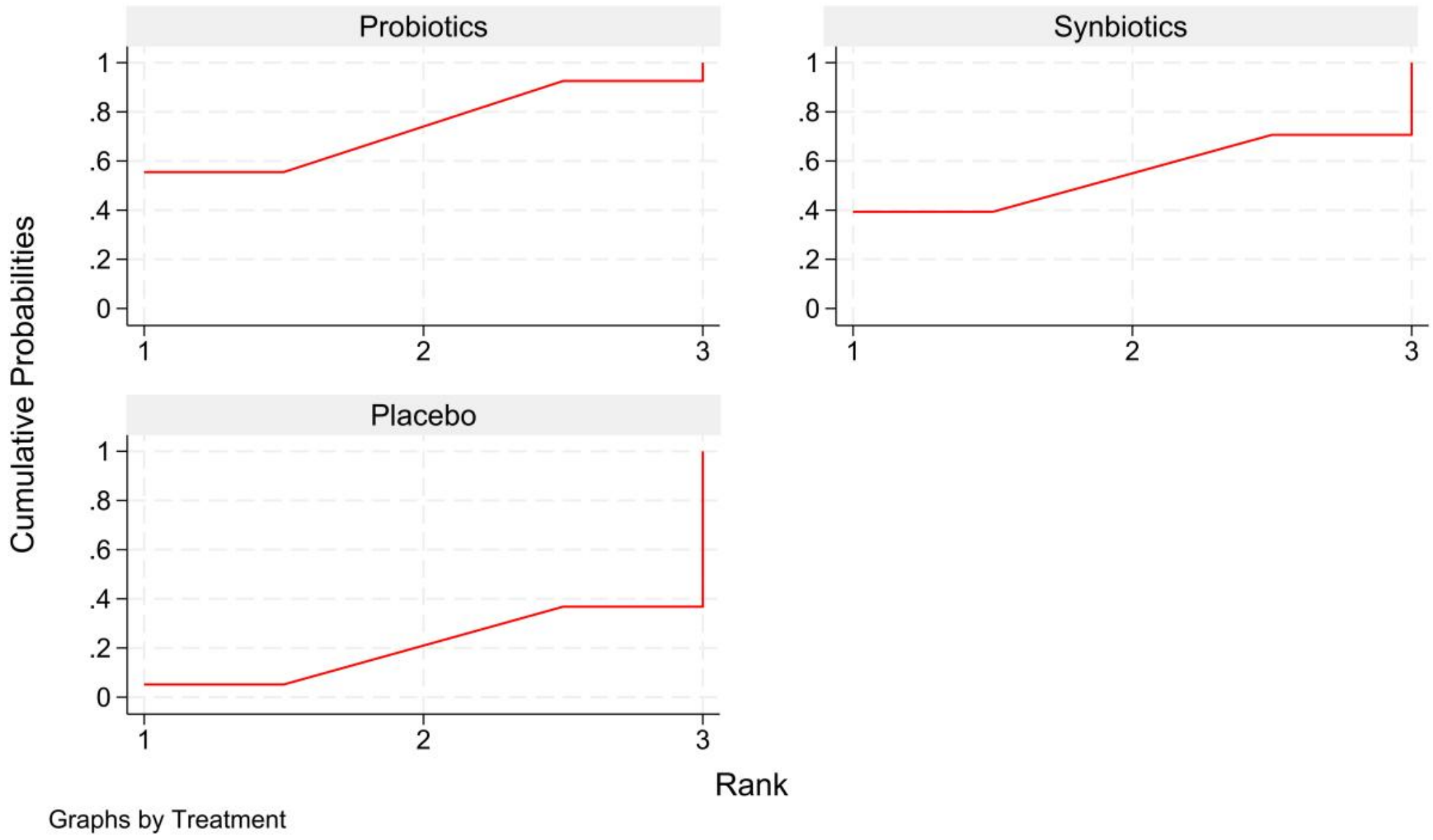

Table S6.10：SUCRA values and probability rankings of various gut microbiota-targeted therapies for TAC.

Treatment	SUCRA	PrBest	MeanRank
**Probiotics**	74	55.5	1.5
**Synbiotics**	55	39.3	1.9
**Placebo**	21	5.2	2.6

Figure S6.11：Cumulative ranking curve plots of gut microbiota-targeted therapies for MDA. A larger surface under the cumulative ranking curve (SUCRA) reflects a higher probability of being positioned as a more effective treatment for reducing malondialdehyde (MDA) levels.

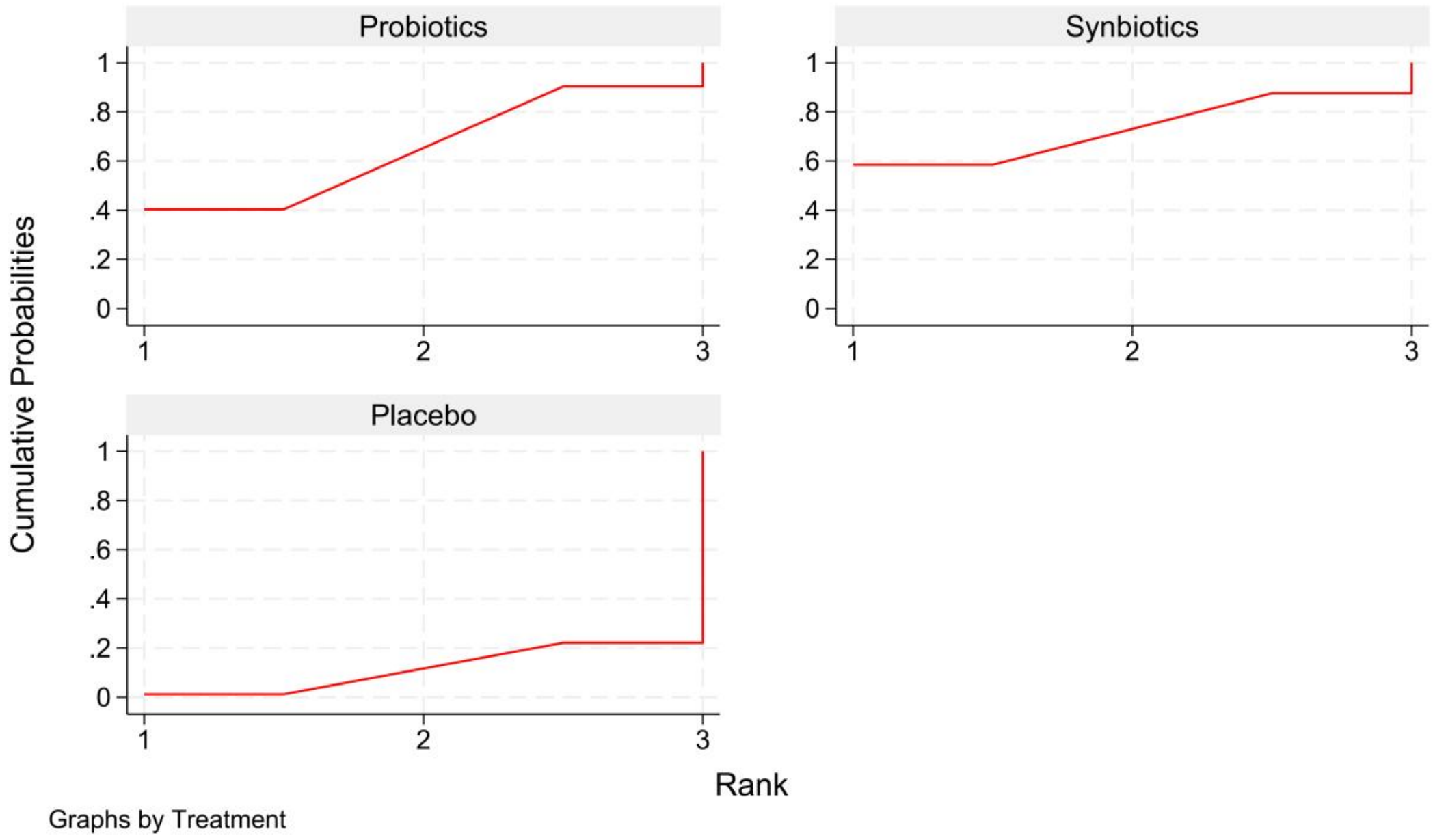

Table S6.11：SUCRA values and probability rankings of various gut microbiota-targeted therapies for MDA.

Treatment	SUCRA	PrBest	MeanRank
**Probiotics**	65.3	40.3	1.7
**Synbiotics**	73	58.5	1.5
**Placebo**	11.7	1.2	2.8

Appendix 7：Summary Evaluation Scale for the Efficacy of Targeted Intestinal Microbiota Therapies in Parkinson's Disease Based on a Network Meta-analysis of 19 Trials

Table S7.1：Network meta-analysis league table for UPDRS scores.

Data are presented as mean differences (MDs) with accompanied 95% confidence intervals (95% CIs). Each cell represents the comparative estimate of the row intervention relative to the column intervention. For negative outcomes such as UPDRS, an MD < 0 favors the row intervention, whereas an MD > 0 favors the column intervention. For instance, the MD in UPDRS scores for FMT compared with Probiotics was -9.98 (95% CI: -22.03 to 2.06). Statistically significant results are highlighted in bold (where the 95% CI excludes 0).

FMT		
-9.98 (-22.03,2.06)	Probiotics	
-15.32 (-24.63,-6.02)	-5.34 (-13.03,2.35)	Placebo

Table S7.2：Network meta-analysis league table for MDS-UPDRS III scores.

Data are presented as mean differences (MDs) with accompanied 95% confidence intervals (95% CIs). Each cell represents the comparative estimate of the row intervention relative to the column intervention. For negative outcomes such as MDS-UPDRS III, an MD < 0 favors the row intervention, whereas an MD > 0 favors the column intervention. For instance, the MD in MDS-UPDRS III scores for FMT compared with Probiotics was -1.92 (95% CI: -5.46 to 1.62). Statistically significant results are highlighted in bold (where the 95% CI excludes 0).

FMT				
-1.92 (-5.46,1.62)	Probiotics			
-3.81 (-7.72,0.09)	-1.90 (-4.26,0.47)	Routine Care		
0.61 (-4.42,5.63)	2.52 (-2.83,7.87)	4.42 (-1.29,10.13)	Synbiotics	
-2.46 (-4.97,0.05)	-0.55 (-3.58,2.49)	1.35 (-2.14,4.85)	-3.07 (-7.52,1.38)	Placebo

Table S7.3：Network meta-analysis league table for SBM frequency.

Data are presented as mean differences (MDs) with accompanied 95% confidence intervals (95% CIs). Each cell represents the comparative estimate of the row intervention relative to the column intervention. For positive outcomes such as SBM frequency, an MD > 0 favors the row intervention, whereas an MD < 0 favors the column intervention. For instance, the MD in SBM frequency for Probiotics compared with Placebo was 1.93 (95% CI: 0.23 to 3.64). Statistically significant results are highlighted in bold.

FMT				
-1.51 (-4.20,1.17)	Probiotics			
1.71 (-0.55,3.97)	3.22 (-0.28,6.73)	Routine Care		
-0.81 (-3.43,1.80)	0.70 (-1.64,3.04)	-2.52 (-5.98,0.93)	Synbiotics	
0.42 (-1.65,2.49)	1.93 (0.23,3.64)	-1.29 (-4.35,1.77)	1.23 (-0.37,2.84)	Placebo

Table S7.4：Network meta-analysis league table for gastrointestinal symptoms.

Data are presented as mean differences (MDs) with accompanied 95% confidence intervals (95% CIs). Each cell represents the comparative estimate of the row intervention relative to the column intervention. For negative outcomes such as gastrointestinal symptoms, an MD < 0 favors the row intervention, whereas an MD > 0 favors the column intervention. For instance, the MD in gastrointestinal symptom scores for Probiotics compared with Placebo was -2.74 (95% CI: -12.11 to 6.63). Statistically significant results are highlighted in bold.

FMT			
-0.52 (-13.95,12.91)	Probiotics		
-4.57 (-23.41,14.27)	-4.05 (-17.29,9.20)	Routine Care	
-3.26 (-12.93,6.40)	-2.74 (-12.11,6.63)	1.31 (-14.90,17.52)	Placebo

Table S7.5：Network meta-analysis league table for anxiety scores.

Data are presented as mean differences (MDs) with accompanied 95% confidence intervals (95% CIs). Each cell represents the comparative estimate of the row intervention relative to the column intervention. For negative outcomes such as anxiety scores, an MD < 0 favors the row intervention, whereas an MD > 0 favors the column intervention. For instance, the MD in anxiety scores for Probiotics compared with Placebo was -11.33 (95% CI: -16.77 to -5.89). Statistically significant results are highlighted in bold.

FMT				
8.81 (2.50,15.12)	Probiotics			
5.61 (-1.72,12.95)	-3.20 (-6.97,0.58)	Routine Care		
-2.04 (-8.09,4.01)	-10.85 (-18.33,-3.37)	-7.65 (-16.03,0.73)	Synbiotics	
-2.52 (-5.72,0.68)	-11.33 (-16.77,-5.89)	-8.13 (-14.76,-1.51)	-0.48 (-5.62,4.65)	Placebo

Table S7.6：Network meta-analysis league table for depression scores.

Data are presented as mean differences (MDs) with accompanied 95% confidence intervals (95% CIs). Each cell represents the comparative estimate of the row intervention relative to the column intervention. For negative outcomes such as depression scores, an MD < 0 favors the row intervention, whereas an MD > 0 favors the column intervention. For instance, the MD in depression scores for Synbiotics compared with Placebo was -2.07 (95% CI: -6.61 to 2.46). Statistically significant results are highlighted in bold.

FMT		
0.78 (-4.40,5.96)	Synbiotics	
-1.30 (-3.80,1.21)	-2.07 (-6.61,2.46)	Placebo

Table S7.7：Network meta-analysis league table for quality of life scores.

Data are presented as mean differences (MDs) with accompanied 95% confidence intervals (95% CIs). Each cell represents the comparative estimate of the row intervention relative to the column intervention. For negative outcomes such as quality of life scores (where lower scores indicate improvement), an MD < 0 favors the row intervention, whereas an MD > 0 favors the column intervention. For instance, the MD in quality of life scores for FMT compared with Routine Care was -12.85 (95% CI: -21.38 to -4.32). Statistically significant results are highlighted in bold.

FMT				
-0.91 (-8.29,6.47)	Probiotics			
-12.85 (-21.38,-4.32)	-11.94 (-23.22,-0.67)	Routine Care		
-0.46 (-8.42,7.50)	0.45 (-8.92,9.82)	12.39 (0.73,24.06)	Synbiotics	
-2.42 (-6.28,1.44)	-1.51 (-7.77,4.75)	10.43 (1.07,19.79)	-1.96 (-8.92,5.01)	Placebo

Table S7.8：Network meta-analysis league table for sleep scores.

Data are presented as mean differences (MDs) with accompanied 95% confidence intervals (95% CIs). Each cell represents the comparative estimate of the row intervention relative to the column intervention. For negative outcomes such as sleep scores (where lower scores indicate better sleep quality), an MD < 0 favors the row intervention, whereas an MD > 0 favors the column intervention. For instance, the MD in sleep scores for Probiotics compared with Routine Care was -2.52 (95% CI: -3.86 to -1.18). Statistically significant results are highlighted in bold.

FMT			
0.75 (-1.50,2.99)	Probiotics		
-1.78 (-3.65,0.10)	-2.52 (-3.86,-1.18)	Routine Care	
-0.71 (-2.25,0.83)	-1.45 (-4.08,1.17)	1.07 (-1.28,3.42)	Placebo

Table S7.9: Network meta-analysis league table for cognitive function scores.

Data are presented as mean differences (MDs) with accompanied 95% confidence intervals (95% CIs). Each cell represents the comparative estimate of the row intervention relative to the column intervention. For positive clinical outcomes such as cognitive function scores (where higher scores indicate better cognitive performance), an MD > 0 favors the row intervention, whereas an MD < 0 favors the column intervention. For instance, the MD in cognitive function scores for FMT compared with Probiotics was -0.42 (95% CI: -3.12 to 2.28). Statistically significant results are highlighted in bold.

FMT				
-0.42 (-3.12,2.28)	Probiotics			
0.52 (-1.79,2.83)	0.94 (-0.46,2.34)	Routine Care		
4.72 (0.12,9.31)	5.14 (-0.19,10.46)	4.20 (-0.94,9.34)	Synbiotics	
0.19 (-1.08,1.45)	0.61 (-2.38,3.59)	-0.33 (-2.96,2.30)	-4.53 (-8.95,-0.11)	Placebo

Table S7.10: Network meta-analysis league table for total antioxidant capacity (TAC).

Data are presented as mean differences (MDs) with accompanied 95% confidence intervals (95% CIs). Each cell represents the comparative estimate of the row intervention relative to the column intervention. For positive biochemical markers such as TAC (where higher values indicate stronger antioxidant capacity), an MD > 0 favors the row intervention, whereas an MD < 0 favors the column intervention. For instance, the MD in TAC (μM) for Synbiotics compared with Probiotics was 68.56 (95% CI: -449.08 to 586.19). Statistically significant results are highlighted in bold.

Probiotics		
68.56 (-449.08,586.19)	Synbiotics	
201.53 (-124.57,527.63)	132.98(-374.36,640.32)	Placebo

Table S7.11：Network meta-analysis league table for malondialdehyde (MDA). Data are presented as mean differences (MDs) with accompanied 95% confidence intervals (95% CIs). Each cell represents the comparative estimate of the row intervention relative to the column intervention. For negative biochemical markers such as MDA (where lower values indicate mitigated oxidative stress), an MD < 0 favors the row intervention, whereas an MD > 0 favors the column intervention. For instance, the MD in MDA (μM) for Synbiotics compared with Probiotics was 0.54 (95% CI: -3.91 to 4.99). Statistically significant results are highlighted in bold.

Probiotics		
0.54 (-3.91,4.99)	Synbiotics	
-1.60 (-4.16,0.95)	-2.14 (-5.78,1.50)	Placebo

Appendix 8：Funnel plots

Figure S8.1：UPDRS

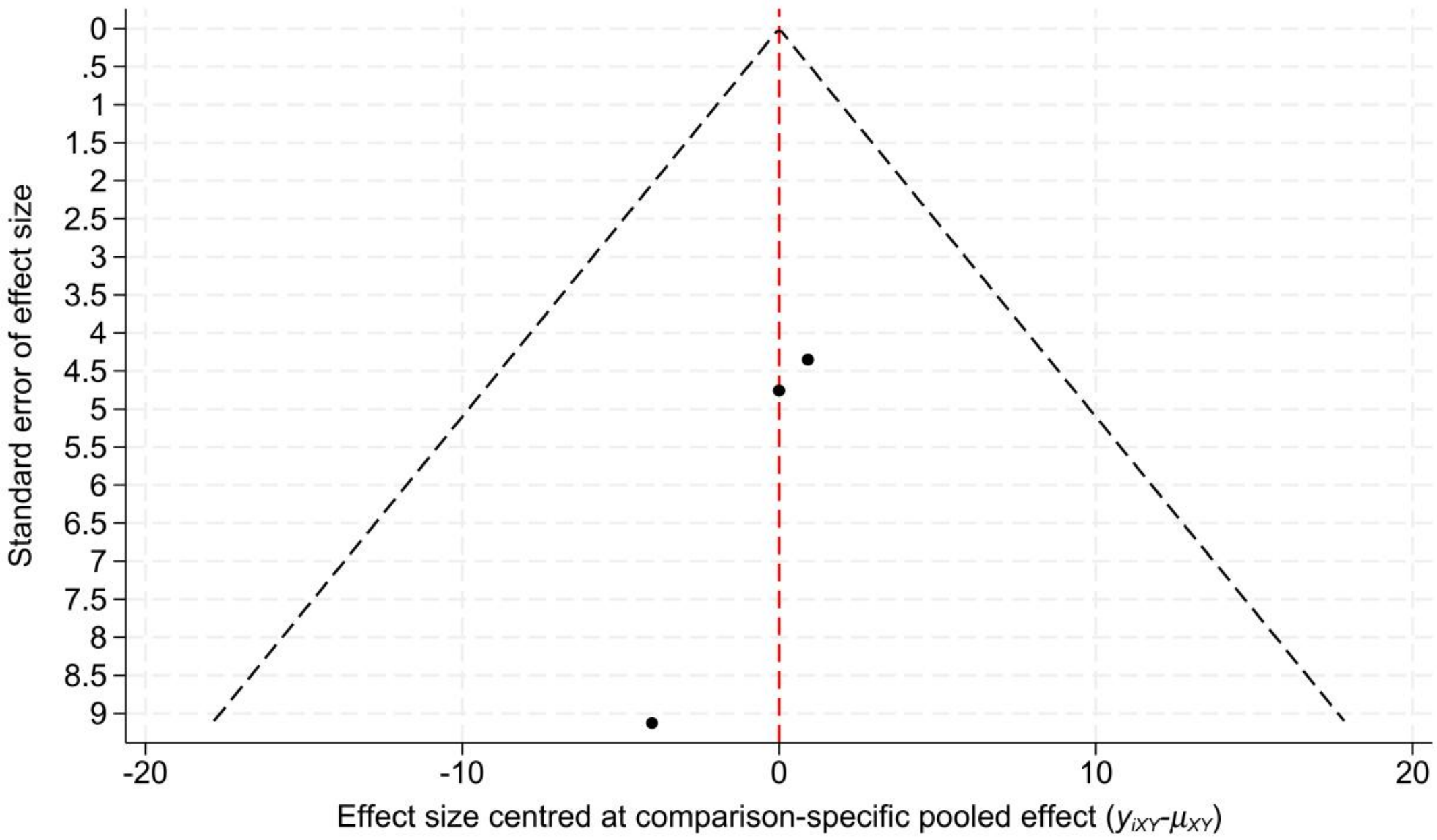

Figure S8.2：UPDRS III

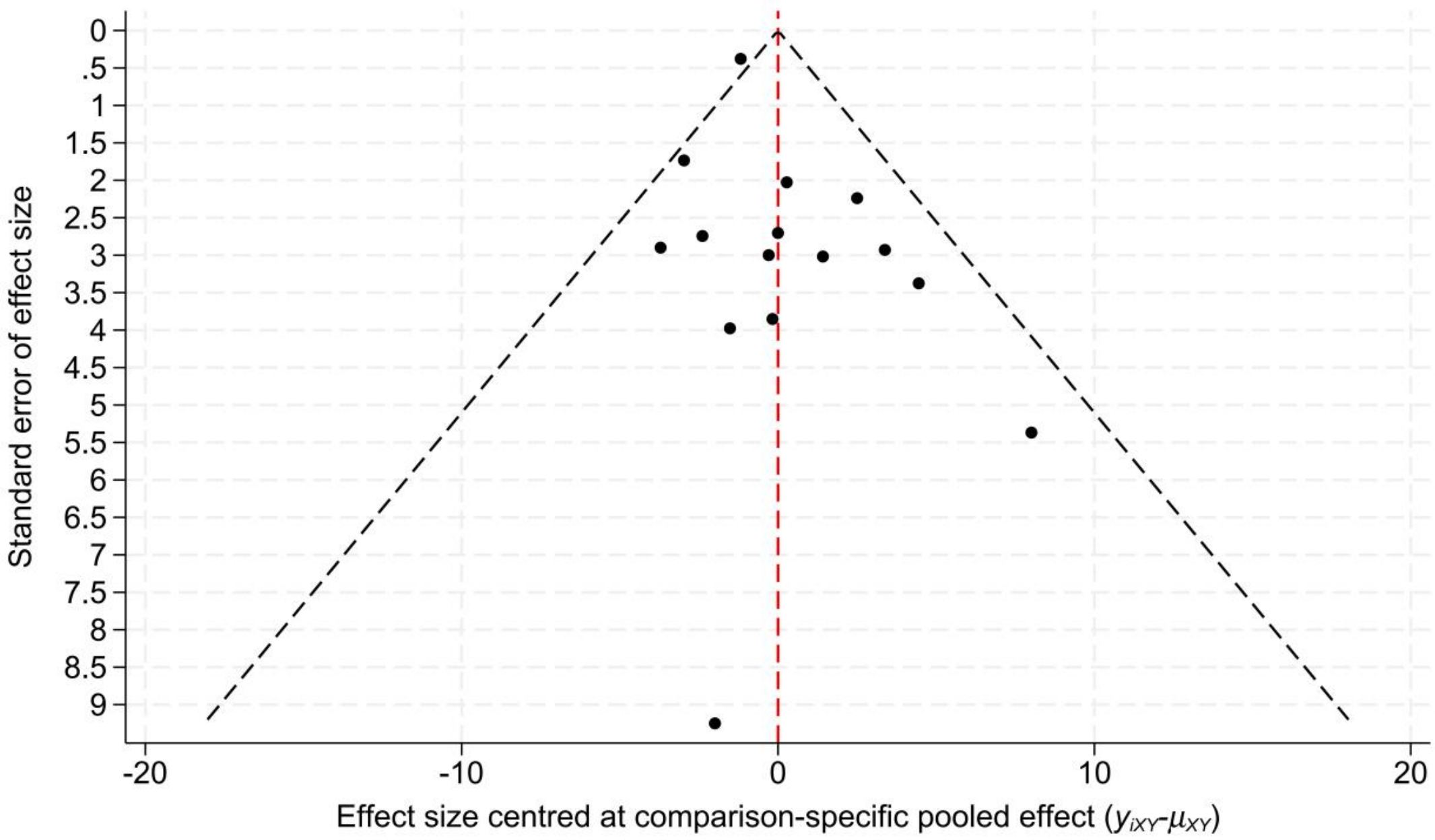

Figure S8.3: SBM

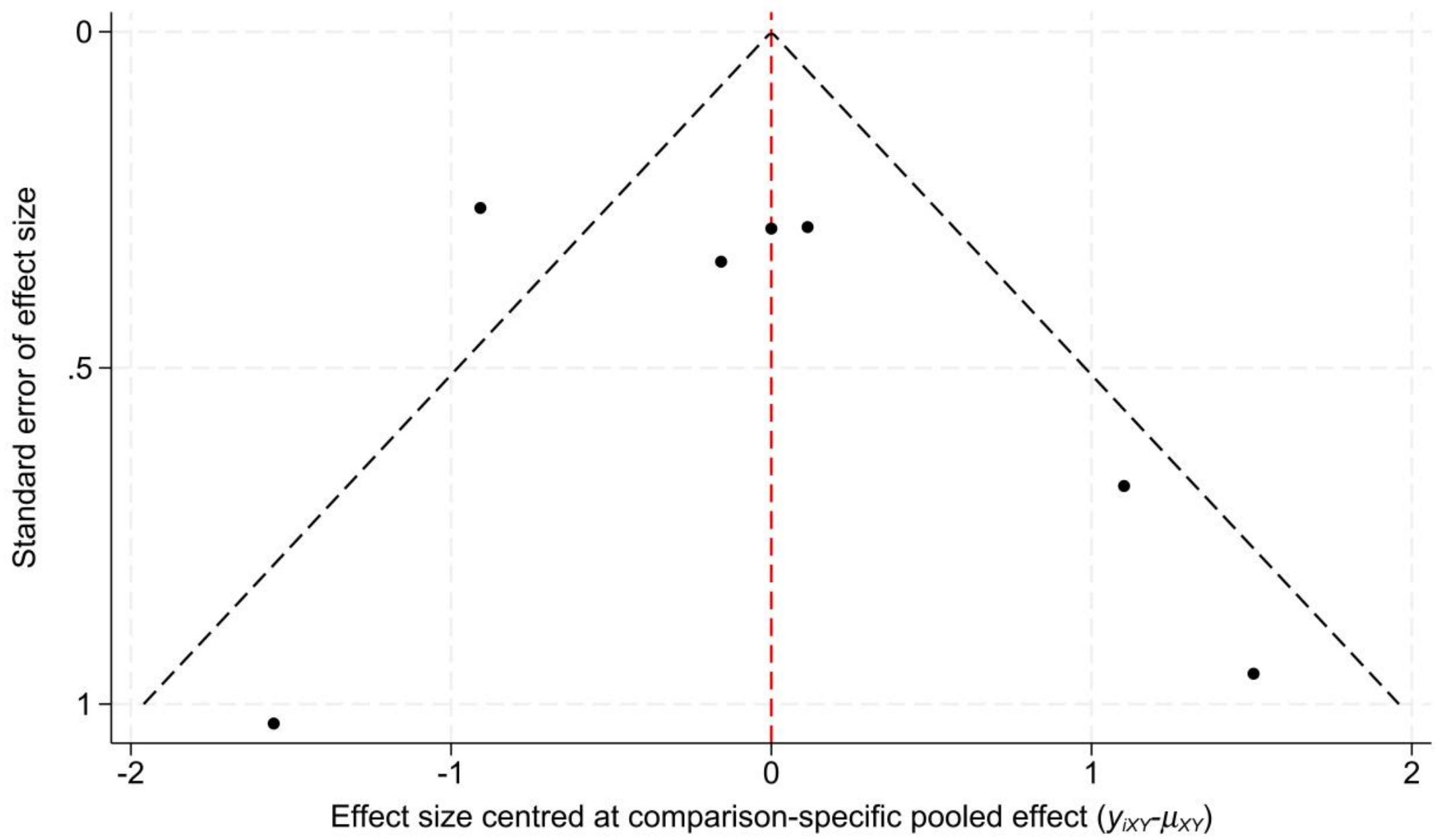

Figure S8.4: Gastrointestinal Symptom

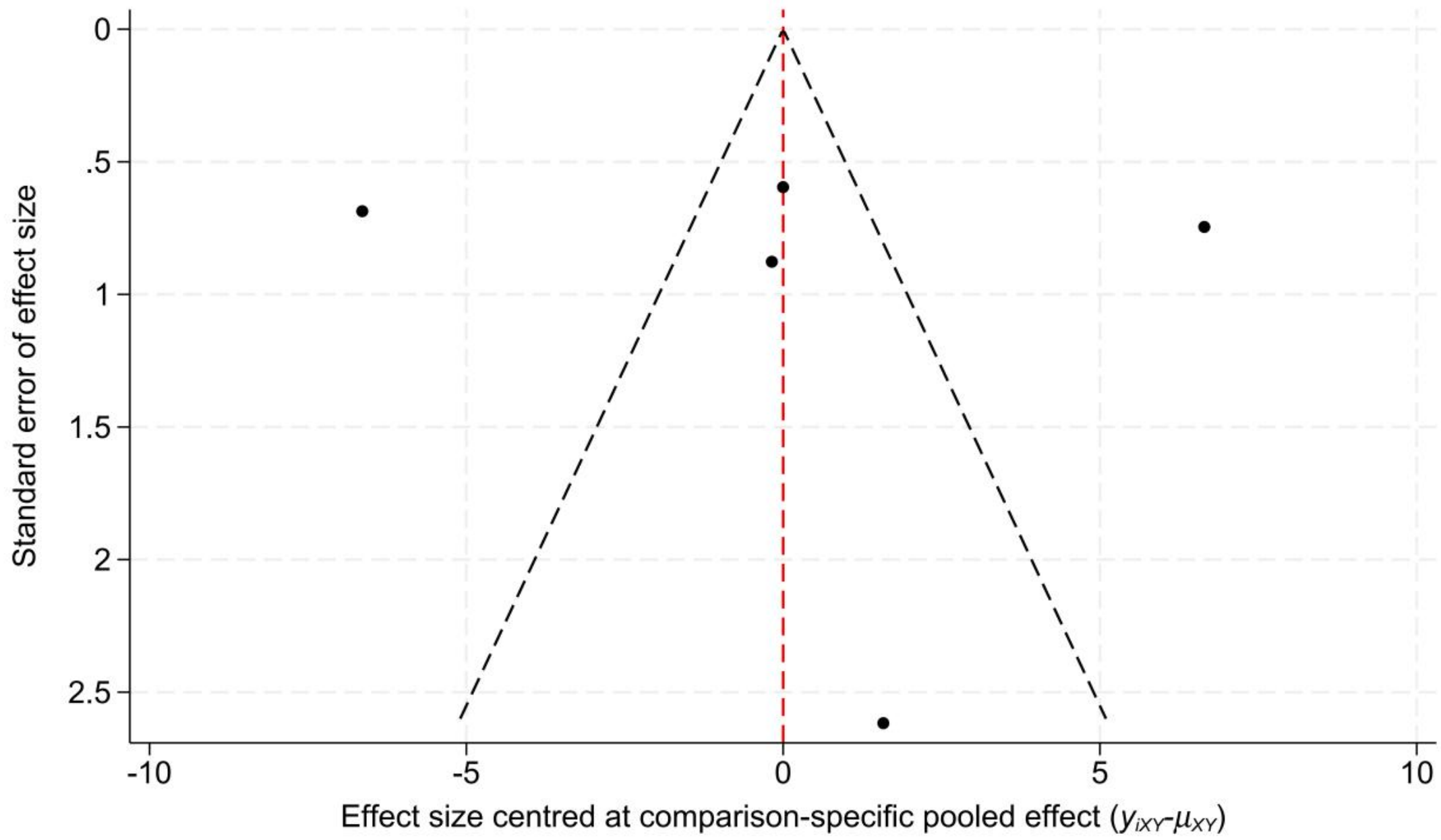

Figure S8.5: Anxiety Score

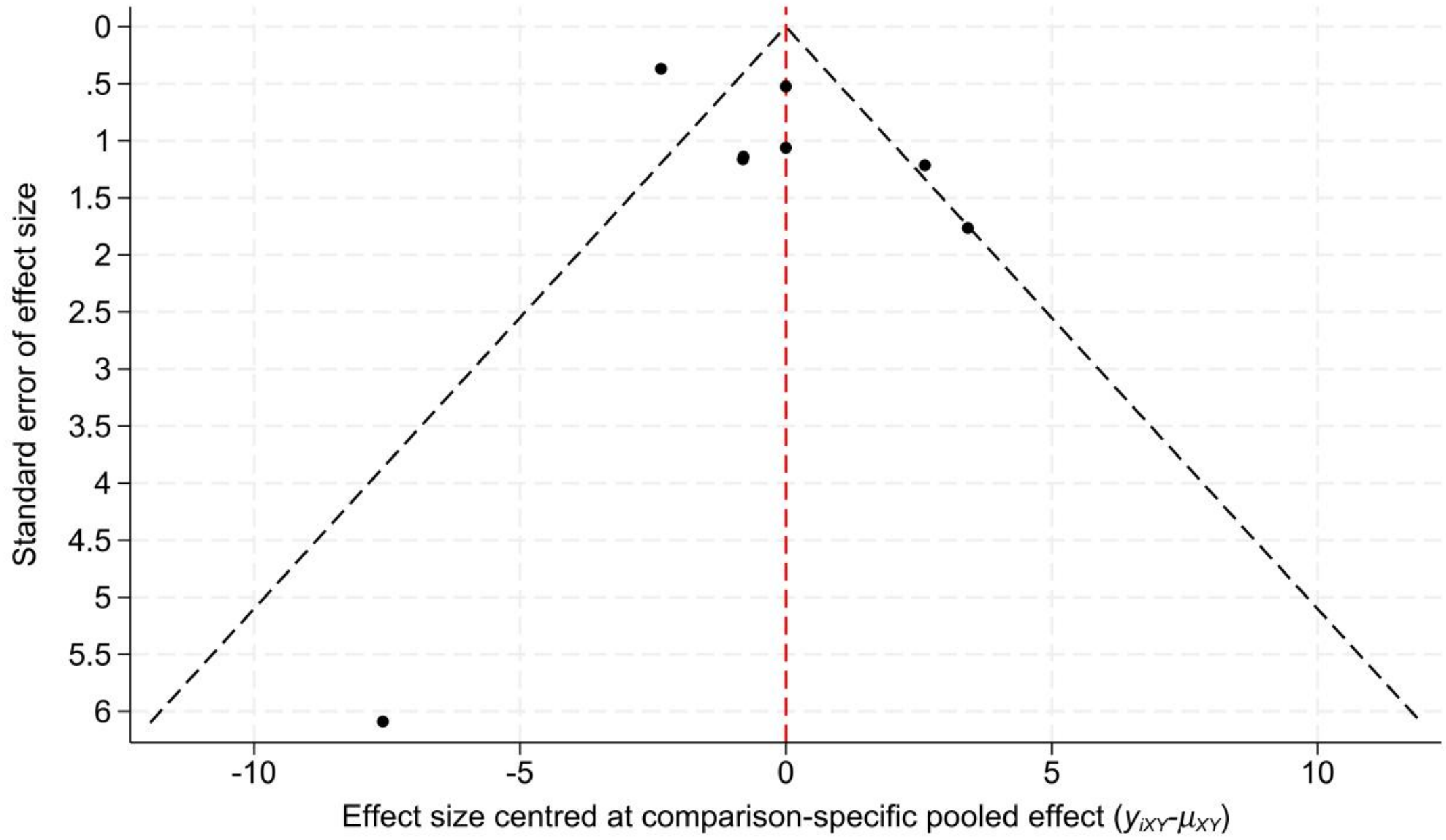

Figure S8.6: Depression Score

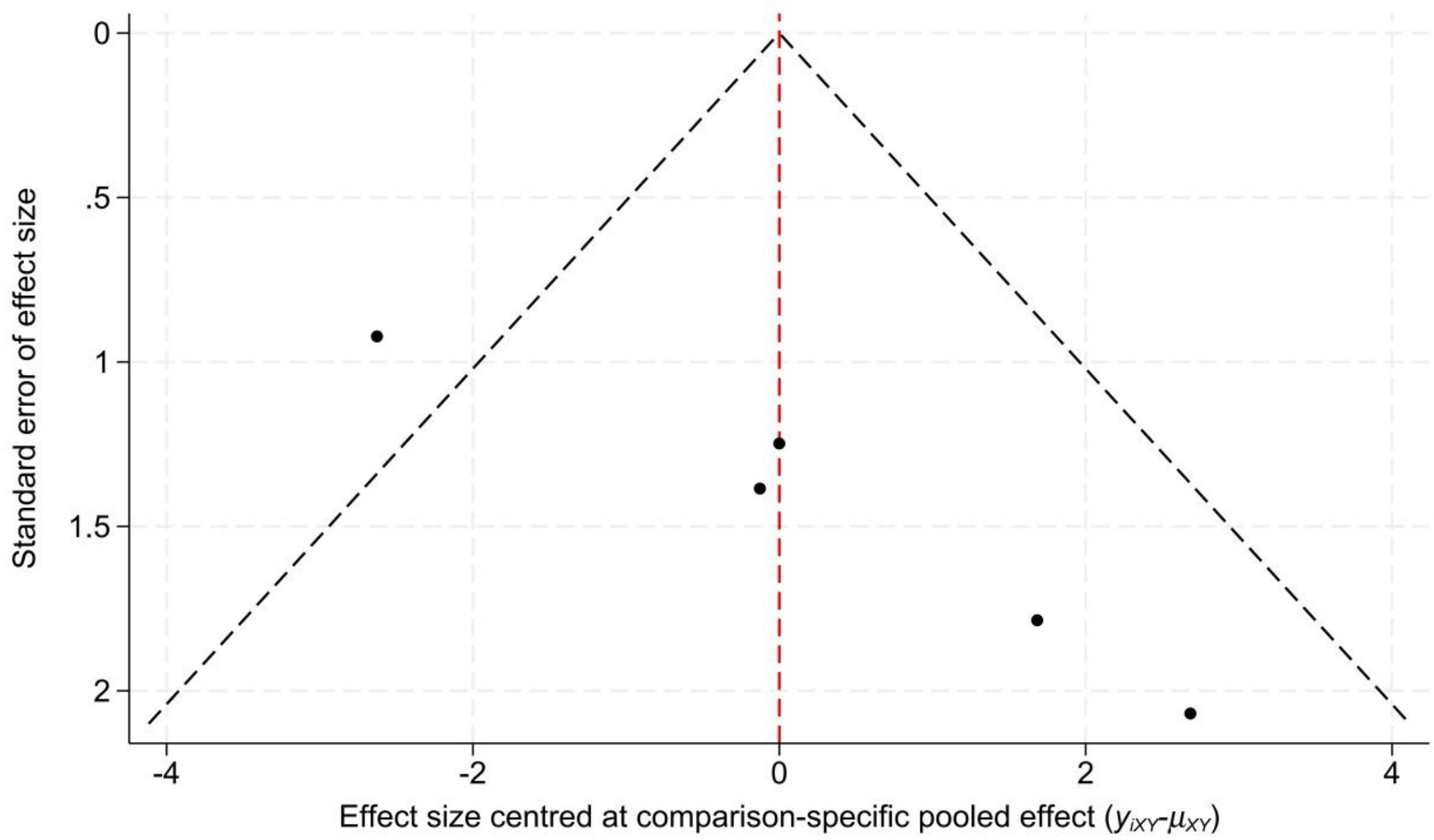

Figure S8.7: Quality of Life Score

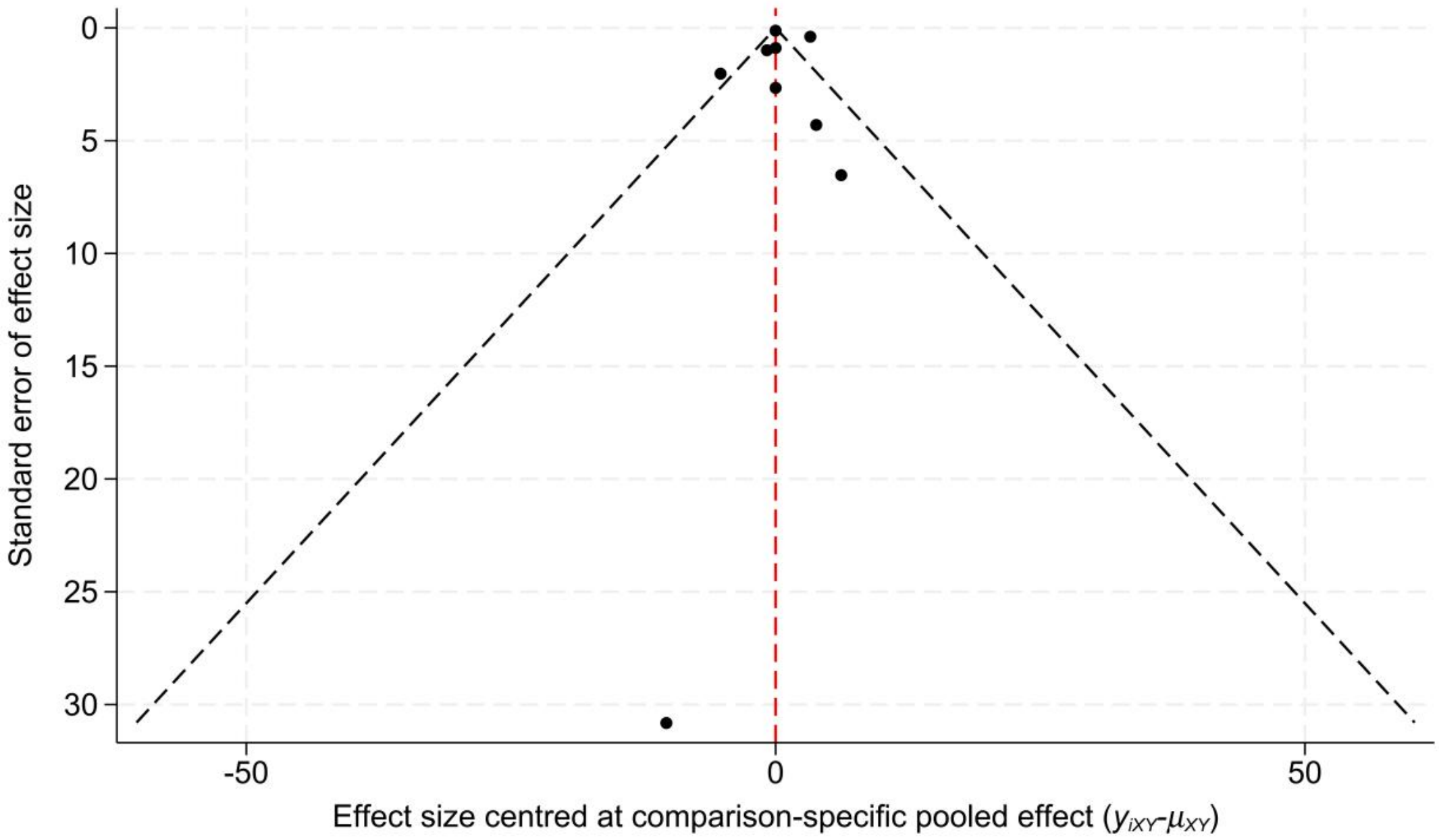

Figure S8.8: Sleep Score

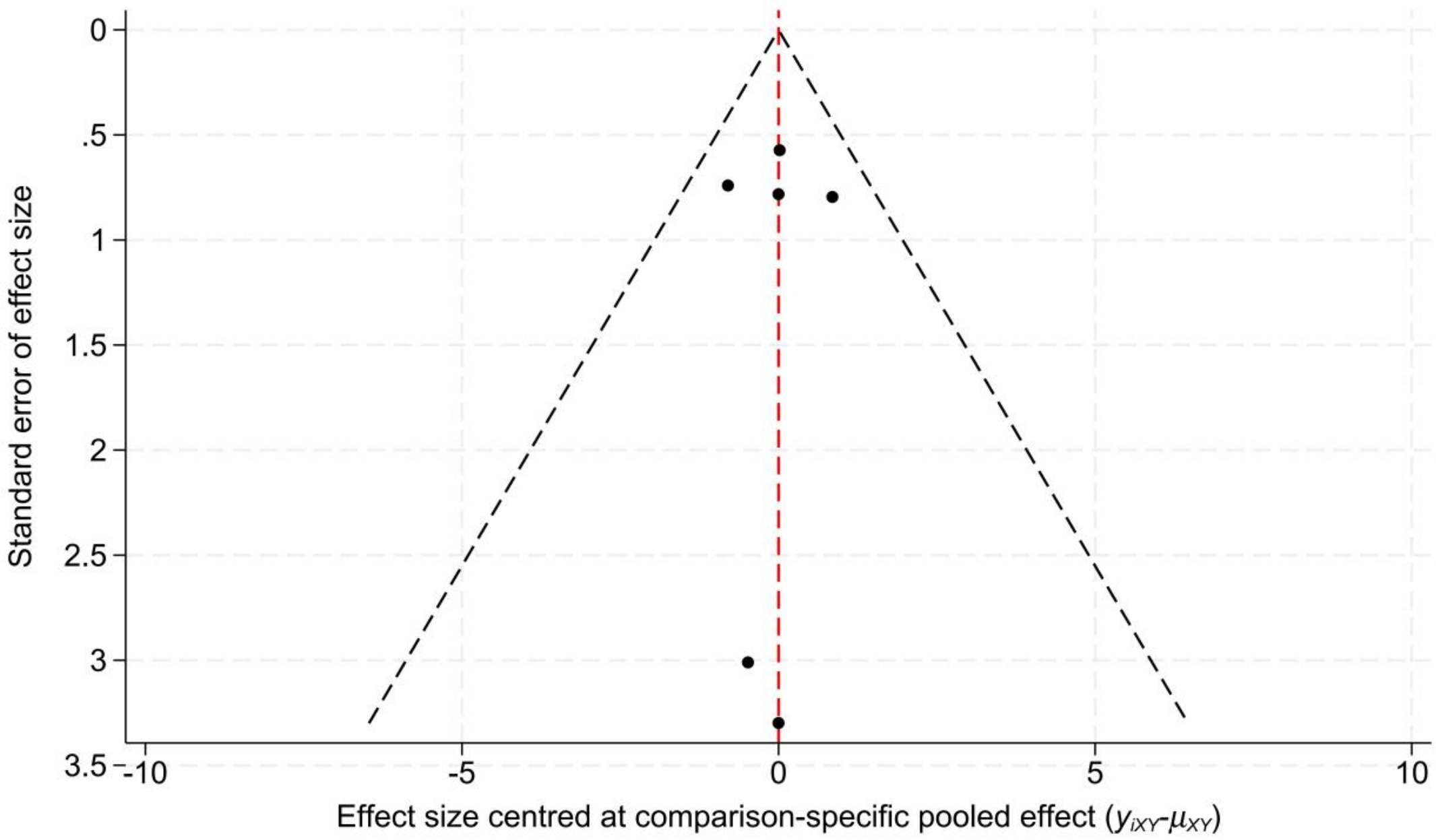

Figure S8.9: Cognitive Function Score

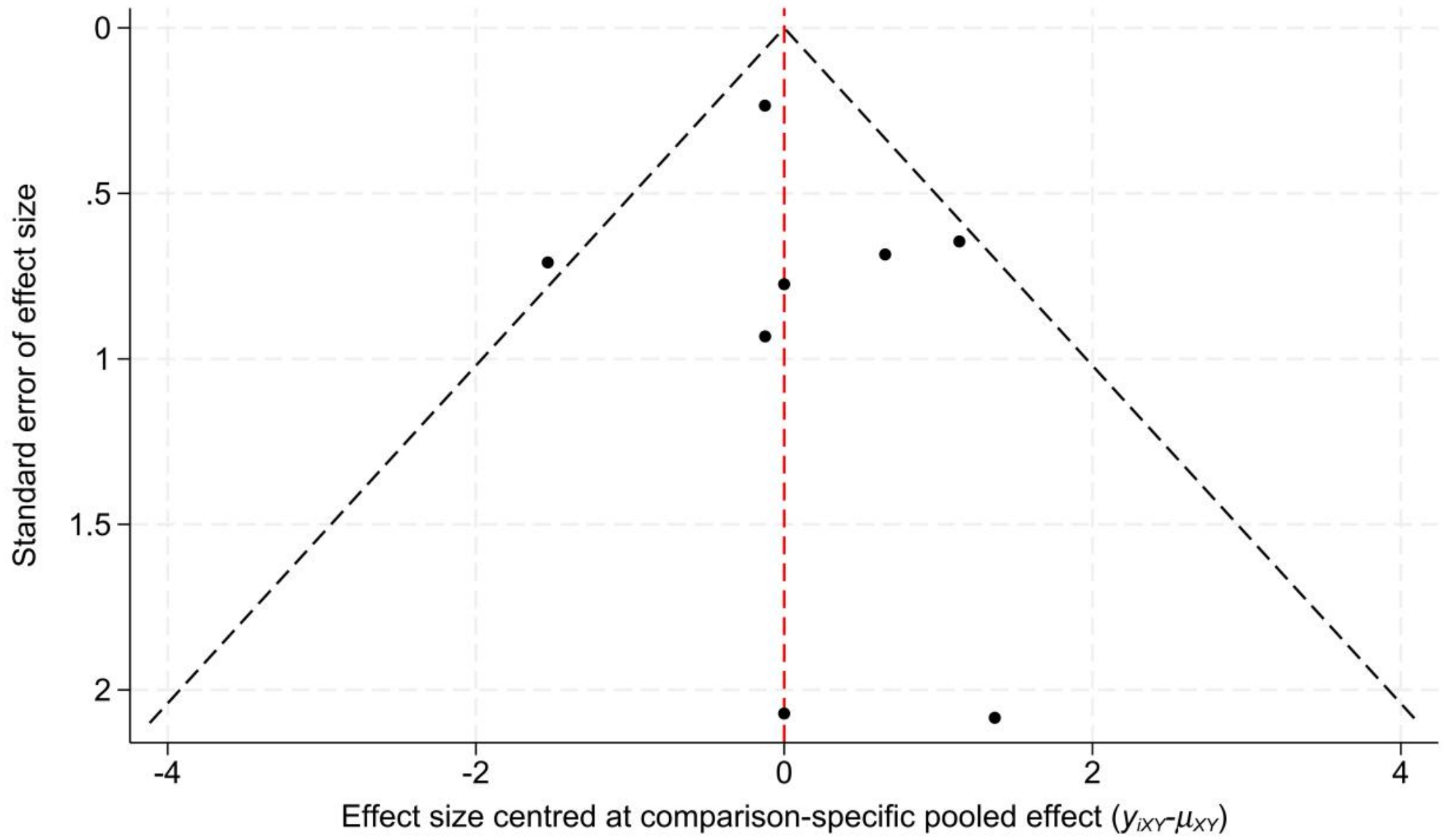

Figure S8.10: Total Antioxidant Capacity (TAC, μM)

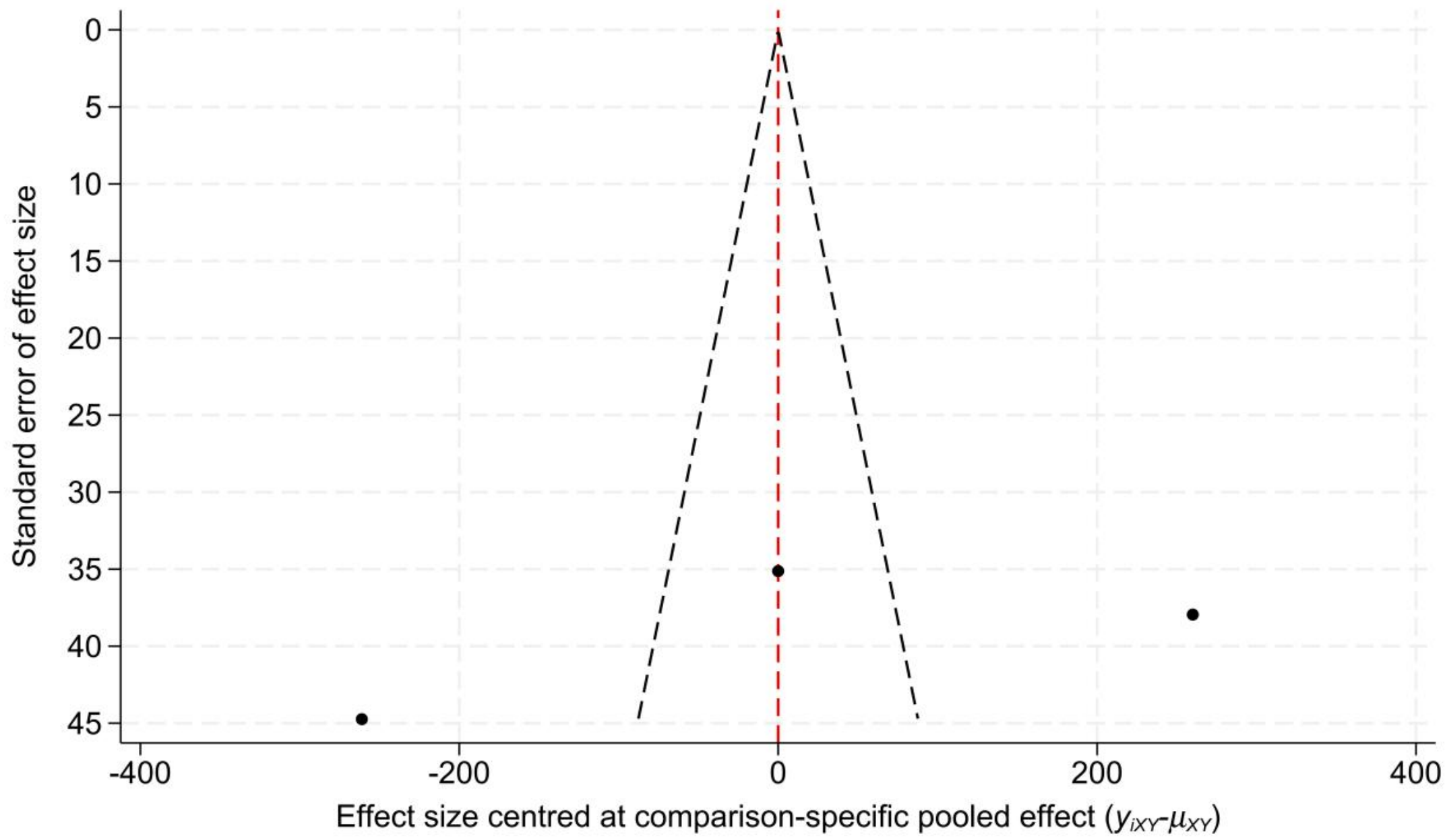

Figure S8.11: Malondialdehyde (MDA, μM)

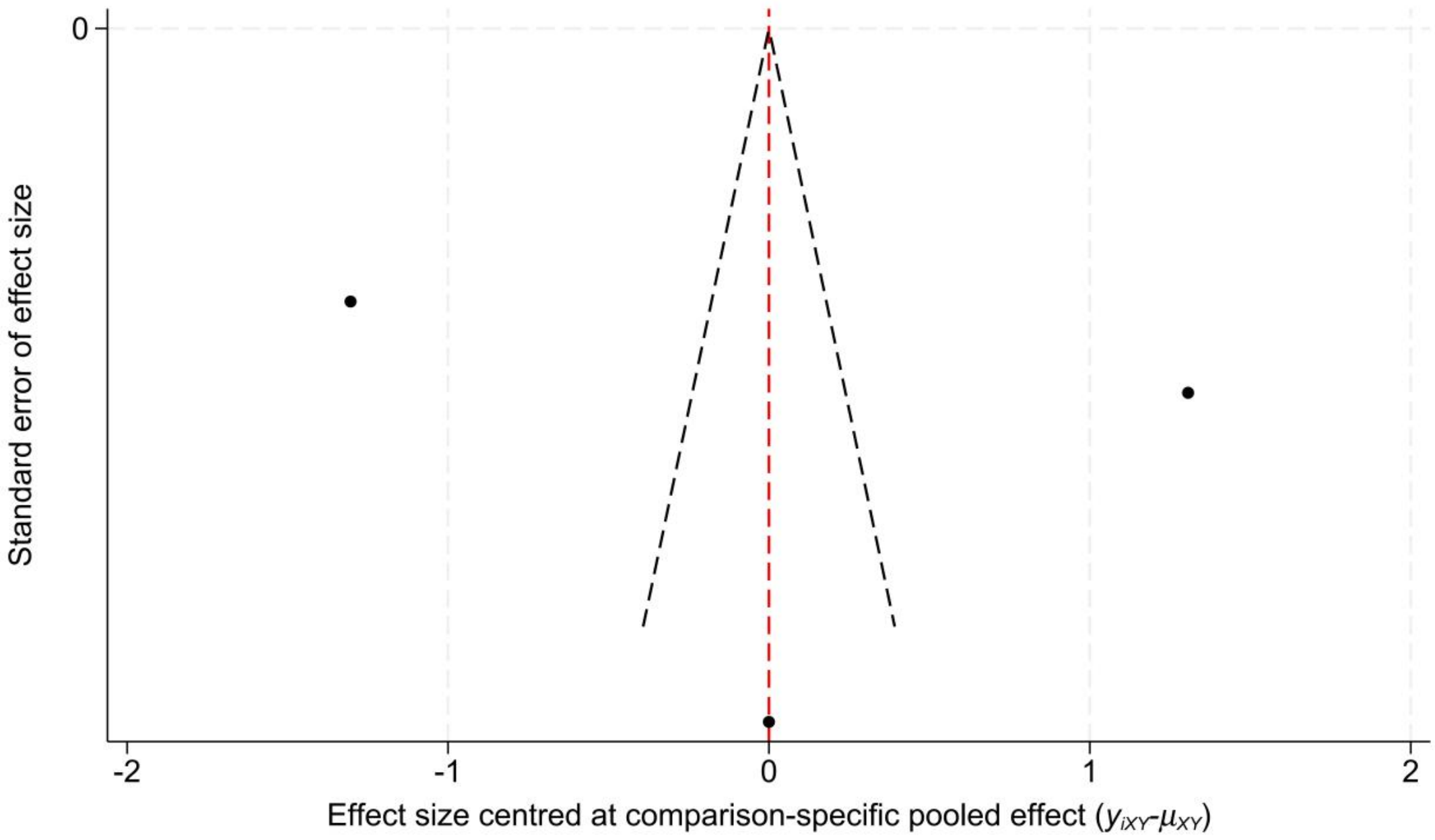

Appendix 9：Subgroup analysis by different administration routes

Appendix 9.1：Oral administration route

Figure S9.1：Cumulative ranking curve plots of gut microbiota-targeted therapies for MDS-UPDRS III scores. A larger surface under the cumulative ranking curve (SUCRA) reflects a higher probability of being positioned as a more effective treatment for reducing MDS-UPDRS III scores.

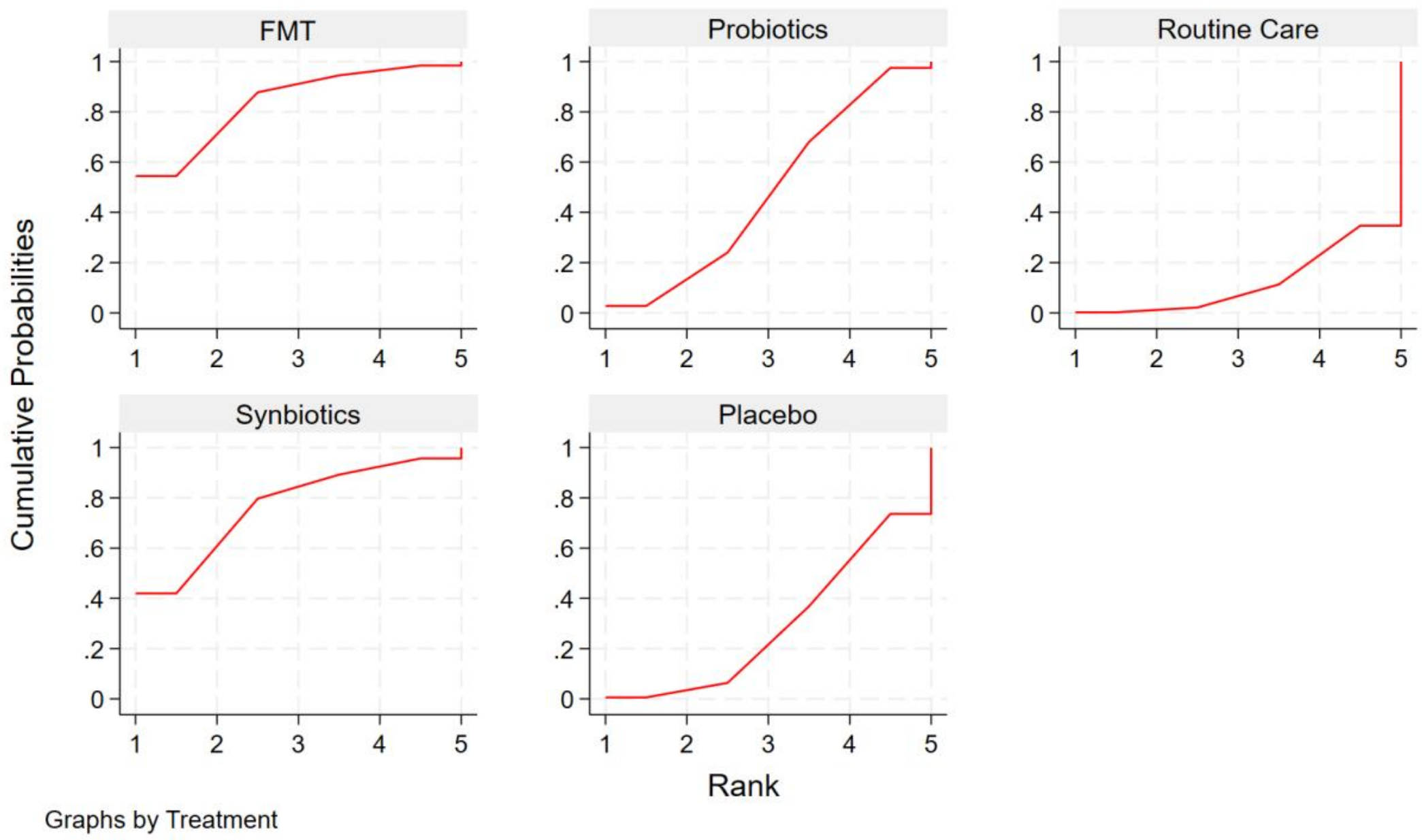

Table S9.1A： SUCRA values and probability rankings of various gut microbiota-targeted therapies for MDS-UPDRS III scores.

Treatment	SUCRA	PrBest	MeanRank
**FMT**	83.8	54.5	1.6
**Probiotics**	48.1	2.8	3.1
**Routine Care**	12.1	0.2	4.5
**Synbiotics**	76.7	42	1.9
**Placebo**	29.4	0.6	3.8

Table S9.1B：Network meta-analysis league table for MDS-UPDRS III scores.

Data are presented as mean differences (MDs) with accompanied 95% confidence intervals (95% CIs). Each cell represents the comparative estimate of the row intervention relative to the column intervention. For negative outcomes such as MDS-UPDRS III scores, an MD < 0 favors the row intervention, whereas an MD > 0 favors the column intervention. For instance, the MD in MDS-UPDRS III scores for FMT compared with Probiotics was -2.72 (95% CI: -7.21 to 1.76). Statistically significant results are highlighted in bold.

FMT				
-2.72 (-7.21,1.76)	Probiotics			
-4.41 (-8.84,0.02)	-1.69 (-3.90,0.52)	Routine Care		
-0.48 (-6.76,5.80)	2.24 (-2.97,7.46)	3.93 (-1.80,9.67)	Synbiotics	
-3.47 (-7.95,1.01)	-0.75 (-3.73,2.24)	0.94 (-2.57,4.46)	-2.99 (-7.31,1.33)	Placebo

Figure S9.2：Cumulative ranking curve plots of gut microbiota-targeted therapies for SBM frequency. A larger surface under the cumulative ranking curve (SUCRA) reflects a higher probability of being positioned as a more effective treatment for increasing SBM frequency.

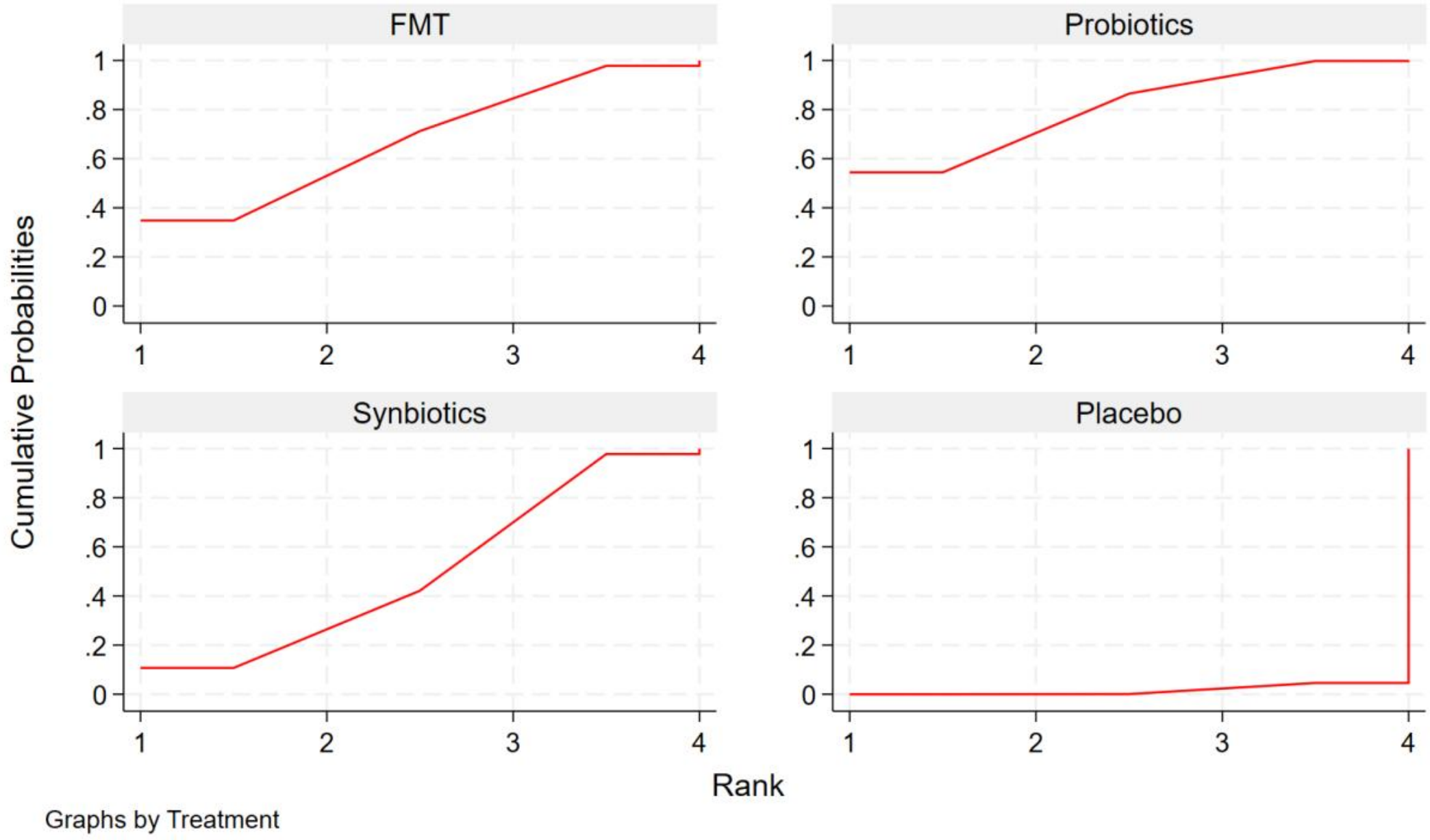

Table S9.2A： SUCRA values and probability rankings of various gut microbiota-targeted therapies for SBM frequency.

Treatment	SUCRA	PrBest	MeanRank
**FMT**	68	34.8	2
**Probiotics**	80.2	54.4	1.6
**Synbiotics**	50.2	10.7	2.5
**Placebo**	1.6	0	4

Table S9.2B：Network meta-analysis league table for SBM frequency.

Data are presented as mean differences (MDs) with accompanied 95% confidence intervals (95% CIs). Each cell represents the comparative estimate of the row intervention relative to the column intervention. For positive clinical outcomes such as SBM frequency, an MD > 0 favors the row intervention, whereas an MD < 0 favors the column intervention. For instance, the MD in SBM frequency for Probiotics compared with Placebo was 2.01 (95% CI: 0.64 to 3.39). Statistically significant results are highlighted in bold.

FMT			
-0.30 (-2.50,1.89)	Probiotics		
0.48 (-1.62,2.58)	0.78 (-1.06,2.62)	Synbiotics	
1.71 (-0.00,3.42)	2.01 (0.64,3.39)	1.23 (0.01,2.45)	Placebo

Figure S9.3：Cumulative ranking curve plots of gut microbiota-targeted therapies for gastrointestinal symptoms. A larger surface under the cumulative ranking curve (SUCRA) reflects a higher probability of being positioned as a more effective treatment for reducing gastrointestinal symptoms.

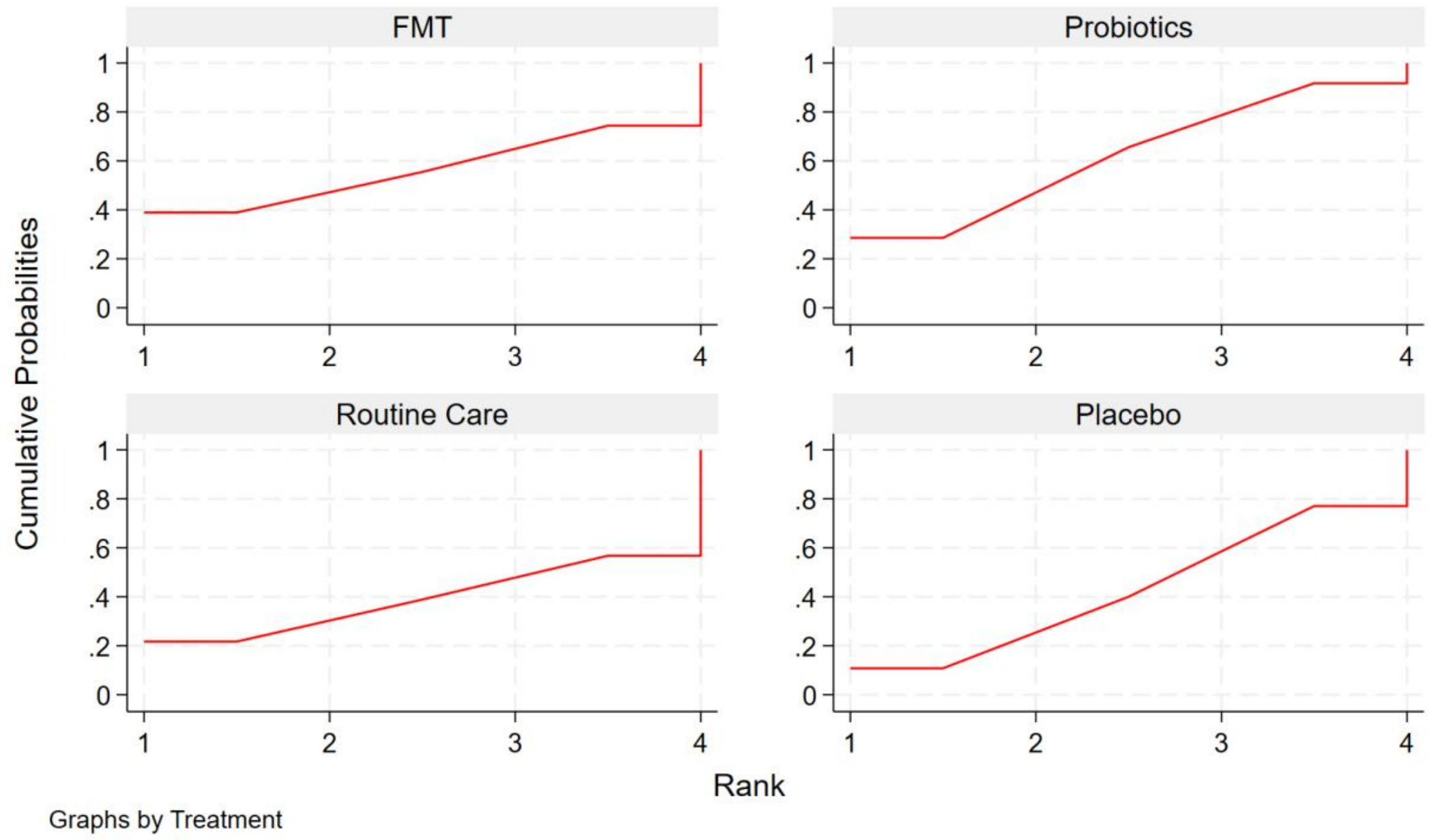

Table S9.3A：SUCRA values and probability rankings of various gut microbiota-targeted therapies for gastrointestinal symptoms.

Treatment	SUCRA	PrBest	MeanRank
**FMT**	56.3	38.9	2.3
**Probiotics**	62	28.6	2.1
**Routine Care**	39.1	21.7	2.8
**Placebo**	42.6	10.8	2.7

Table S9.3B：Network meta-analysis league table for gastrointestinal symptoms. Data are presented as mean differences (MDs) with accompanied 95% confidence intervals (95% CIs). Each cell represents the comparative estimate of the row intervention relative to the column intervention. For negative outcomes such as gastrointestinal symptoms, an MD < 0 favors the row intervention, whereas an MD > 0 favors the column intervention. For instance, the MD in gastrointestinal symptoms for Routine Care compared with Placebo was 1.31 (95% CI: -20.61 to 23.23). Statistically significant results are highlighted in bold.

FMT			
0.29 (-21.41,21.99)	Probiotics		
-3.76 (-31.84,24.32)	-4.05 (-21.98,13.88)	Routine Care	
-2.45 (-20.22,15.33)	-2.74 (-15.40,9.93)	1.31 (-20.61,23.23)	Placebo

Appendix 10：Subgroup analysis based on short-term efficacy

Figure S10.1：Cumulative ranking curve plots of gut microbiota-targeted therapies for MDS-UPDRS III scores. A larger surface under the cumulative ranking curve (SUCRA) reflects a higher probability of being positioned as a more effective treatment for reducing MDS-UPDRS III scores.

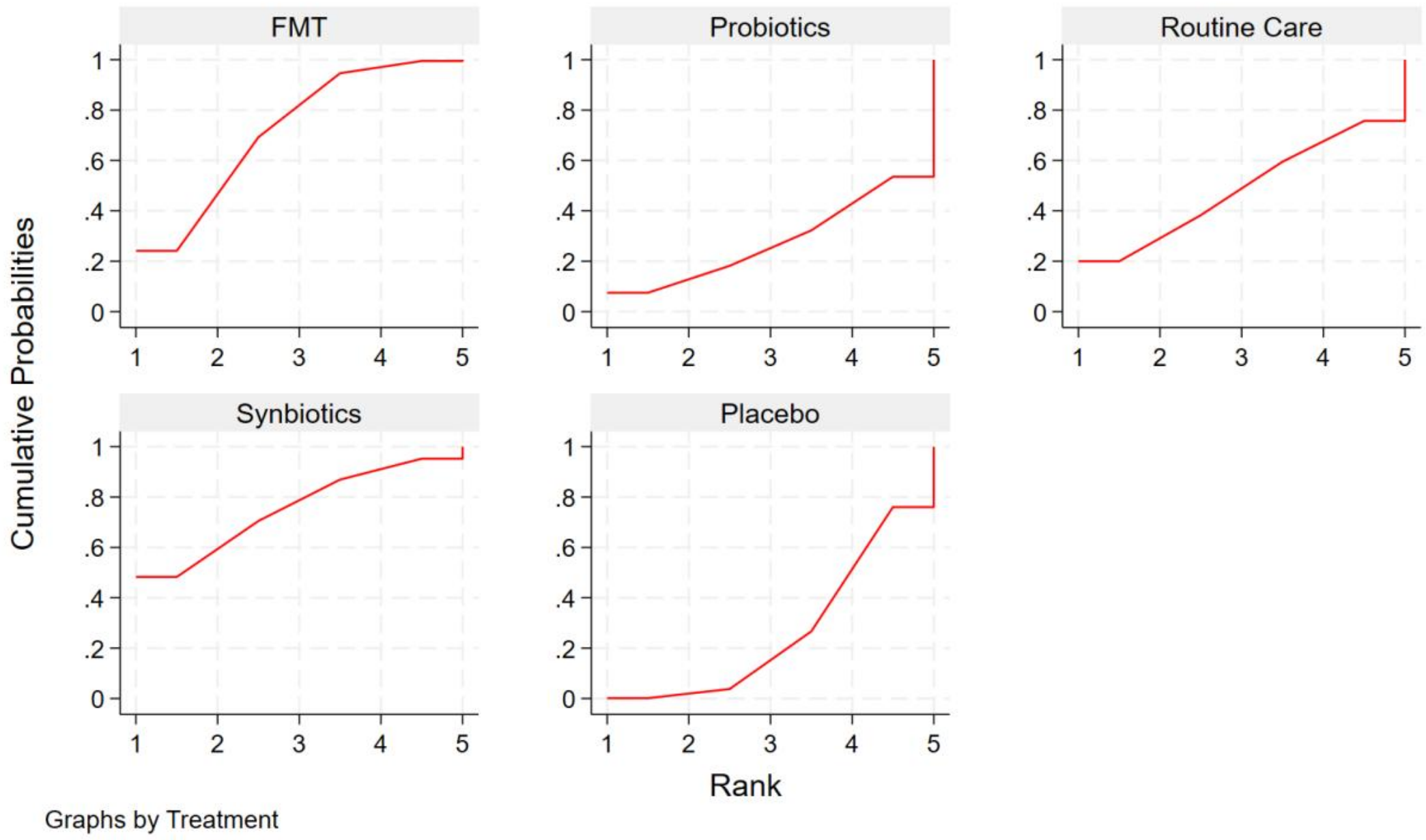

Table S10.1A ： SUCRA values and probability rankings of various gut microbiota-targeted therapies for MDS-UPDRS III scores.

Treatment	SUCRA	PrBest	MeanRank
**FMT**	71.9	24.1	2.1
**Probiotics**	27.9	7.5	3.9
**Routine Care**	48.4	20	3.1
**Synbiotics**	75.2	48.3	2
**Placebo**	26.6	0.1	3.9

Table S10.1B：Network meta-analysis league table for MDS-UPDRS III scores. Data are presented as mean differences (MDs) with accompanied 95% confidence intervals (95% CIs). Each cell represents the comparative estimate of the row intervention relative to the column intervention. For negative outcomes such as MDS-UPDRS III scores, an MD < 0 favors the row intervention, whereas an MD > 0 favors the column intervention. For instance, the MD in MDS-UPDRS III scores for FMT compared with Probiotics was -3.91 (95% CI: -12.07 to 4.25). Statistically significant results are highlighted in bold.

FMT				
-3.91 (-12.07,4.25)	Probiotics			
-1.72 (-9.31,5.87)	2.19 (-8.95,13.33)	Routine Care		
0.90 (-6.43,8.22)	4.81 (-5.13,14.74)	2.62 (-7.93,13.16)	Synbiotics	
-3.26 (-6.74,0.22)	0.65 (-6.72,8.01)	-1.54 (-9.89,6.81)	-4.16 (-10.78,2.47)	Placebo

Figure S10.2：Cumulative ranking curve plots of gut microbiota-targeted therapies for SBM frequency. A larger surface under the cumulative ranking curve (SUCRA) reflects a higher probability of being positioned as a more effective treatment for increasing SBM frequency.

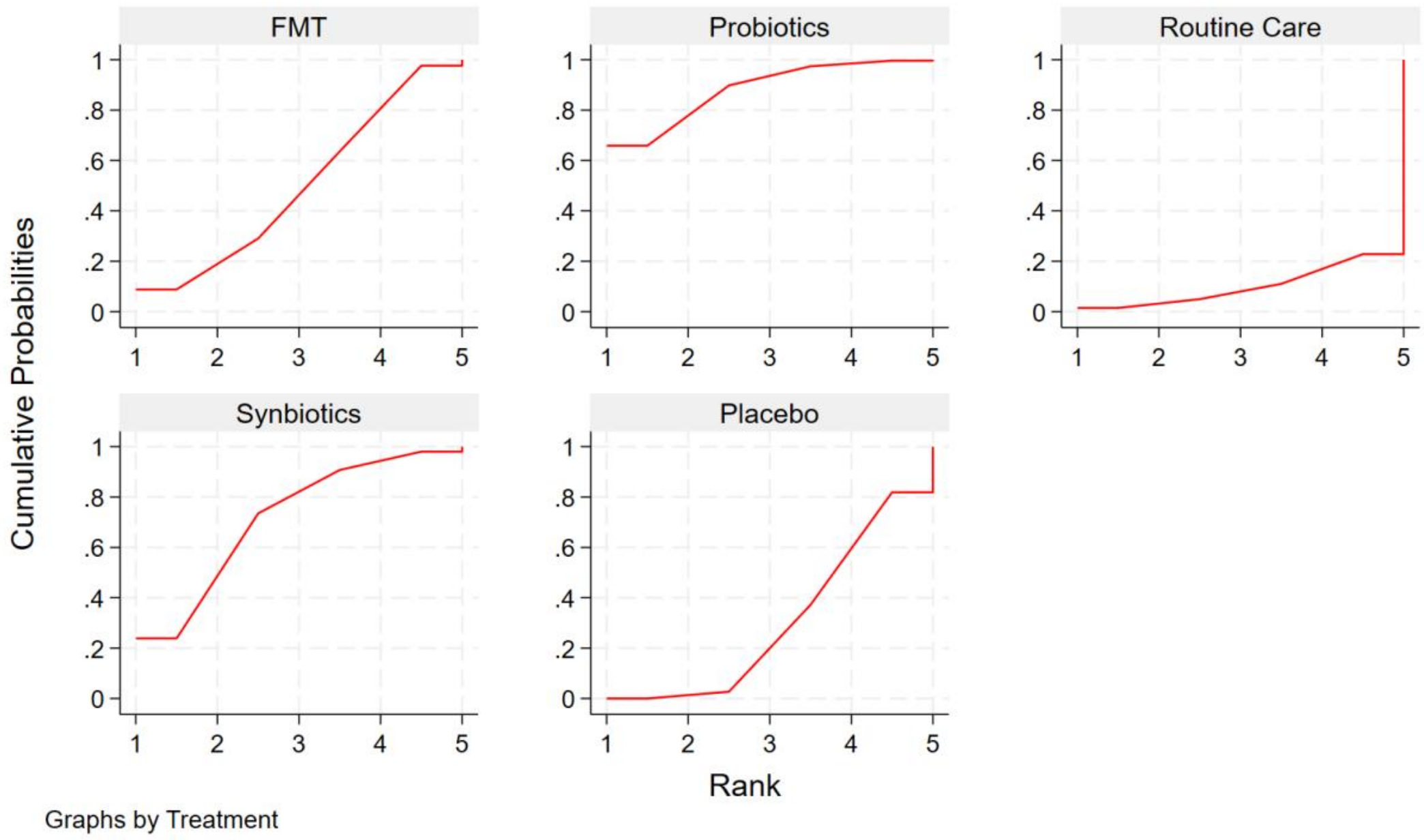

Table S10.2A： SUCRA values and probability rankings of various gut microbiota-targeted therapies for SBM frequency.

Treatment	SUCRA	PrBest	MeanRank
**FMT**	49.8	8.8	3
**Probiotics**	88.2	65.9	1.5
**Routine Care**	10.1	1.4	4.6
**Synbiotics**	71.5	23.9	2.1
**Placebo**	30.5	0	3.8

Table S10.2B：Network meta-analysis league table for SBM frequency.

Data are presented as mean differences (MDs) with accompanied 95% confidence intervals (95% CIs). Each cell represents the comparative estimate of the row intervention relative to the column intervention. For positive clinical outcomes such as SBM frequency, an MD > 0 favors the row intervention, whereas an MD < 0 favors the column intervention. For instance, the MD in SBM frequency for Probiotics compared with Placebo was 1.93 (95% CI: 0.23 to 3.64). Statistically significant results are highlighted in bold.

FMT				
-1.51 (-4.20,1.17)	Probiotics			
1.71 (-0.55,3.97)	3.22 (-0.28,6.73)	Routine Care		
-0.81 (-3.43,1.80)	0.70 (-1.64,3.04)	-2.52 (-5.98,0.93)	Synbiotics	
0.42 (-1.65,2.49)	1.93 (0.23,3.64)	-1.29 (-4.35,1.77)	1.23 (-0.37,2.84)	Placebo

Appendix 11：Subgroup analysis of medium-to-long-term efficacy

Figure S11.1：Cumulative ranking curve plots of gut microbiota-targeted therapies for MDS-UPDRS III scores. A larger surface under the cumulative ranking curve (SUCRA) reflects a higher probability of being positioned as a more effective treatment for reducing MDS-UPDRS III scores.

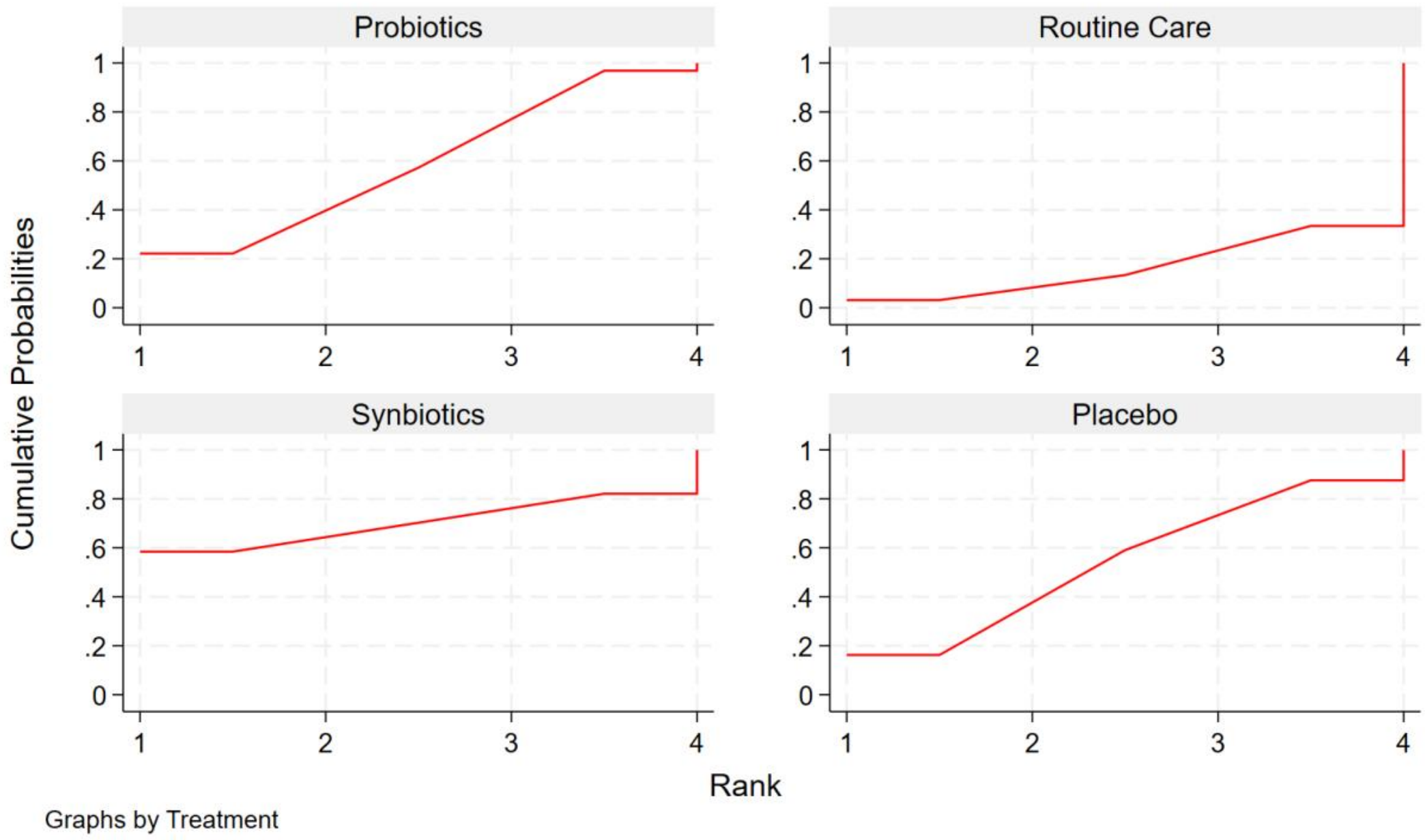

Table S11.1A： SUCRA values and probability rankings of various gut microbiota-targeted therapies for MDS-UPDRS III scores.

Treatment	SUCRA	PrBest	MeanRank
**Probiotics**	58.8	22.2	2.2
**Routine Care**	16.6	3.1	3.5
**Synbiotics**	70.3	58.5	1.9
**Placebo**	54.3	16.2	2.4

Table S11.1B：Network meta-analysis league table for MDS-UPDRS III scores.

Data are presented as mean differences (MDs) with accompanied 95% confidence intervals (95% CIs). Each cell represents the comparative estimate of the row intervention relative to the column intervention. For negative outcomes such as MDS-UPDRS III scores, an MD < 0 favors the row intervention, whereas an MD > 0 favors the column intervention. For instance, the MD in MDS-UPDRS III scores for Probiotics compared with Routine Care was -2.35 (95% CI: -5.65 to 0.95). Statistically significant results are highlighted in bold.

Probiotics			
-2.35 (-5.65,0.95)	Routine Care		
1.92 (-7.40,11.24)	4.27 (-5.64,14.17)	Synbiotics	
-0.05 (-4.26,4.17)	2.30 (-3.09,7.69)	-1.97 (-10.29,6.35)	Placebo

Appendix 12：Sensitivity analysis excluding uncertain studies

Figure S12.1：UPDRS III

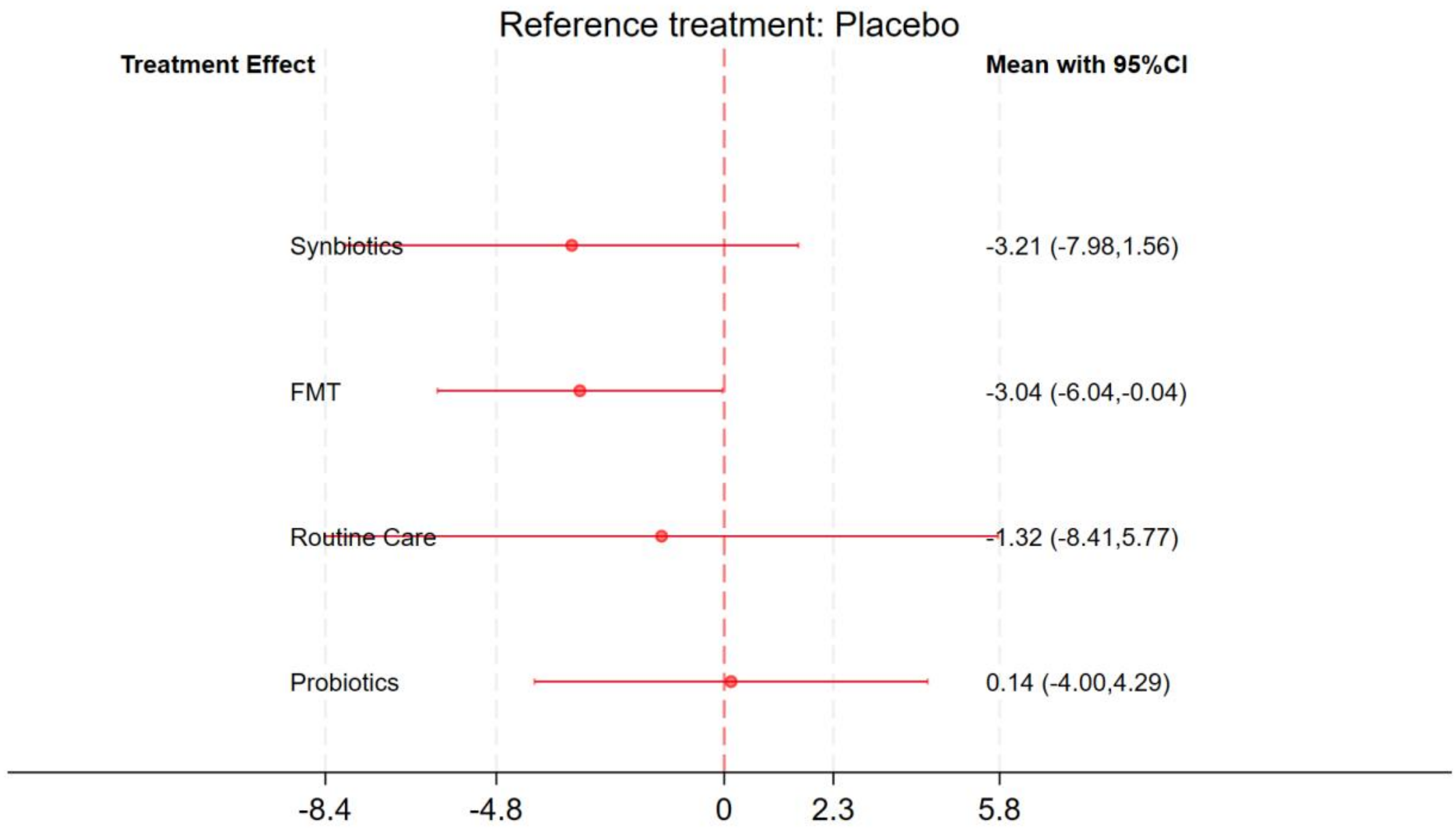

Figure S12.2：SBM

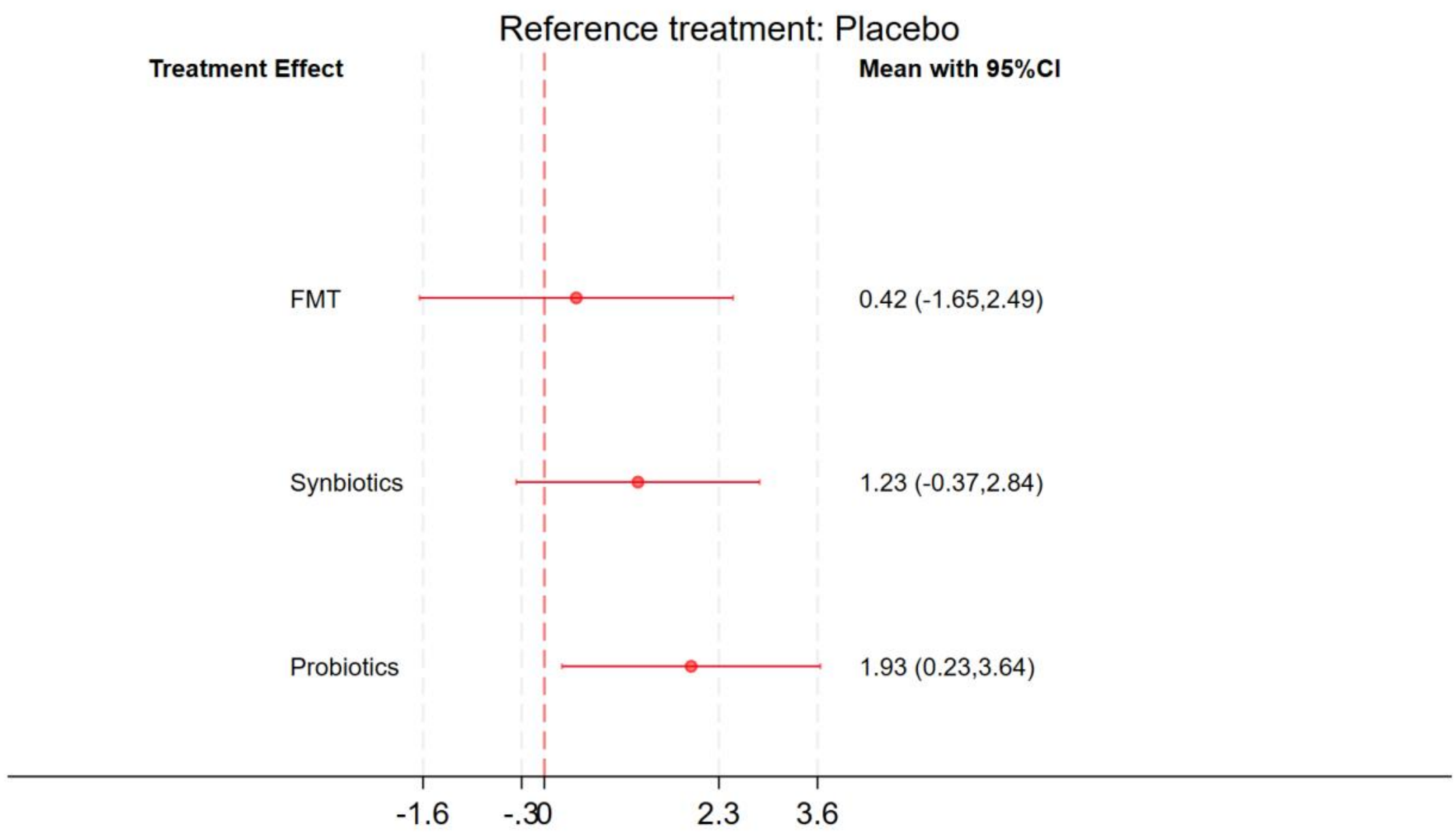

Figure S12.3：Gastrointestinal Symptom

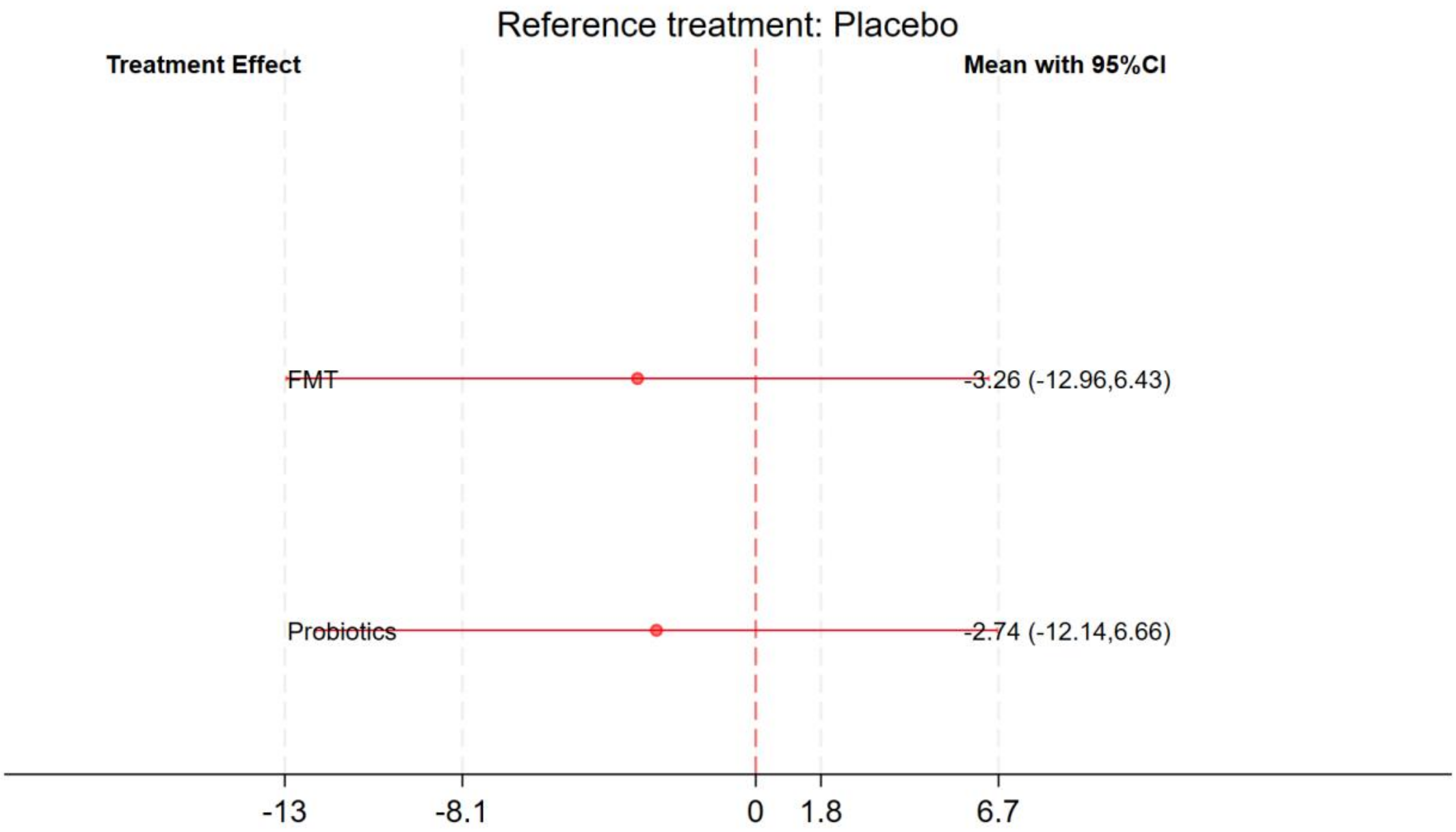

Appendix 13：Sensitivity analysis after excluding small-sample studies

Figure S13.1：UPDRS III

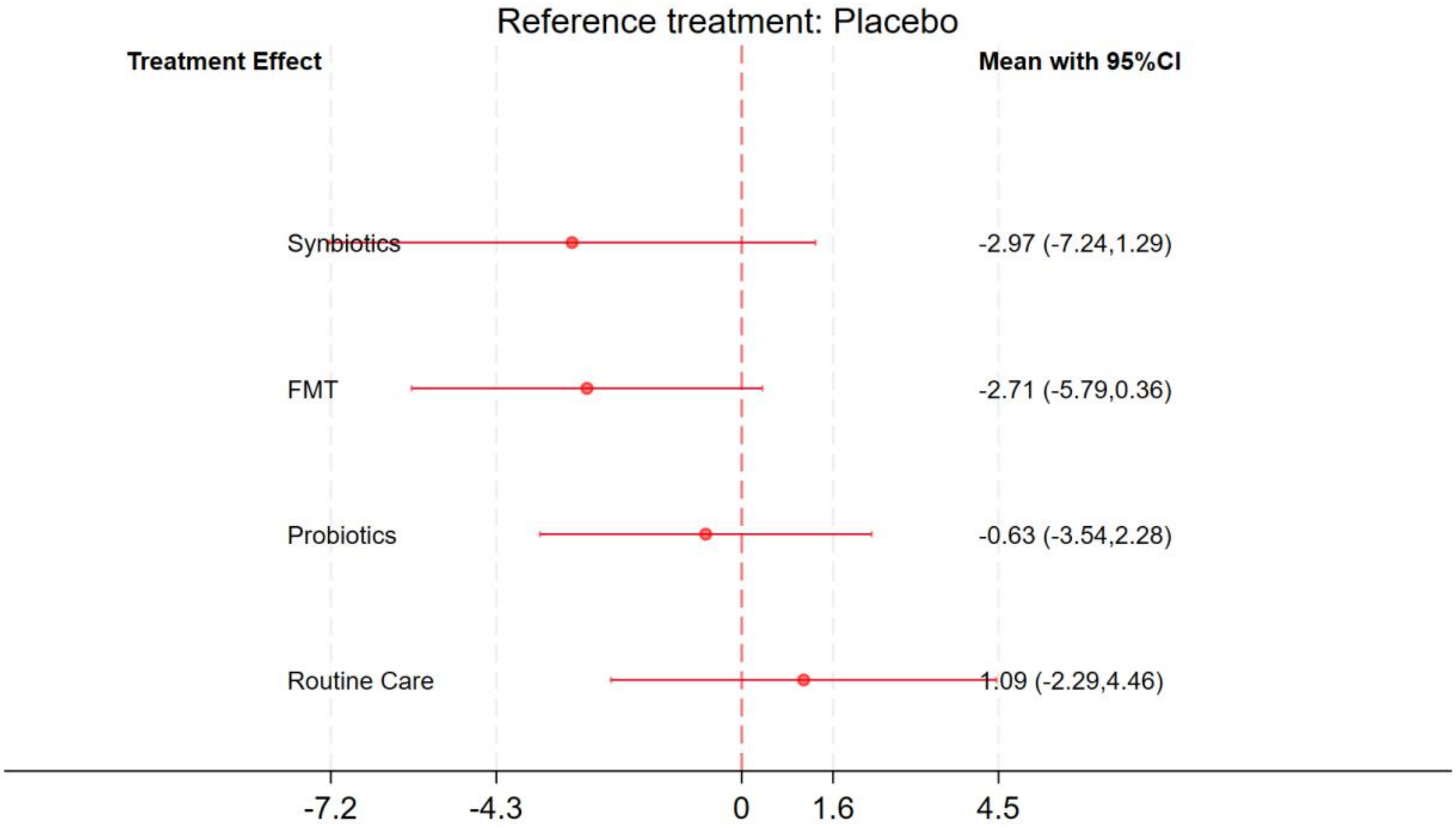

Figure S13.2：SBM

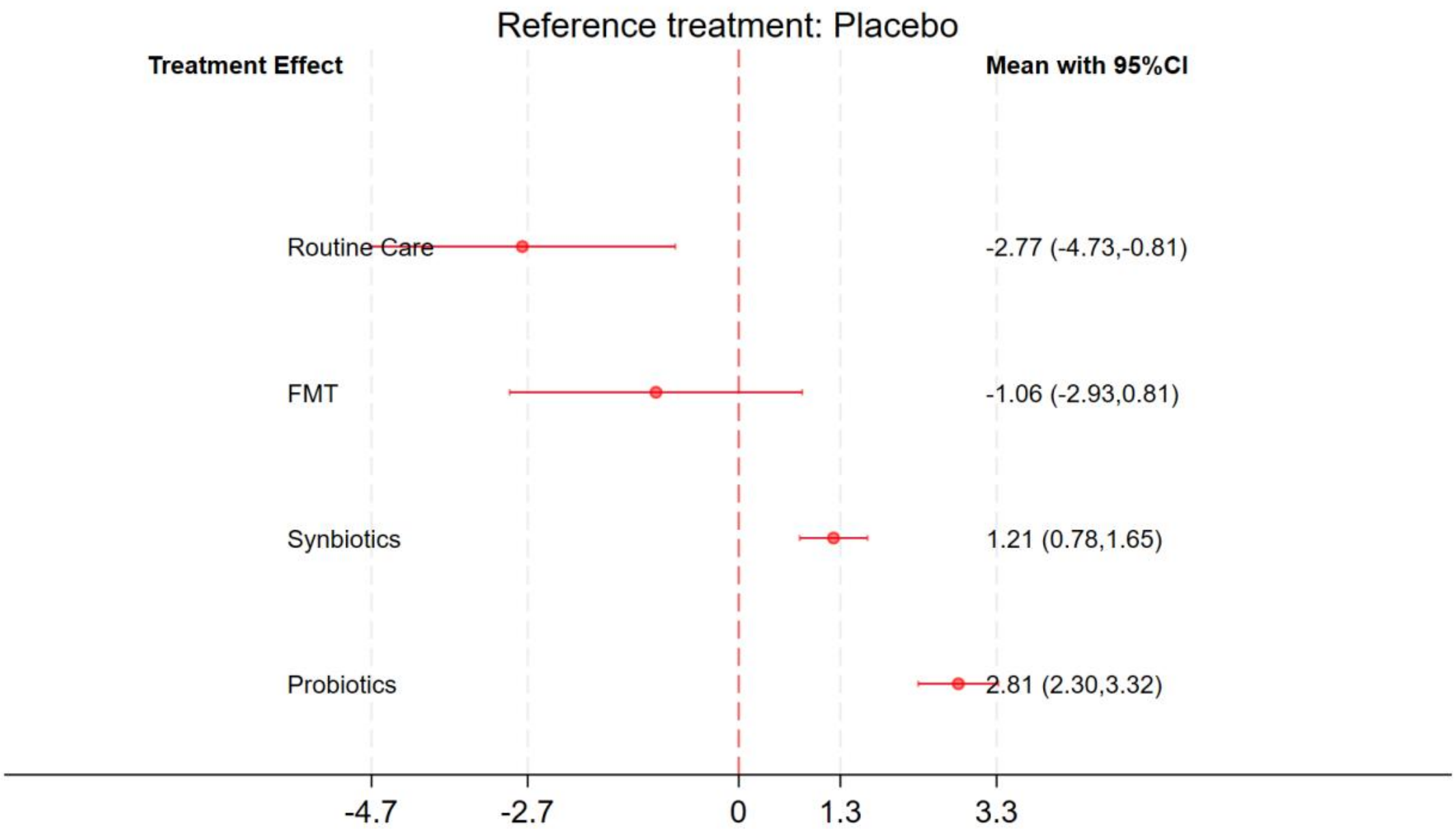

Figure S13.3：Gastrointestinal Symptom

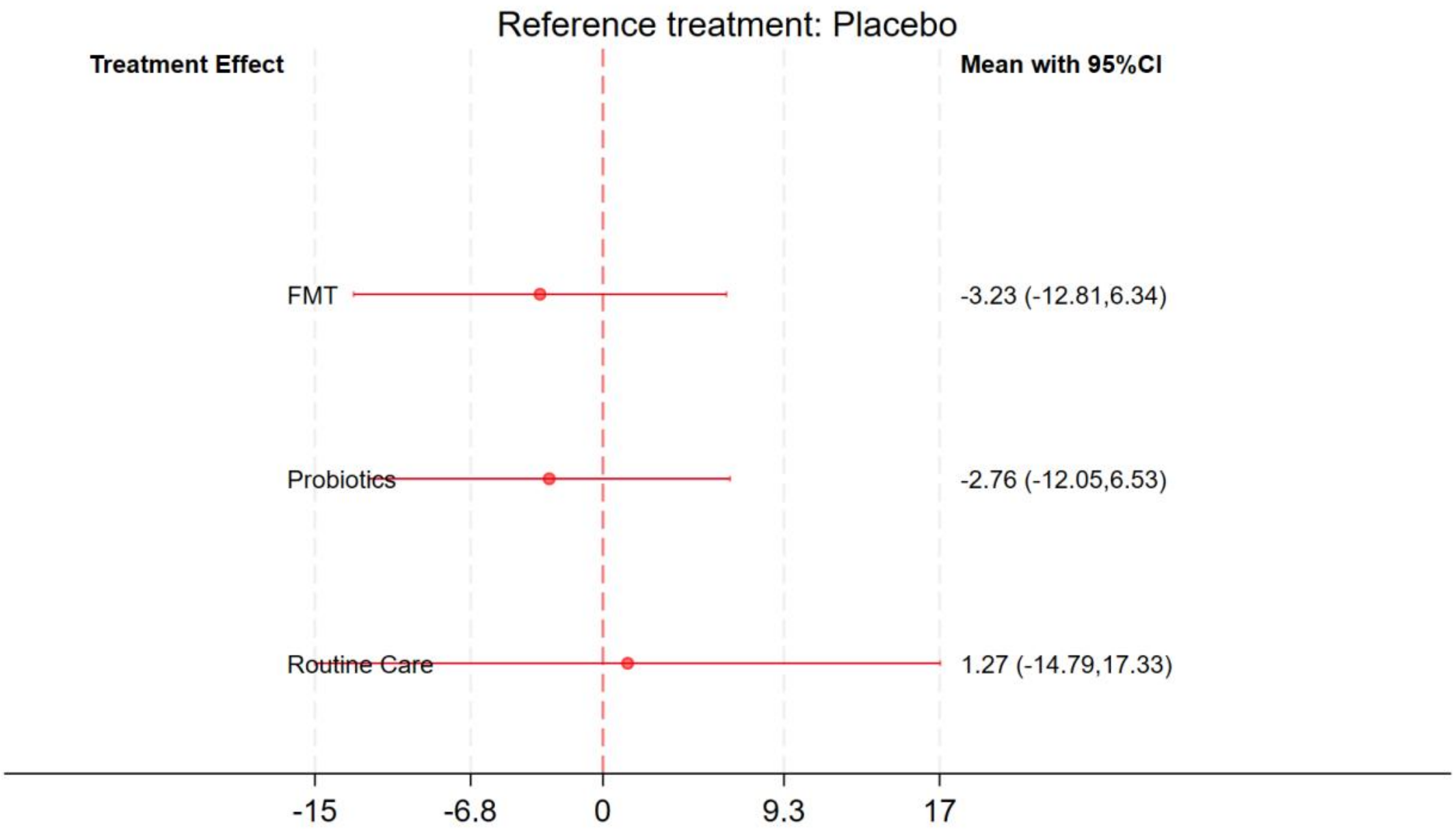

Appendix 14: CINeMA Assessment

Figure S14.1: Risk of bias contribution in UPDRS III

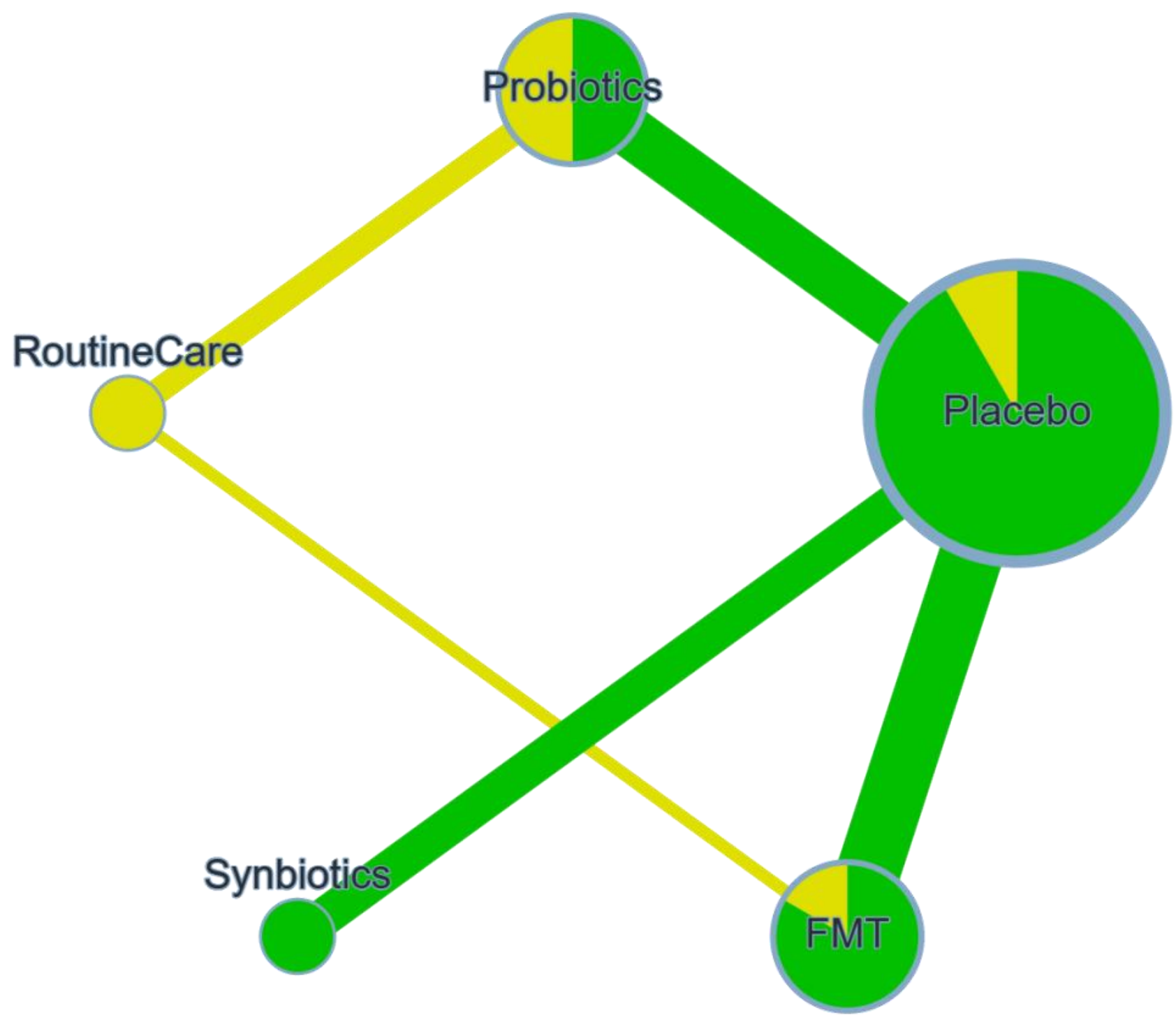

Figure S14.2: Overall risk of bias by treatment comparison in UPDRS III

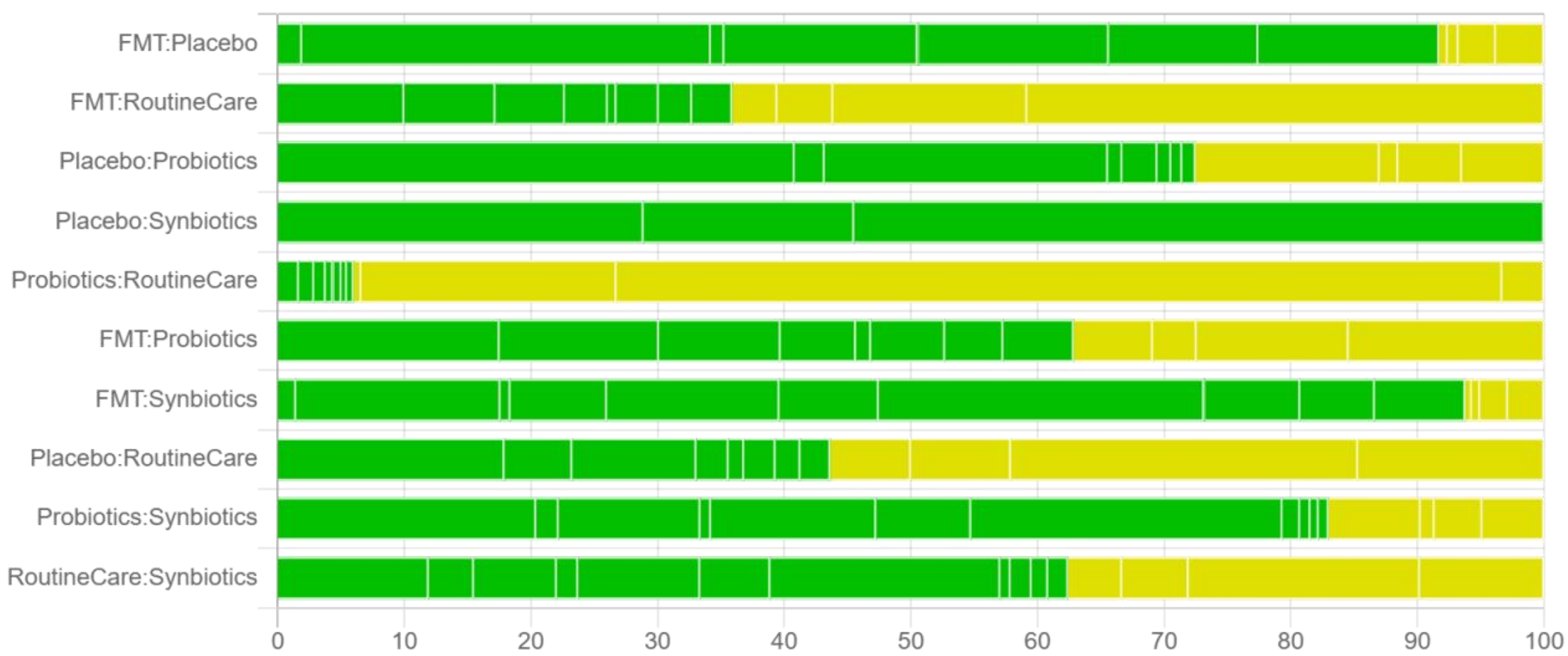

Figure S14.3: Risk of bias contribution in SBM

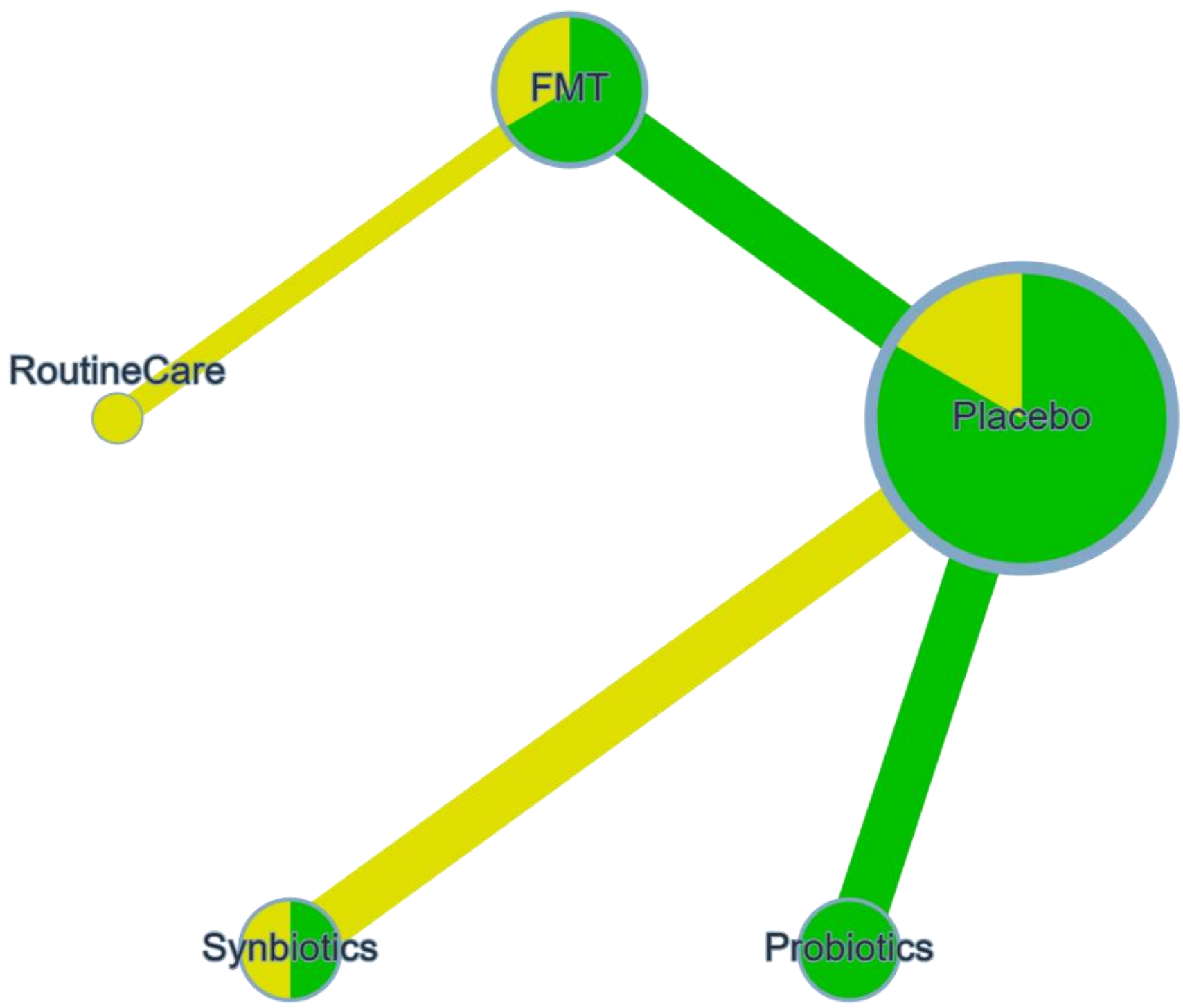

Figure S14.4: Overall risk of bias in SBM

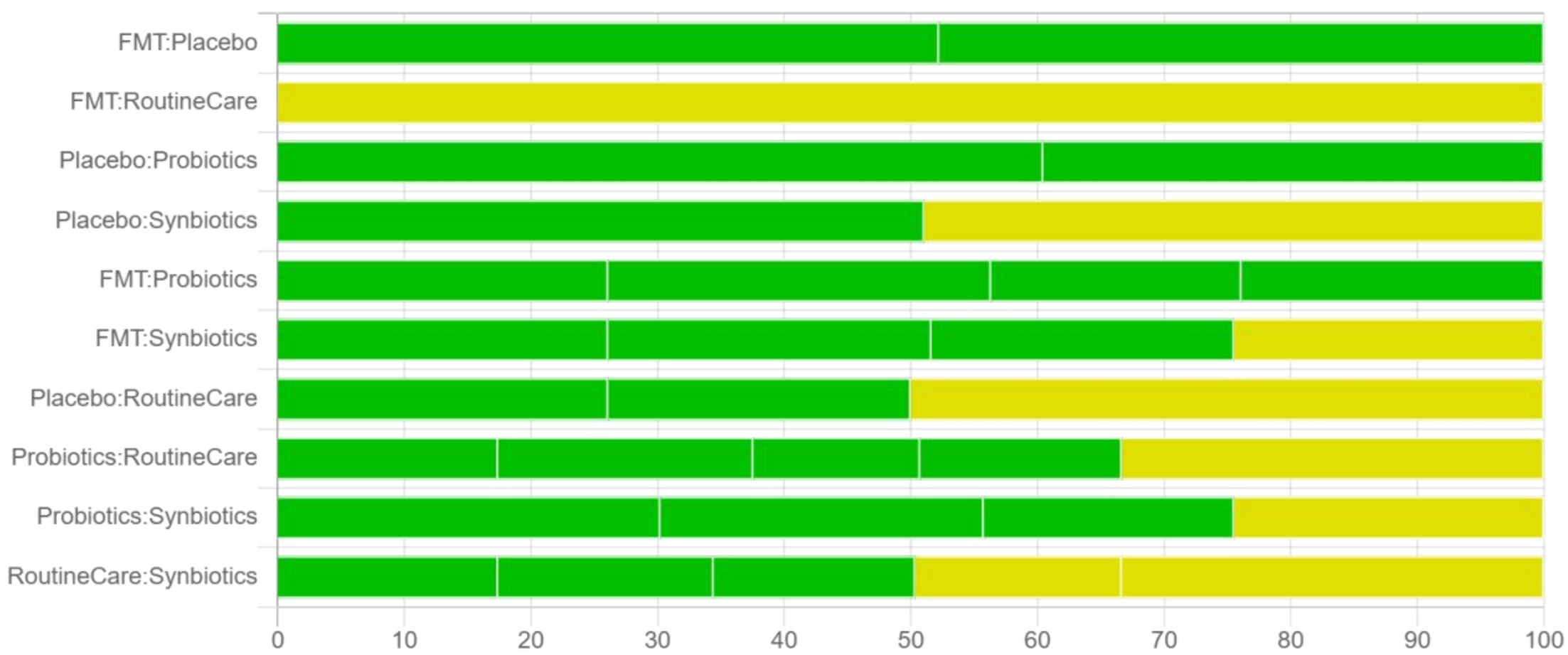

Figure S14.5: Risk of bias contribution in Gastrointestinal Symptom

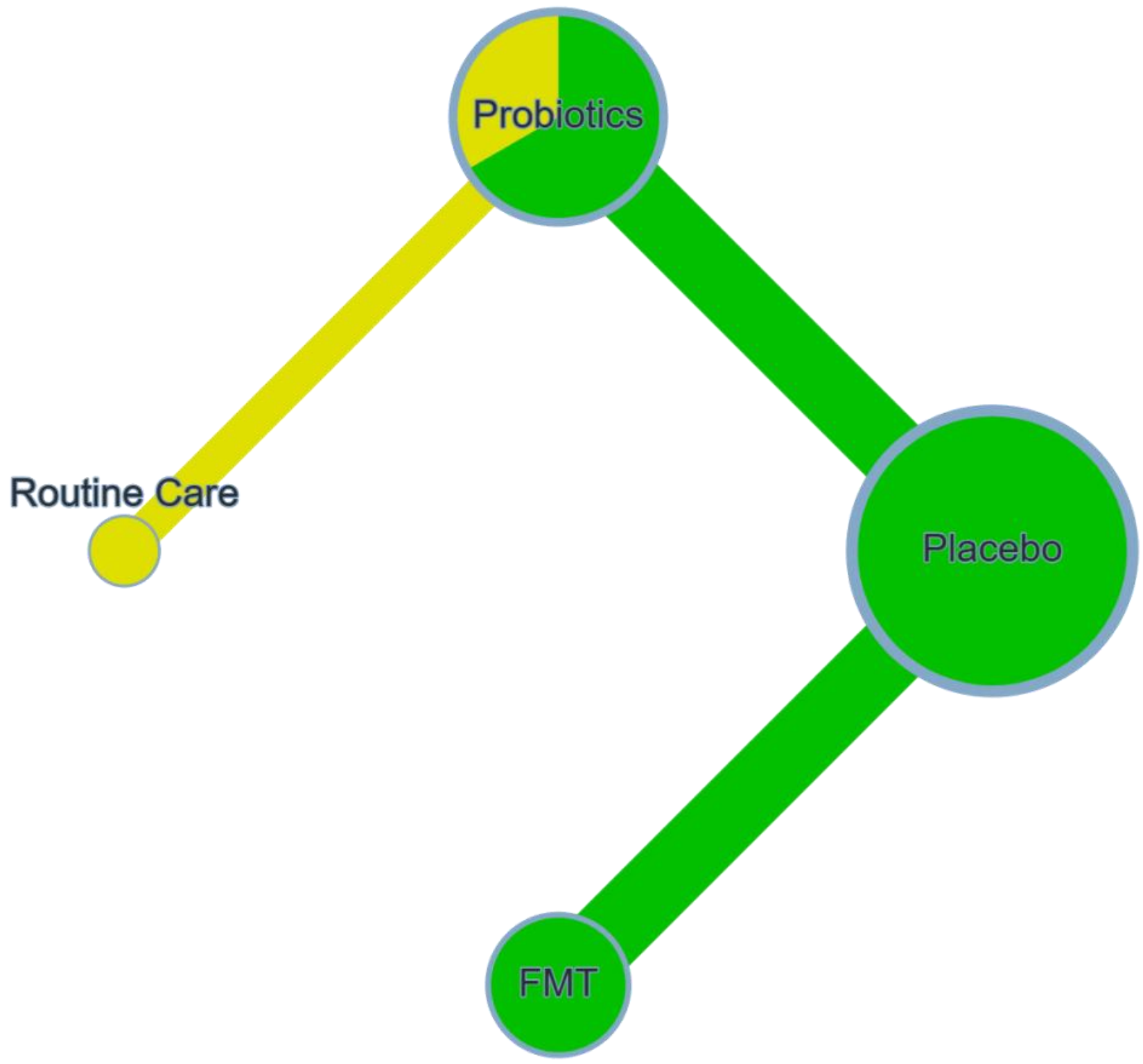

Figure S14.6: Overall risk of bias in Gastrointestinal Symptom

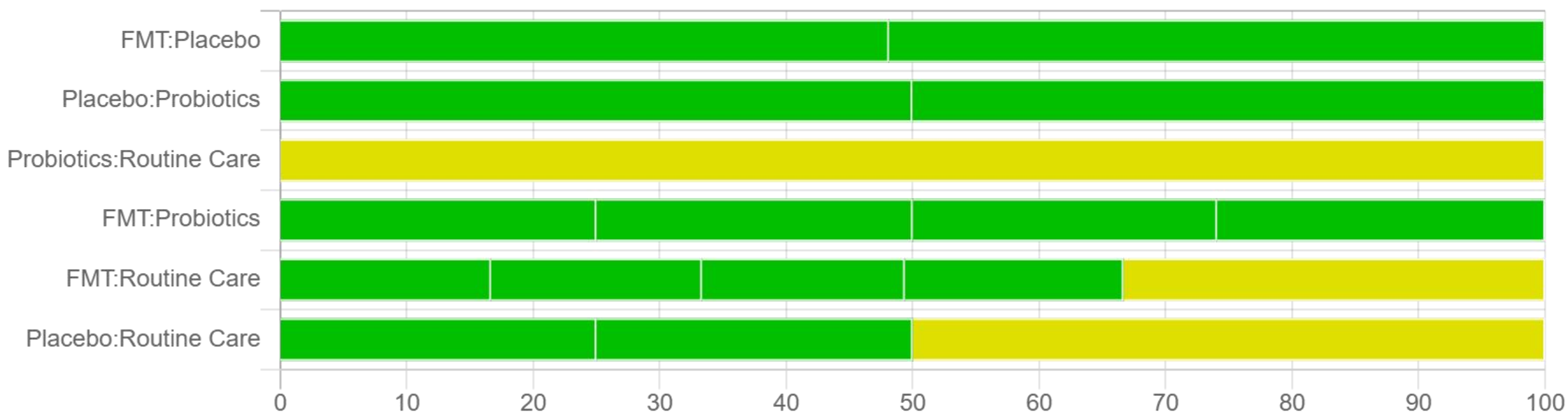

Table S14.1: CINeMA Results of UPDRS III

Comparison	Within-study bias	Reporting bias	Indirectness	Imprecision	Heterogeneity	Incoherence	Confidence rating
FMT:Placebo	No concerns	Low risk	No concerns	No concerns	Major concerns	No concerns	Very low
FMT:Routine Care	Some concerns	Low risk	No concerns	No concerns	Major concerns	No concerns	Very low
Placebo:Probiotics	No concerns	Low risk	No concerns	Major concerns	No concerns	No concerns	Low
Placebo:Synbiotics	No concerns	Low risk	No concerns	Major concerns	No concerns	No concerns	Low
Probiotics:Routine Care	Some concerns	Low risk	No concerns	Major concerns	No concerns	No concerns	Very low
FMT:Probiotics	No concerns	Low risk	No concerns	Major concerns	No concerns	No concerns	Low
FMT:Synbiotics	No concerns	Low risk	No concerns	Major concerns	No concerns	No concerns	Low
Placebo:Routine Care	Some concerns	Low risk	No concerns	Major concerns	No concerns	No concerns	Very low
Probiotics:Synbiotics	No concerns	Low risk	No concerns	Major concerns	No concerns	No concerns	Low
Routine Care:Synbiotics	No concerns	Low risk	No concerns	Major concerns	No concerns	No concerns	Low

Table S14.2: CINeMA Results of SBM

Comparison	Within-study bias	Reporting bias	Indirectness	Imprecision	Heterogeneity	Incoherence	Confidence rating
FMT:Placebo	No concerns	Low risk	No concerns	Major concerns	No concerns	No concerns	Very low
FMT:Routine Care	Some concerns	Low risk	No concerns	No concerns	Major concerns	No concerns	Very low
Placebo:Probiotics	No concerns	Low risk	No concerns	No concerns	Major concerns	No concerns	Moderate
Placebo:Synbiotics	No concerns	Low risk	No concerns	No concerns	Major concerns	No concerns	Low
FMT:Probiotics	No concerns	Low risk	No concerns	Major concerns	No concerns	No concerns	Very low
FMT:Synbiotics	No concerns	Low risk	No concerns	Major concerns	No concerns	No concerns	Very low
Placebo:Routine Care	No concerns	Low risk	No concerns	Major concerns	No concerns	No concerns	Very low
Probiotics:Routine Care	No concerns	Low risk	No concerns	No concerns	Major concerns	No concerns	Very low
Probiotics:Synbiotics	No concerns	Low risk	No concerns	Major concerns	No concerns	No concerns	Very low
Routine Care:Synbiotics	No concerns	Low risk	No concerns	Major concerns	No concerns	No concerns	Very low
FMT:Placebo	No concerns	Low risk	No concerns	Major concerns	No concerns	No concerns	Very low

Table S14.3: CINeMA Results of Gastrointestinal Symptom

Comparison	Within-study bias	Reporting bias	Indirectness	Imprecision	Heterogeneity	Incoherence	Confidence rating
FMT:Placebo	No concerns	Low risk	No concerns	Major concerns	No concerns	No concerns	Very low
Placebo:Probiotics	No concerns	Low risk	No concerns	Major concerns	No concerns	No concerns	Low
Probiotics:Routine Care	Some concerns	Low risk	No concerns	Major concerns	No concerns	No concerns	Very low
FMT:Probiotics	No concerns	Low risk	No concerns	Major concerns	No concerns	No concerns	Very low
FMT:Routine Care	No concerns	Low risk	No concerns	Major concerns	No concerns	No concerns	Very low
Placebo:Routine Care	No concerns	Low risk	No concerns	Major concerns	No concerns	No concerns	Very low

Appendix 15: PRISMA Checklist and Graphical Asset

Section and Topic	Item #	Checklist item	Location where item is reported
**TITLE**			
Title	1	Identify the report as a systematic review.	Title (Page 1)
**ABSTRACT**			
Abstract	2	See the PRISMA 2020 for Abstracts checklist.	Abstract (Page1): Structured under Background, Methods, Results, and Conclusion
**INTRODUCTION**			
Rationale	3	Describe the rationale for the review in the context of existing knowledge.	Introduction (Pages2-3): Outlined across paragraphs 1-4 (Pathophysiological translation, therapeutic potential, and current clinical gaps in microecological configurations).
Objectives	4	Provide an explicit statement of the objective(s) or question(s) the review addresses.	Introduction (Page 2, Last Paragraph): Explicitly stated: "...this study aims to evaluate and rank the efficacy of various gut microbiota-targeted therapies on both motor and non-motor symptoms in patients with PD..."
**METHODS**			
Eligibility criteria	5	Specify the inclusion and exclusion criteria for the review and how studies were grouped for the syntheses.	Methods - Eligibility & Exclusion Criteria (Pages 3-4): Structured using the PICOS framework. Merging rules into core foundational nodes (FMT, Probiotics, Synbiotics) are detailed on Page 4.
Information sources	6	Specify all databases, registers, websites, organisations, reference lists and other sources searched or consulted to identify studies. Specify the date when each source was last searched or consulted.	Methods - Search Strategy (Page 6): Primary databases (PubMed, Embase, Web of Science, Cochrane CENTRAL, CNKI, Wanfang, VIP,

			CBM), registries (ClinicalTrials.gov, ICTRP, ChiCTR), and grey literature searched up to April 1, 2026.
Search strategy	7	Present the full search strategies for all databases, registers and websites, including any filters and limits used.	Methods - Search Strategy (Page 6): Noted in text; full string breakdowns for each electronic source are provided in Appendix 1 (Tables S1–S5).
Selection process	8	Specify the methods used to decide whether a study met the inclusion criteria of the review, including how many reviewers screened each record and each report retrieved, whether they worked independently, and if applicable, details of automation tools used in the process.	Methods - Data Extraction (Page 5): Two reviewers independently executed title/abstract screening and subsequent full-text validation; conflicts resolved by a third reviewer.
Data collection process	9	Specify the methods used to collect data from reports, including how many reviewers collected data from each report, whether they worked independently, any processes for obtaining or confirming data from study investigators, and if applicable, details of automation tools used in the process.	Methods - Data Extraction (Page 5): Standardized extraction framework executed independently by two reviewers; discrepancies resolved via a third reviewer.
Data items	10a	List and define all outcomes for which data were sought. Specify whether all results that were compatible with each outcome domain in each study were sought (e.g. for all measures, time points, analyses), and if not, the methods used to decide which results to collect.	Methods - Outcome Measures (Page 5): Defined 2 primary endpoints (MDS-UPDRS III and SBM frequency) and secondary metrics (total UPDRS, gastrointestinal symptoms, anxiety, depression, sleep, cognitive scores, PDQ-39, TAC, and MDA).
	10b	List and define all other variables for which data were sought (e.g. participant and intervention characteristics, funding sources). Describe any assumptions made about any missing or unclear information.	Methods - Data Extraction & Statistical Analysis (Pages 4-7): Extracted baseline modifiers (age, sex, disease duration, BMI, geographic distribution). Missing $SD_{\triangle}$ handled via Cochrane formula (R=0.5), medians handled via Wan/Luo transformations.
Study risk of bias assessment	11	Specify the methods used to assess risk of bias in the included studies, including details of the tool(s) used, how many reviewers assessed each study and whether they worked independently, and if applicable, details of automation tools used in the process.	Methods - Quality Assessment of Evidence (Page 5-6): Cochrane Risk of Bias 2.0 tool applied

			independently by two reviewers.
Effect measures	12	Specify for each outcome the effect measure(s) (e.g. risk ratio, mean difference) used in the synthesis or presentation of results.	Methods - Statistical Analysis (Page 6): Expressed as mean differences (MD) or standardized mean differences (SMD) alongside 95% confidence intervals (CIs).
Synthesis methods	13a	Describe the processes used to decide which studies were eligible for each synthesis (e.g. tabulating the study intervention characteristics and comparing against the planned groups for each synthesis (item #5)).	Methods - Principles for Merging Intervention Nodes (Page 7): Described specific biological and clinical homogeneity criteria utilized for compiling cohorts into foundational network nodes.
	13b	Describe any methods required to prepare the data for presentation or synthesis, such as handling of missing summary statistics, or data conversions.	Methods - Statistical Analysis (Pages 6): Variance baseline transformations from medians/IQRs described based on Wan et al. (2014) and Luo et al. (2018) formulas.
	13c	Describe any methods used to tabulate or visually display results of individual studies and syntheses.	Methods - Principles for Merging Intervention Nodes (Page 7): Indicated the execution of network geometry maps, forest trajectories, and SUCRA ranking curve calculations.
	13d	Describe any methods used to synthesize results and provide a rationale for the choice(s). If meta-analysis was performed, describe the model(s), method(s) to identify the presence and extent of statistical heterogeneity, and software package(s) used.	Methods - Statistical Analysis (Page 6): Frequentist random-effects network meta-analysis conducted via Stata 17.0 (mvmeta command); cross-arm correlation within multi-arm trials modeled via multivariate variance-covariance matrix structure; common τ^2 network heterogeneity assumed.
	13e	Describe any methods used to explore possible causes of heterogeneity among study results (e.g. subgroup analysis, meta-regression).	Methods - Principles for Merging Intervention Nodes (Page 7): Outlined hierarchical subgroup analyses pre-stratified by treatment

			tracking windows and route of microecological delivery.
	13f	Describe any sensitivity analyses conducted to assess robustness of the synthesized results.	Methods - Principles for Merging Intervention Nodes (Page 7): Indicated sensitivity test protocols by restricting parameters to low risk-of-bias trials and large-sample cohorts.
Reporting bias assessment	14	Describe any methods used to assess risk of bias due to missing results in a synthesis (arising from reporting biases).	Methods - Risk of bias, certainty of evidence, and consistency (Page 9): Exploratory funnel plots used for visual symmetry mapping; statistical underpower acknowledged for sparse sub-networks.
Certainty assessment	15	Describe any methods used to assess certainty (or confidence) in the body of evidence for an outcome.	Methods - Quality Assessment of Evidence (Page 9): Graded systematically via the Confidence in Network Meta-Analysis (CINeMA) framework.
**RESULTS**			
Study selection	16a	Describe the results of the search and selection process, from the number of records identified in the search to the number of studies included in the review, ideally using a flow diagram.	Results - Literature Selection (Page 8): Flow documented step-by-step from 1,405 initial inputs to 19 selected trials; mapped natively via the Figure 1 PRISMA Flow Diagram.
	16b	Cite studies that might appear to meet the inclusion criteria, but which were excluded, and explain why they were excluded.	Results - Literature Selection (Page 8) & Figure 1: Categorized reasons for full-text exclusion across 208 articles.
Study	17	Cite each included study and present its characteristics.	Results - Table 1 & Table 2 (Pages

characteristics			11-12): Tabulated comprehensive features for all 19 included RCTs. Table 3 (Page 3): Displays pooled effect modifiers.
Risk of bias in studies	18	Present assessments of risk of bias for each included study.	Results - Risk of bias... (Page 10-14) & Figure 2: Summarized Cochrane tool outputs. Trial-level domains detailed in Appendix 3 (Table S3 & Figure S3).
Results of individual studies	19	For all outcomes, present, for each study: (a) summary statistics for each group (where appropriate) and (b) an effect estimate and its precision (e.g. confidence/credible interval), ideally using structured tables or plots.	Results - Appendix 7 (Tables S7.1–S7.11): NMA relative comparison matrix tables provide structured estimates (MD/SMD) and 95% CIs across all outcomes.
Results of syntheses	20a	For each synthesis, briefly summarise the characteristics and risk of bias among contributing studies.	Results - Primary Network Analysis (Pages 14-19): Provided summary statistics and network structures (star-like topology) for each active outcome network. Risk bias contribution outlined via Appendix 14 (Figures S14.1–S14.6).
	20b	Present results of all statistical syntheses conducted. If meta-analysis was done, present for each the summary estimate and its precision (e.g. confidence/credible interval) and measures of statistical heterogeneity. If comparing groups, describe the direction of the effect.	Results - Primary Network Analysis & Figures 3–6 (Pages 14-19): Reports all network comparisons, SUCRA probabilities, and the common heterogeneity variance (τ^2

			= 0.08) across endpoints. League tables detailed in Appendix 7.
	20c	Present results of all investigations of possible causes of heterogeneity among study results.	Results - Subgroup Analysis (Page 21-22) & Figure 7: Documents data mapping for oral delivery vs. endoscopic routes. Data tracking detailed in Appendix 9, 10, & 11.
	20d	Present results of all sensitivity analyses conducted to assess the robustness of the synthesized results.	Results - Sensitivity analyses (Page 20): Detailed shifts in the motor outcome (FMT vs. Placebo: MD = -3.04, 95% CI: -6.04 to -0.04) and non-motor stability. Trajectories provided in Appendix 12 & 13.
Reporting biases	21	Present assessments of risk of bias due to missing results (arising from reporting biases) for each synthesis assessed.	Results - Risk of bias, certainty of evidence, and consistency (Page 9-10) & Table 4: CINeMA assessment results outline reporting bias ratings across key contrasts. Mapped visually via Appendix 8 Funnel Plots.
Certainty of evidence	22	Present assessments of certainty (or confidence) in the body of evidence for each outcome assessed.	Results - Table 4 (Page 13): Complete matrix of CINeMA evaluations. Breakdown tables provided in Appendix 14 (Tables S14.1–S14.3).
**DISCUSSION**			

Discussion	23a	Provide a general interpretation of the results in the context of other evidence.	Discussion - Comparison with Previous Research... & Pathophysiological Translation (Page 23): Discussed findings alongside preclinical rodent systematic reviews and MCID criteria.
	23b	Discuss any limitations of the evidence included in the review.	Discussion - Strengths and Limitations (Page 25-26): Critiqued small-study effects, clinical heterogeneity, and lack of objective metabolic or sequencing biomarkers.
	23c	Discuss any limitations of the review processes used.	Discussion - Strengths and Limitations (Page 25): Acknowledged limitations regarding sparse network geometry, node pooling, and the dominance of indirect comparisons.
	23d	Discuss implications of the results for practice, policy, and future research.	Discussion - Clinical Implications (Page 24) & Conclusion (Page 4): Detailed recommendations for a personalized, symptom-driven approach and the design of future multi-omic head-to-head trials.
**OTHER INFORMATION**			

Registration and protocol	24a	Provide registration information for the review, including register name and registration number, or state that the review was not registered.	Methods (Page 3, Paragraph 1): Registered in PROSPERO international database under registration number CRD420261380067.
	24b	Indicate where the review protocol can be accessed, or state that a protocol was not prepared.	Methods (Page 3, Paragraph 1): Detailed in text; protocol available dynamically through the PROSPERO platform using the provided registration ID.
	24c	Describe and explain any amendments to information provided at registration or in the protocol.	None reported so far.
Support	25	Describe sources of financial or non-financial support for the review, and the role of the funders or sponsors in the review.	None reported so far.
Competing interests	26	Declare any competing interests of review authors.	None reported so far.
Availability of data, code and other materials	27	Report which of the following are publicly available and where they can be found: template data collection forms; data extracted from included studies; data used for all analyses; analytic code; any other materials used in the review.	None reported so far.

Appendix 16：Safety and Adverse Events

Table S16.1 Summary of Reported Adverse Events Across Included Trials

Study	Assigned Node	Reported Adverse Events (AEs)	Serious Adverse Events (SAE)
Scheperjans	FMT	Mild bloating, transient diarrhea, abdominal discomfort	None
Cheng Y	FMT	Mild abdominal cramps, nausea	None
DuPont	FMT	Transient gastrointestinal discomfort, self-limiting low-grade fever	None
Zhang R	FMT	Mild abdominal pain	None
Wang J	FMT	Mild, self-limiting diarrhea	None
Figura	FMT	Mild bloating	None
Liu J	FMT	No AEs reported	None
Leta V	Probiotics	Mild, transient bloating	None
Mehrabani	Synbiotics	Mild abdominal discomfort	None
Ibrahim	Probiotics	Mild bloating	None
Wang SY	Probiotics	Mild gastrointestinal discomfort	None

Appendix 17:

Table S17.1:Key Efficacy Findings, Clinical Implications, and Trial Limitations

Dimension / Domain	Domain Key Efficacy Findings (What We Found)	Clinical Implications (What It Means)	Methodological Limitations (Crucial Caveats)
Non-Motor Symptoms (SBM Frequency & Constipation)	Oral probiotics achieved a statistically significant increase in weekly SBM frequency (MD = 1.93, 95% CI: 0.23–3.64) compared with placebo.	Oral probiotics may be considered as an adjunctive option for PD-associated constipation; interpretation should reflect the Moderate certainty of evidence.	High clinical heterogeneity exists regarding probiotic strains, taxa, prebiotics, and specific viable dosing regimens.
	Subgroup analyses confirmed consistent efficacy for both probiotics and synbiotics under oral delivery.		
Central Neurological Motor Symptoms (MDS-UPDRS III Score)	In the primary analysis, no active gut microbiota-targeted intervention (FMT, probiotics, synbiotics) demonstrated statistically significant motor improvements compared with placebo	SUCRA probability rankings for FMT or synbiotics represent mathematical tendencies rather than confirmed clinical superiority.	The motor symptom network is highly sensitive to trial-level bias.
	Sensitivity analysis of low-risk trials revealed a tentative therapeutic signal for FMT (MD = -3.04, 95% CI: -6.04 to -0.04).	General gut microbiota-targeted treatment cannot currently be recommended as a standard therapy for central motor control.	Sparse network geometry with a critical deficit of direct head-to-head active comparisons.
Quality of Life (HRQoL via PDQ-39)	No active interventions achieved statistically significant improvements in PDQ-39 scores compared with placebo.	Microecological interventions may provide subtle, multi-dimensional benefits (e.g., sleep, anxiety) that conventional care fails to address.	PDQ-39 measurements carry inherent investigator-administered subjectivity.
	However, active microbial therapies showed significant reductions in PDQ-39 scores when compared exclusively to Routine Care.		Sparse data networks prevent stable multi-dimensional evaluation of secondary endpoints.
Safety & Tolerability Profiles	Reported adverse events with probiotics and synbiotics were mainly mild and transient.	Comparative and long-term safety remains uncertain because adverse-event reporting was incomplete.	Adverse-event details were inconsistently or incompletely reported across the included trials.
	FMT introduced a higher frequency of transient, self-limiting adverse events (diarrhea, bloating, transient fever).		

Figure S17.1:Conceptual summary framework of the network meta-analysis.

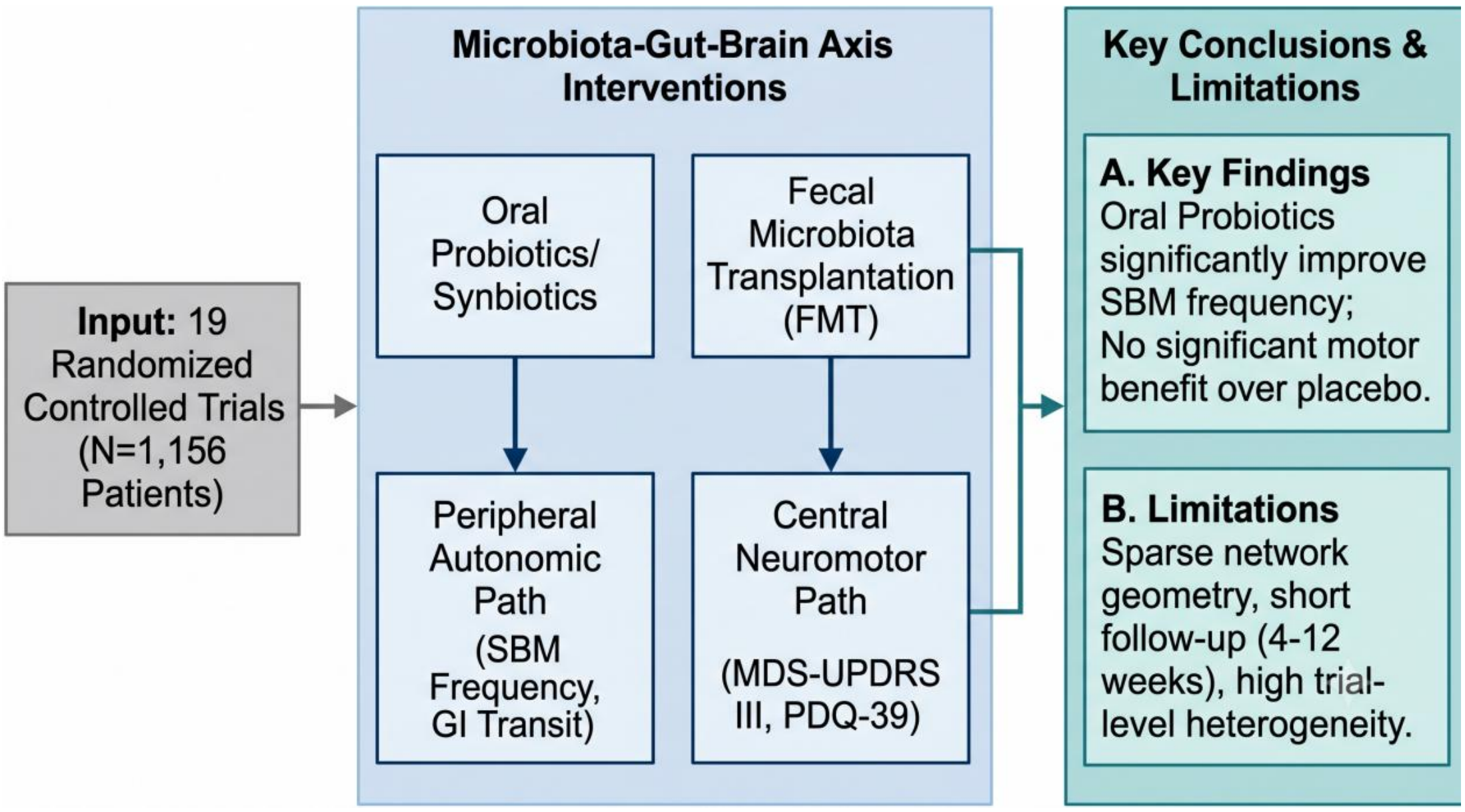

Supplement: Supplementary file 1 [file Data_Sheet_1.pdf]
